# Simple yttrium salts as highly active and controlled catalysts for the atom-efficient synthesis of high molecular weight polyesters†

**DOI:** 10.1039/d2sc02745c

**Published:** 2022-08-24

**Authors:** Zachary A. Wood, Mikiyas K. Assefa, Megan E. Fieser

**Affiliations:** Department of Chemistry, University of Southern California Los Angeles California 90089 USA; Wrigley Institute for Environmental Studies, University of Southern California Los Angeles California 90089 USA fieser@usc.edu

## Abstract

The ring-opening copolymerization (ROCOP) of epoxides and cyclic anhydrides is a promising route to sustainable aliphatic polyesters with diverse mechanical and thermal properties. Here, simple yttrium chloride salts (YCl_3_THF_3.5_ and YCl_3_·6H_2_O), in combination with a bis(triphenylphosphoranylidene)ammonium chloride [PPN]Cl cocatalyst, are used as efficient and controlled catalysts for ten epoxide and anhydride combinations. In comparison to past literature, this simple salt system exhibits competitive turn-over frequencies (TOFs) for most monomer pairs. Despite no supporting ligand framework, these salts provide excellent control of dispersity, with suppression of side reactions. Using these catalysts, the highest molecular weight reported to date (302.2 kDa) has been obtained with a monosubstituted epoxide and tricyclic anhydride. These data indicate that excellent molecular weight control and suppression of side reactions for ROCOP of epoxides and cyclic anhydrides can coincide with high activity using a simple catalytic system, warranting further research in working towards industrial viability.

## Abstract 2

Two simple yttrium salts, YCl_3_THF_3.5_ and YCl_3_·6H_2_O, are highly active and controlled catalysts for the perfectly alternating ring-opening copolymerization of epoxides and cyclic anhydrides.

## Graphical abstract

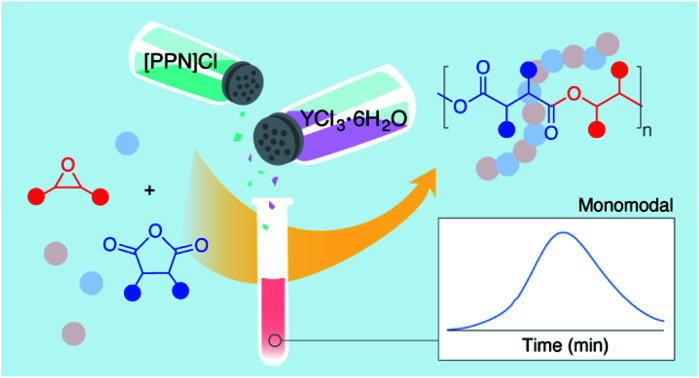

## Introduction

**Fig. 1:**
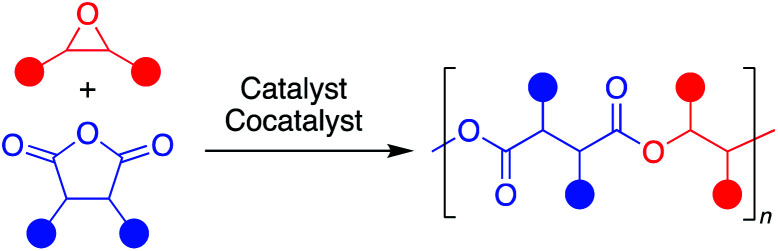
Representative perfectly alternating ring-opening copolymerization of epoxides and cyclic anhydrides.

Aliphatic polyesters are a promising class of plastics that can be synthesized from renewable sources and have the potential to be biodegradable and/or chemically recyclable, thus working away from petroleum sources while also providing more sustainable end-of-life solutions.^1^ While ring-opening polymerization (ROP) of cyclic esters has led to commercialized and chemically recyclable polyesters, limitations in monomer diversity restricts the polymer properties available.^2,3^ The perfectly alternating ring-opening copolymerization (ROCOP) of epoxides and cyclic anhydrides is a promising route to sustainable polyesters with a wider range of physical properties (Fig. 1).^4,5^

**Fig. 2:**
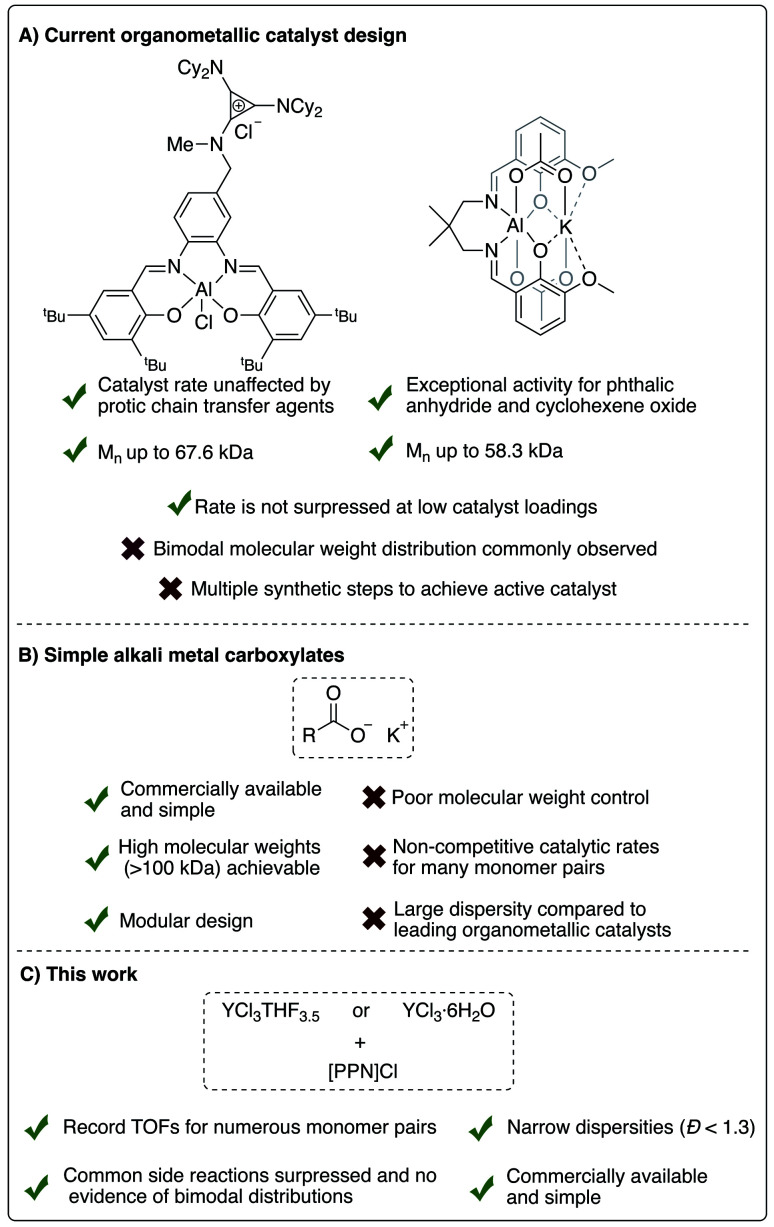
Comparison of current successful catalyst designs for the ROCOP of epoxides and cyclic anhydrides.

To address commercial viability of this process, catalytic methods must be inexpensive, rapid, versatile and controlled. For this polymerization, homopolymerization of epoxides, inter- and intramolecular transesterification, and epimerization are common side reactions that inhibit the cost-effective synthesis of the target polyesters with high molecular weights and controlled dispersities.^5^ Elaborate ligand design for transition metal and main group catalysts and use of a cocatalyst have enabled high rates of polymerization and successful synthesis of moderately high molecular weight polymers (Fig. 2a).^6–11^ However, despite well-thought-out and implemented ligand design throughout the literature, a “one-size-fits-all” catalyst that can polymerize a wide range of monomers with fast rates, excellent molecular weight control, absence of side reactions and produce well-defined high molecular polymers has yet to be established.

Towards more industrially viable catalytic systems, the use of simple salts has been explored by researchers the field. In fact, some of the earliest forays into this ROCOP employed simple metal salt catalysts, such as halides and alkoxides of zinc, magnesium and aluminum, yet these catalysts were deemed sluggish and uncontrolled due to polyether formation in addition to broad dispersities.^12–14^ This is unsurprising given the absence of an ancillary ligand framework and cocatalyst in these examples. Nevertheless, more efficient and versatile simple salt catalysts have recently been identified. For example, alkali metal carboxylates have recently been shown to be effective for the ROCOP of numerous epoxides and cyclic anhydrides, as well as the ring-opening polymerization of cyclic esters.^15–17^ Simple magnesium alkoxide salts have also been shown to produce nearly perfectly alternating aliphatic polyesters *via* ROCOP.^18,19^ However, the rate and polymer dispersity were often lacking in comparison to organometallic catalysts in both examples for many of the monomer pairs studied (Fig. 2b). More recently, our group showed that simple yttrium trisalkyl species, in the presence of a cocatalyst, can polymerize 1,2-butylene oxide and phthalic anhydride with good control (*Đ* < 1.30).^20^ Yttrium trichloride, in the presence of neutral donors, has also been shown to polymerize cyclic esters, albeit with sub-optimal control of dispersity.^21^ Inspired by these results, we endeavored to investigate the catalytic activity of simple yttrium salts for ROCOP.

Herein, we report that the YCl_3_THF_3.5_ and bis(triphenylphosphoranylidene)ammonium chloride ([PPN]Cl) catalyst/cocatalyst pair are surprisingly highly active and controlled for the ROCOP of a variety of epoxides and cyclic anhydrides, Fig. 2c. Moreover, the YCl_3_·6H_2_O hydrate salt also shows excellent rates and control (*Đ* < 1.30) of the target ROCOP with no evidence of bimodal distributions despite the reaction taking place exposed to standard atmosphere. Between the two salts, record TOFs were identified for eight monomer pairs spanning monosubstituted and disubstituted epoxides and monocyclic, bicyclic and tricyclic anhydrides. In addition, the highest recorded polymer molecular weight known to date for this ROCOP is realized using the YCl_3_THF_3.5_/[PPN]Cl catalyst pair. Many of these attributes are sought after in highly air/moisture unstable metal complexes that require multiple synthetic steps, emphasizing the relevance of such simple yet efficient catalyst systems in advancing this polymerization's industrial viability.

## Results/discussion

### Catalyst optimization

The monocyclic epoxide 1,2-butylene oxide (BO) and the tricyclic carbic anhydride (CPMA) were the initial monomers of interest to investigate catalyst efficacy against undesirable epimerization side reactions. [PPN]Cl was used as a cocatalyst, as it has been shown to significantly enhance the polymerization rate and control (in regard to dispersity and side reactions) of many catalysts in the literature.^5,6^ While anhydrous YCl_3_ and [PPN]Cl were able to catalyze the ROCOP of BO and CPMA at 60 °C for five hours (Table S1,† entries 1–3), the yield of the 5 hour polymerization reactions was inconsistent. This inconsistency may be due to the large error of weighing small catalyst quantities (∼2 mg) of a static-prone solid inside a glovebox or variable YCl_3_ solubilization timeframes. Notably, no evidence of homopolymerization of BO or epimerization of the polymer was observed, which has previously been seen with other simple salts.^22–24^

In order to address inconsistency due to solubility and weighing challenges of YCl_3_, stock solutions were first made to solubilize the catalyst prior to polymerization. However, presence of polyether was identified in these stock solutions by the time the YCl_3_ was entirely solubilized. This is unsurprising given literature precedence for simple halide salts being identified as active catalysts for the homopolymerization of epoxides.^22–24^ Instead, YCl_3_THF_3.5_ was selected as a promising alternative that has greater solubility and higher molecular weight than YCl_3_, which were anticipated to improve reproducibility.^25^ Catalysis with YCl_3_THF_3.5_, in the presence of [PPN]Cl, resulted in not only more consistent results, but also an appreciable TOF of 19 h^−1^ at 60 °C, while maintaining excellent molecular weight control and absence of side reactions (Table 1, entry 1). Heating to 110 °C led to a dramatic increase in TOF from 19 h^−1^ to 402 h^−1^ (Table 1, entry 2). While single point measurements do not provide a complete picture of polymerization rate, they are the most common measure of TOF for this polymerization in the literature.

**Table 1:** Catalytic reactions for the copolymerization of BO and CHO with CPMA with an YCl_3_THF_3.5_ catalyst and [PPN]Cl cocatalyst^a^

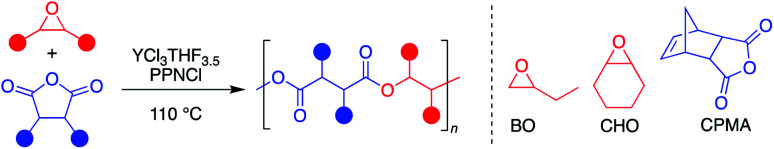									
Entry	Epoxide	Time	Yield^b^ (%)	TOF^c^ (h^−1^)	Ester^d^ (%)	Epim^e^ (%)	^Theor^ *M*_n_ f (kDa)	^Exp^ *M*_n_ g (kDa)	*Đ* g
1^h^^,^^i^	BO	5 h	93(2)	19(1)	>99	<1	5.3	6.3(6)	1.04(2)
2^h^	BO	10 min	67(1)	402(3)	>99	<1	3.8	4.8	1.08(6)
3^j^	BO	10 min	24	144	>99	<1	—	—	—
4^k^	BO	10 min	7	42	>99	<1	—	—	—
5	BO	20 min	>99	>300	>99	19	5.9	11.9	1.08
6	BO	24 h	>99	—	>99	>99	5.8	11.1	1.28
7	CHO	10 min	63	378	>99	<1	3.9	4.0	1.10
8	CHO	24 h	>99	—	>99	21	6.8	7.5	1.48

a [Epoxide] : [CPMA] : [YCl_3_THF_3.5_] : [[PPN]Cl] was 500 : 100 : 1 : 1 at 110 °C, unless otherwise noted.

b Determined using ^1^H NMR spectra of crude reaction mixtures, comparing the conversion of CPMA monomer to polymer.

c Defined as mol CPMA consumed/(mol YCl_3_THF_3.5_*x* h).

d Determined using ^1^H NMR spectra of crude reaction mixtures, comparing the polyether signal to a polyester signal.

e Determined using ^1^H NMR spectra of purified polymers as described previously: epim (%) = {2 × *A*_2.7 ppm_/(*A*_6.0–6.5 ppm_)} × 100.^26^

f Calculated for 4 initiating chlorides.

g Identified by gel permeation chromatography (GPC), using a Wyatt MALS detector.

h Reactions done in triplicate to show reproducibility. Averages and standard deviation shown, individual reactions can be found in Table S1.

i Reaction done at 60 °C.

j [PPN]Cl was not used.

k YCl_3_THF_3.5_ was not used.

**Fig. 3:**
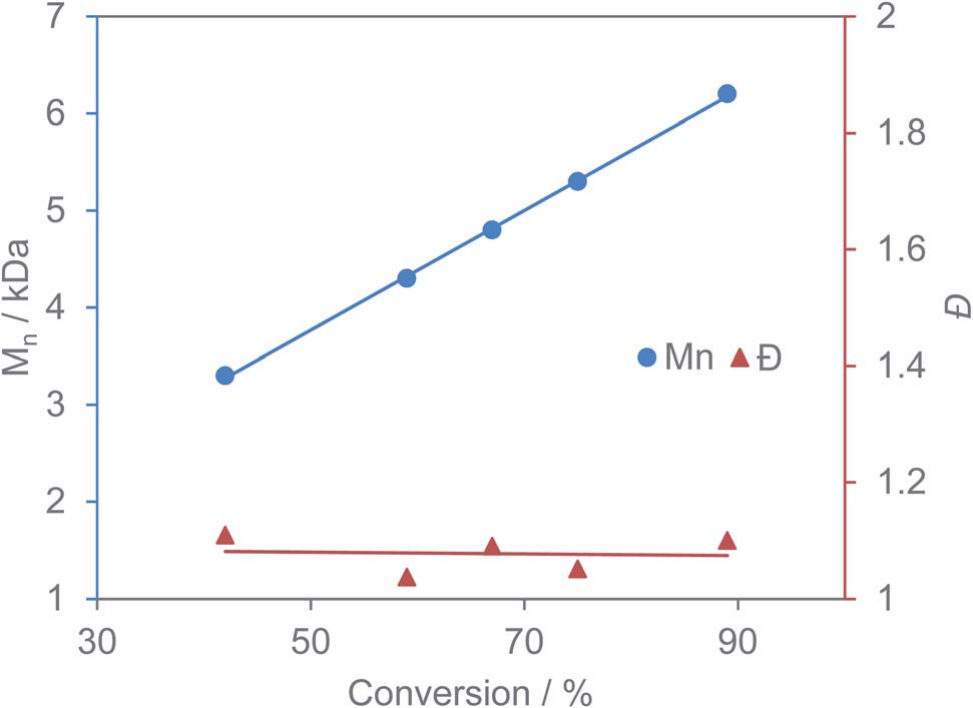
Plot of change in polymer molecular weight and dispersity with increasing anhydride conversion for BO/CPMA using YCl_3_THF_3.5_/[PPN]Cl catalyst pair.

Excellent molecular weight control and complete suppression of side reactions is still maintained at 110 °C. This is the highest TOF reported for the copolymerization of BO/CPMA, despite both YCl_3_THF_3.5_ and [PPN]Cl being simple salts with no supporting ligand frameworks. While our catalyst system is slower at 60 °C in comparison to literature examples, YCl_3_THF_3.5_/[PPN]Cl maintains control of dispersity and polyester linkages at 110 °C with no signs of catalyst deactivation (hence the higher TOF). This highlights that catalytic conditions are just as important as catalyst design. This suggests that it is important to compare catalysts at identical conditions and to compare optimized conditions for each catalyst. Time point reactions revealed a linear increase in polymer molecular weight with increasing anhydride conversion while maintaining dispersities under 1.11, indicating living polymerization (Fig. 3). Control reactions using just YCl_3_THF_3.5_ or [PPN]Cl led to severely lower yields (Table 1, entries 3 and 4, respectively) suggesting the catalyst/cocatalyst pair is needed for efficient catalysis. Finally, the MALDI-TOF mass spectrum of the isolated polymer is consistent with α,ω-Cl,OH and α,ω-Cl,OPPN end groups and a perfectly alternating polyester microstructure, in agreement with the ^1^H NMR spectroscopic data (Fig. S63†).

Although epimerization does occur soon after the copolymerization of BO and CPMA is complete (Table 1, entry 5), transesterification is still suppressed as the dispersity of the resulting polymer remains very low. In this case, an experimental molecular weight twice that of the theoretical is observed. This could be attributed to chain end coupling, previously proposed by Coates and coworkers.^4^ In their case, this coupling was hypothesized to be mediated by nucleophillic alkoxide termini that intermolecularly displace chloride initiators *via* an SN_2_ type attack, resulting in a second, higher molecular weight distribution in the Gel Permeation Chromatography (GPC) trace for reactions run for several hours past full conversion. Curiously, in our case, GPC analysis of polymers isolated from reactions run only minutes past full conversion (20 min total) finds a monomodal molecular weight distribution (Table 1, entry 5 and Fig. S26†). Further heating of the reaction for 24 h still led to a monomodal molecular weight distribution and a molecular weight nearly twice that of the expected, while the dispersity remained relatively low (Table 1, entry 6 and Fig. S27†).

**Scheme 1:**
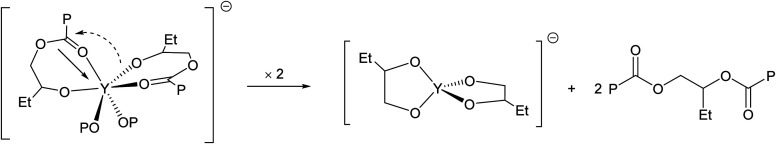
Possible mechanism of post-polymerization transesterification (P = polymer chain).

To investigate this unique behavior, we monitored the polymerization of BO/CPMA by inverse-gated ^13^C NMR spectroscopy using a 1 : 1:50 : 250 YCl_3_THF_3.5_ : [PPN]Cl : CPMA : BO feed ratio at 110 °C (Fig. S17†). Interestingly, the *in situ*^13^C NMR spectrum before full anhydride consumption (5 min reaction time) revealed two environments at 73.93 and 74.25 ppm attributable to the methine carbon of the yttrium-bound alkoxide end group. Given the absence of ancillary ligands on the yttrium, we speculate this may be due to a *κ*^2^ metal coordination at the kinetic chain-end, which desymmeterizes the two epoxide enantiomers (Scheme 1). Upon complete consumption of the CPMA (15 min), these peaks disappear, and new environments at 74.64 and 74.86 ppm are observed, along with a rapid onset of epimerization. Integration of these new peaks relative to the methine environment of the non-terminating epoxides normalized against a 1,4-bis(trimethylsilyl) benzene internal standard (1 : 19 relative ratio, respectively), and comparison with that of the former (1 : 9 relative ratio), is consistent with doubling of the degree of polymerization. This result is also in good agreement with the MALDI-TOF-MS data obtained for BO/CPMA copolymers isolated from reactions run past conversion, which implicated α,ω-Cl,Cl end groups (Fig. S65†). Based on these data, a possible coupling mechanism might involve a rapid post-polymerization transesterification reaction *via* intramolecular chain-end condensation that releases a dormant polyester and anionic yttrium alkoxides (Scheme 1). We note that this mechanism has precedence in non-hydrolytic sol–gel processes that employ molecular metal alkoxides and carboxylates to generate metal/mixed-metal oxides and/or species with a mixture of oxide and alkoxide anions under anhydrous conditions at temperatures similar to that used in this reaction.^27–29^

Extending reactivity studies to include cyclohexene oxide (CHO), a disubstituted epoxide that is more difficult to ring-open, still led to one of the highest TOFs reported for its copolymerization with CPMA (378 h^−1^), while also maintaining excellent molecular weight control (Table 1, entry 7). Akin to reactions with BO, no side reactions were observed during polymerization. Interestingly, unlike the reaction with BO, doubling of the experimental molecular weight was not observed when heating for 24 h (Table 1, entry 8). Consistent with this finding, MALDI-TOF spectra for copolymers isolated from this reaction revealed α,ω-Cl,OH end groups, indicating coupling *via* the abovementioned mechanism hasn't occurred. Further, the *in situ*^13^C NMR spectrum for CHO/CPMA copolymerization (Fig. S18†) revealed a singlet at 77.65 ppm attributable to the terminating CHO methine environment, that is sustained past full anhydride conversion with no significant changes in its relative integration values. This result may be due to deterrence of *κ*^2^ interactions in the case of CHO on account of its greater steric constraints. If true, the observed coupling for BO may be favored by such interactions that bring two polymer chain ends in close proximity to each other. This is also supported by the fact that there are no ancillary ligands on the yttrium catalyst used, allowing for coordination of multiple polymer chains at once.

### High molecular weight polymers

One main challenge in the ROCOP of epoxides and cyclic anhydrides is the synthesis of high molecular weight polyesters, which is paramount to fully realize their industrial applications in areas such as packaging and tissue engineering. This challenge can stem from strong dilution effects or prolonged reaction times at elevated temperatures, both of which can lead to catalyst deactivation. Indeed, there are only a handful reports to date that have achieved polyester molecular weights greater than 100 kDa using this copolymerization (Table S5†), and most of these cases afford molecular weights much lower than the theoretical.^15,30–33^

When considering the plausibility of synthesizing high molecular weight polyesters using our catalyst system, we envisioned the abovementioned coupling mechanism could be exploited to generate high molecular weight polymers using much lower monomer to catalyst feed ratios than what would be nominally required. Thus, we targeted the BO/CPMA monomer combination for low catalyst loading reactions using the YCl_3_THF_3.5_/[PPN]Cl catalyst pair to reach high molecular weights.

We first conducted polymerizations at a 0.25 mol% catalyst loading (based on the anhydride) to gauge the TOF dependence on feed ratio (Table 2, entry 1). As would be expected for a binary system, the TOF for this reaction decreased to 62 h^−1^. GPC analysis of the resulting polymer revealed good agreement between theoretical and experimental molecular weights, and preservation of polymerization control as evidenced by a narrow dispersity. We next investigated if post-polymerization transesterification also occurs at this catalyst loading. Gratifyingly, running the polymerization past full conversion yielded a polymer with an experimental *M*_n_ nearly twice that of the theoretical (Table 2, entry 2). Inspired by this promising result, we conducted polymerizations at a very low catalyst loading of 0.04 mol%, which resulted in full conversion of the anhydride and a 90% polyester content (Table 2, entry 3). Interestingly, we don't see formation of the trans diester polymer in this reaction, which may be due to the much larger monomer to catalyst feed ratio. GPC analysis of the isolated polymer revealed an unprecedented molecular weight of 302.2 kDa with a moderate dispersity of 1.62, which is consistent with the anticipated post-polymerization transesterification. To our knowledge, this is the highest molecular weight reported for polyesters synthesized by ROCOP of epoxides and cyclic anhydrides. Notably, the GPC trace of the high molecular weight polymer did not exhibit the distinct bimodal distribution that is commonly seen in low catalyst loading ROCOP polymerizations,^7,8,11^ which has previously been ascribed to presence of adventitious water (Fig. S56†).

**Table 2:** Low catalyst loading polymerization reactions^a^

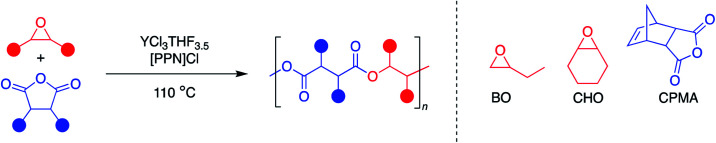										
Entry	Epoxide	Loading^b^ (mol%)	Time	Yield^c^ (%)	TOF^d^ (h^−1^)	Ester^e^ (%)	Epim^f^ (%)	^Theor^ *M*_n_ g (kDa)	^Exp^ *M*_n_ h (kDa)	*Đ* h
1	BO	0.25	5 h	78	62	>99	<1	17.9	20.2	1.29
2	BO	0.25	8 h	>99	>50	>99	5	22.6	41.0	1.74
3	BO	0.04	10 d	>99	>10	90	<1	144.4	302.2	1.62
4	CHO	0.25	2.5 h	72	115	>99	<1	19.7	25.9	1.34
5	CHO	0.04	5 d	>99	>20	62	<1	195.4	139.4	1.34

a [Epoxide] : [CPMA] was 5 : 1 at 110 °C.

b Based on the anhydride.

c Determined using ^1^H NMR spectra of crude reaction mixtures, comparing the conversion of CPMA monomer to polymer.

d Defined as mol CPMA consumed/(mol catalyst *x* h).

e Determined using ^1^H NMR spectra of purified polymers, comparing the polyether signal to a polyester signal.

f Determined using ^1^H NMR spectra of purified polymers as described previously: epim (%) = {2 × *A*_2.7 ppm_/(*A*_6.0–6.5 ppm_)} × 100.^26^

g Calculated for 4 initiating chlorides.

h Identified by gel permeation chromatography (GPC), using a Wyatt MALS detector.

There is a general consensus in the ROCOP literature that disubstituted epoxides are much harder to polymerize to high molecular weights than their monosubstituted congeners.^5^ In this regard, given their high activity towards copolymerization of tricyclic anhydrides, we targeted to prepare high molecular weight CHO/CPMA polyesters using the YCl_3_THF_3.5_/[PPN]Cl catalyst pair. Accordingly, copolymerization of CHO and CPMA was carried out at a 0.25 mol% catalyst loading, which resulted in a TOF of 115 h^−1^ and a corresponding increase in molecular weight, along with maintenance of good polymerization control and suppression of side reactions (Table 2, entry 4). Subsequent reactions using 0.04 mol% catalyst loading afforded a remarkably high polymer molecular weight of 139.4 kDa with a narrow dispersity of 1.34 (Table 2, entry 5). This *M*_n_ is amongst the highest reported for polyesters synthesized by ROCOP. While ^1^H NMR spectroscopy analysis did indicate a noticeably increased polyether content, GPC analysis of the isolated polymer revealed a well-defined monomodal distribution and an experimental molecular weight that is in reasonable agreement with theoretical assuming four chloride initiators (Fig. S58†), illustrating good polymerization control given the low catalyst loading employed. One flaw for this catalytic system is that catalyst efficiency decreases significantly with a decreased catalyst loading, as expected with a bimolecular catalyst system.^9^ Additionally, polyether formation appears to compete with ROCOP at lower catalyst loadings. Despite these limitations, the demonstrated access to high molecular weight polyesters derived from disubstituted epoxides *via* ROCOP represents a significant advance towards realizing commercial viability of this synthetic method for aliphatic polyesters.

To probe the dependence of polymer thermal properties on molecular weight, differential scanning calorimetry (DSC) was employed to measure the glass transition temperatures (*T*_g_s) of the different molecular weight polyesters synthesized using the YCl_3_THF_3.5_/[PPN]Cl pair. DSC analysis of the BO/CPMA copolymers with *M*_n_ values ranging from 4.8 to 41.0 kDa revealed linear dependence of *T*_g_ on 1/*M*_n_ (*T*_g_ = 46–55 °C; Fig. S67 and S68†).^34–36^ The *T*_g_s for the CHO/CPMA congeners with similar *M*_n_ values (4.0 and 25.9 kDa) were comparatively higher at 82 and 88 °C; respectively, consistent with the greater rigidity of the disubstituted epoxide CHO (Fig. S69†). On the other hand, the *T*_g_s of the high molecular weight BO/CPMA and CHO/CPMA copolymers were comparatively underwhelming at 53 and 116 °C, respectively. The depression of these *T*_g_ values is consistent with their polyether content, as poly(1,2-butylene oxide) and poly(cyclohexene oxide) have *T*_g_s of −60 °C and 75 °C, respectively.^37,38^ Given their exceptionally high *M*_n_ values, these copolymers may also have already reached their maximum glass transition temperature. Notably, the observation of a single *T*_g_ value for both copolymers is indicative of statistical distribution of the ether linkages across the polyester backbone. Finally, endothermic and exothermic peaks assignable to melting and crystallization temperatures were absent in all DSC traces, confirming all tested copolymers are amorphous.^39^

### Monomer scope

Since YCl_3_THF_3.5_ and [PPN]Cl showed high activity for both monosubstituted and disubstituted epoxides with a tricyclic anhydride, expansion of the monomer scope was the next area of interest (Table 3). Using the same two epoxides, BO and CHO, the anhydride scope was expanded to include monocyclic and bicyclic anhydrides. In particular, succinic anhydride (SA) has been identified as a high-value biomass-based chemical.^12^ Phthalic anhydride (PA) is the most-common bicyclic anhydride used for ROCOP in the literature.^5^ Glutaric anhydride (GA) represents a monocyclic anhydride with a lower ring strain that can be obtained from biomass.^40^ Cyclohexane anhydride (CHA) was used as a non-aromatic analogue of phthalic anhydride.

**Table 3:** Catalytic reactions for the copolymerization of BO and CHO and several anhydrides with an YCl_3_THF_3.5_ catalyst and [PPN]Cl cocatalyst^a^

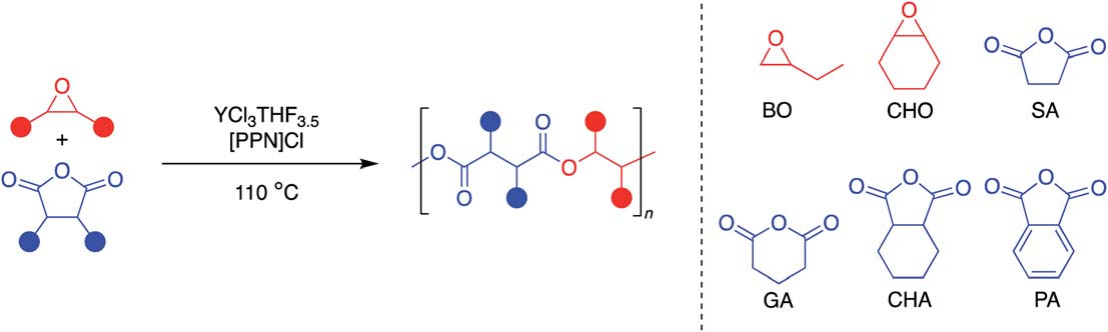									
Entry	Epoxide	Anhydride	Time (min)	Yield^b^ (%)	TOF^c^ (h^−1^)	Ester^d^ (%)	^Theor^ *M*_n_ e (kDa)	^Exp^ *M*_n_ f (kDa)	*Đ* f
1	BO	PA	50	85	102	>99	4.5	4.5	1.07
2	BO	PA	120	>99	50	>99	5.5	9.0	1.39
3	CHO	PA	50	71	85	96	4.2	5.3	1.13
4	BO	GA	60	96	96	>99	4.3	4.9	1.19
5	BO	GA	120	>99	50	98	4.7	3.3	2.85
6	CHO	GA	30	94	188	>99	4.9	3.8	1.06
7	BO	CHA	40	91	137	>99	5.2	3.1	1.08
8	BO	CHA	120	>99	50	97	5.7	5.6	1.46
9	CHO	CHA	40	82	123	92	5.2	3.2	1.07
10	BO	SA	45	98	131	>99	4.1	2.1^g^	1.40^g^
11	BO	SA	120	>99	50	98	4.3	2.2^g^	1.72^g^
12	CHO	SA	60	83	83	93	4.2	2.0^g^	1.65^g^

a [Epoxide] : [anhydride] : [YCl_3_THF_3.5_] : [[PPN]Cl] was 500 : 100 : 1 : 1 at 110 °C.

b Determined using ^1^H NMR spectra of crude reaction mixtures, comparing the conversion of anhydride to polymer.

c Defined as mol anhydride consumed/(mol YCl_3_THF_3.5_*x* h).

d Determined using ^1^H NMR spectra of crude reaction mixtures, comparing the polyether signal to a polyester signal.

e Calculated for 4 initiating chlorides.

f Identified by gel permeation chromatography (GPC), using a Wyatt MALS detector.

g Identified by a Wyatt refractive index (RI) detector.

All eight monomer combinations could be polymerized to high conversions within an hour at 110 °C (Table 3). For most monomer combinations, the GPC data indicate low dispersities and good agreement with theoretical molecular weights calculated for four initiating chlorides, while combinations with SA exhibited the highest dispersities. No epoxide homopolymerization was observed for all monomer combinations except for CHO/PA, CHO/CHA and CHO/SA, which exhibited 4, 8 and 7% polyether linkages, respectively. TOFs for the bicyclic and monocyclic anhydride monomers were all lower than that for the tricyclic anhydride, CPMA. In addition, TOFs were consistently higher with the BO monomer, with the exception of combinations with GA, which were faster with CHO. Even still, the YCl_3_THF_3.5_/[PPN]Cl catalyst/cocatalyst pair has competitive TOFs for several of the monomer combinations studied. Comparisons to the top catalysts in the literature are discussed below.

### Hydrated salt catalysts

Since the YCl_3_THF_3.5_ salt was able to catalyze the ROCOP of epoxides and cyclic anhydrides with good molecular weight control, we were curious if the hydrate salt (YCl_3_·6H_2_O) could also be active. This would make the catalyst even cheaper and easier to use in comparison to the anhydrous YCl_3_THF_3.5_; however, it was anticipated that the water solvates present would serve as bifunctional chain transfer agents, causing a bimodal molecular weight distribution.^5,6^ Surprisingly, YCl_3_·6H_2_O and [PPN]Cl were not only highly active for the copolymerization, but also showed good control of dispersity (*Đ* < 1.3) for all the monomer combinations studied with YCl_3_THF_3.5_ (Table 4). As noted by many in the field of this polymerization, adventitious water or diacid/diol impurities can impede the synthesis of well-defined polyesters *via* ROCOP,^5,6^ therefore, all reagents often need to be dried and purified before use to achieve the best results. In this case however, purified monomers were not stored in air-free conditions. In fact, all polymerizations are conducted in open air.

**Table 4:** Catalytic reactions for the copolymerization of BO and CHO and several anhydrides with an YCl_3_·6H_2_O catalyst and [PPN]Cl cocatalyst^a^

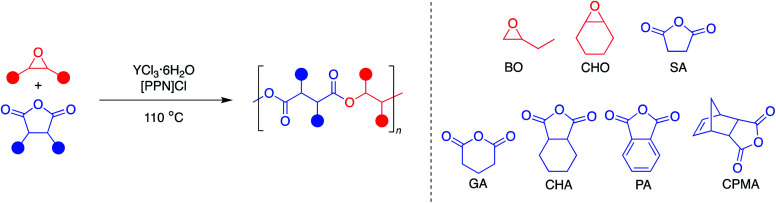										
Entry	Epoxide	Anhydride	Time (min)	Yield^b^ (%)	TOF^c^ (h^−1^)	Ester^d^ (%)	Epim^e^ (%)	^Theor^ *M*_n_ f (kDa)	^Exp^ *M*_n_ g (kDa)	*Đ* g
1^i^	BO	CPMA	30	76(3)	152(6)	>99	<1	1.8	1.8(1)	1.06(1)
2	CHO	CPMA	20	68	204	>99	<1	1.8	1.7	1.09
3	BO	PA	15	87	348	>99	—	2.2	3.1	1.08
4	CHO	PA	5	54	648	>99	—	1.4	2.2	1.03
5	BO	GA	50	>99	>120	>99	—	1.9	1.4	1.19
6	BO	GA	25	57	136	>99	—	—	—	—
7	CHO	GA	25	>99	>240	>99	—	2.0	2.2	1.03
8	CHO	GA	15	43	172	>99	—	—	—	—
9	BO	CHA	15	95	380	>99	—	2.3	2.1	1.03
10	CHO	CHA	15	>99	>400	91	—	2.3	1.4^h^	1.20^h^
11	CHO	CHA	5	93	1116	—	—	—	—	—
12	BO	SA	30	>99	>200	>99	—	1.7	1.8^h^	1.28^h^
13	BO	SA	15	37	148	>99	—	—	—	—
14	CHO	SA	17	91	321	>99	—	1.8	1.4^h^	1.14^h^

a [Epoxide] : [anhydride] : [YCl_3_·6H_2_O] : [[PPN]Cl] was 500 : 100 : 1 : 1 at 110 °C.

b Determined using ^1^H NMR spectra of crude reaction mixtures, comparing the conversion of anhydride to polymer.

c Defined as mol anhydride consumed/(mol YCl_3_·6H_2_O *x* h).

d Determined using ^1^H NMR spectra of crude reaction mixtures, comparing the polyether signal to a polyester signal.

e Determined using ^1^H NMR spectra of purified polymers as described previously: epim (%) = {2 × *A*_2.7 ppm_/(*A*_6.0–6.5 ppm_)} × 100.^26^

f Calculated for 4 initiating chlorides and 6 water chain transfer agents.

g Identified by gel permeation chromatography (GPC), using a Wyatt MALS detector.

h Identified by a Wyatt refractive index (RI) detector.

i Reactions done in triplicate to show reproducibility. Averages and standard deviation shown, individual reactions can be found in Table S1.

GPC analysis of the polymers synthesized using YCl_3_·6H_2_O/[PPN]Cl revealed molecular weights in agreement with 10 active initiators, consistent with that expected for four chlorides and six water chain transfer agents. However, MALDI-TOF-MS end group analysis of the BO/CPMA copolymer indicated majority diol/diacid-initiated telechelics with similar *m*/*z* values (Fig. S64†), indicating that water impurities are a major source of initiator. Correspondingly, the GPC traces also revealed monomodal molecular weight distributions across all the monomers studied (Fig. S47–S53†), in agreement with prior reports for ROCOP catalyst systems containing excess chain transfer agents.^41,42^ For example, Williams *et al.* have previously reported a smooth transition from bi-modal molecular weight distribution to monomodal upon incremental addition of diol chain transfer agent equivalents (5 to 10 equiv.) to the ROCOP of cyclohexene oxide and phthalic anhydride using aluminum *o*-vanillin catalysts.^42^ Finally, there does not appear to be any side reactions present for all monomer combinations, except for the CHO/CHA pair, which exhibited 9% polyether content.

### Understanding the role of catalytic reaction conditions

**Fig. 4:**
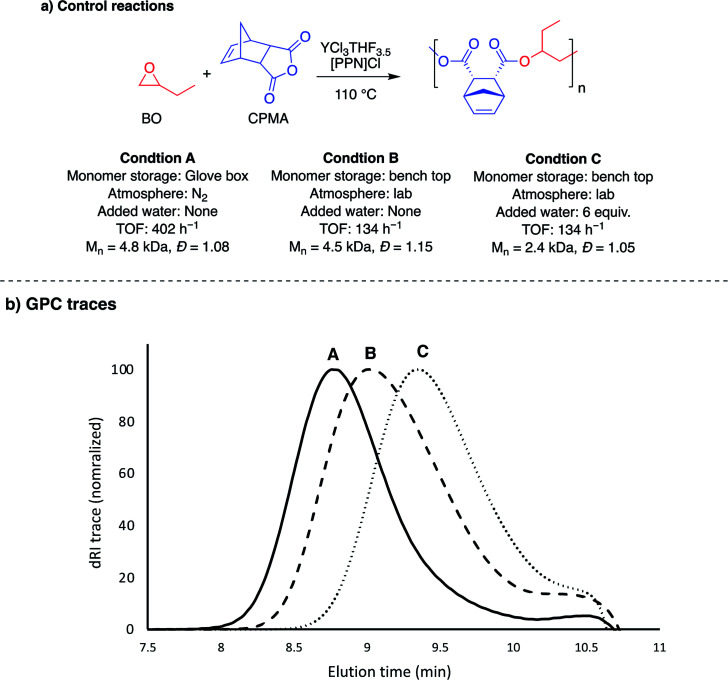
Control reactions performed to understand the impact of reaction conditions on TOF. Condition A is the TOF from Table 1, entry 2. See ESI (Table S3†) for summary of control reactions performed.

The YCl_3_·6H_2_O/[PPN]Cl pair had lower TOFs than the YCl_3_THF_3.5_ for both epoxide combinations with the tricyclic anhydride, CPMA. Interestingly, the hydrate catalyst was significantly faster with all monocyclic and bicyclic anhydride monomers, with both epoxides. Because the YCl_3_THF_3.5_ and YCl_3_·6H_2_O were studied under different reaction conditions, a series of controls using YCl_3_THF_3.5_ were performed to understand the cause for the difference in rates between the two yttrium catalysts (Table S3†). As summarized in Fig. 4, this difference cannot be necessarily attributed to the difference in catalysts alone, as YCl_3_THF_3.5_/[PPN]Cl has a drastic decrease of TOF (402 h^−1^ to 134 h^−1^) for BO/CPMA (Fig. 4, condition A *vs.* B) when using the same conditions as the YCl_3_·6H_2_O/[PPN]Cl reactions (*i.e.*, using monomers stored outside the glove box and preparing reaction exposed to lab atmosphere). This is further emphasized by the measured *M*_n_ value, which is consistent with 4 initiators, suggesting minimal water is present in the monomers stored outside the glove box. Importantly, when 6 equivalents of water is added to a BO/CPMA reaction with YCl_3_THF_3.5_ (Fig. 4, condition C), both the rate and *M*_n_ value are in agreement with those of YCl_3_·6H_2_O/[PPN]Cl. Likewise, when BO/PA stored out of the glove box was copolymerized using YCl_3_THF_3.5_/[PPN]Cl, the TOF nearly doubles, again with *M*_n_ values consistent with four initiators (Table S3,† entry 3). Addition of 6 equivalents of water increased the rate drastically to match that of the hydrated yttrium salt (Table S3,† entry 4). These data suggest that catalytic reaction conditions, such as the presence of (a)protic impurities, play an important role in determining the two yttrium catalysts' relative polymerization rates. For comparison, while not as pronounced, prior ROCOP studies have identified similar effects of small amounts of protic reagents on the activity of simple salt catalysts, such as yttrium isopropoxide and quaternary ammonium salts, which were hypothesized to manifest through reduction of the polymerization induction period.^43–45^ Mechanistic studies to better understand the differences between reaction conditions and catalyst structures are currently underway.

### TOF comparisons

**Fig. 5:**
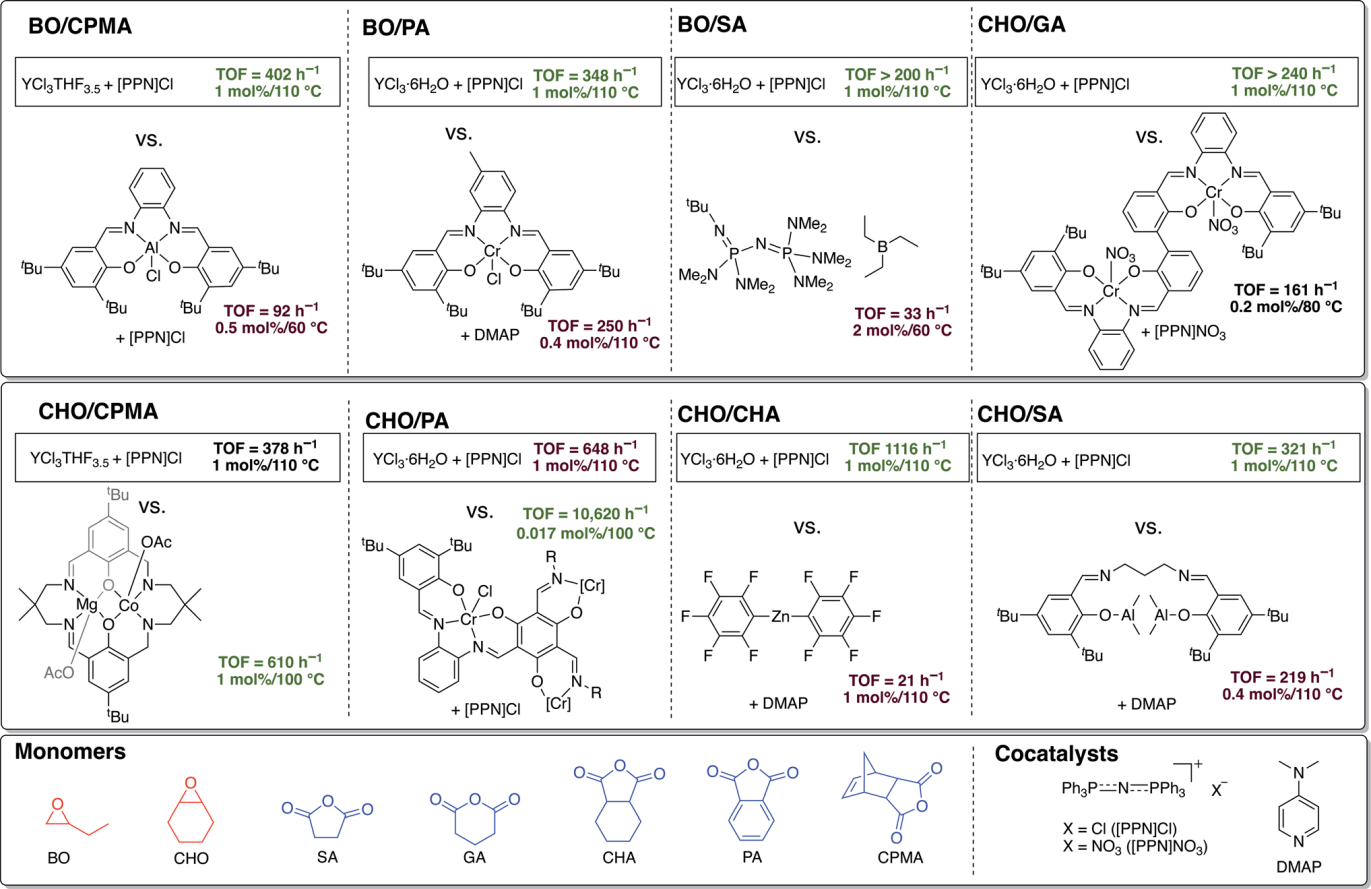
Comparisons of ROCOP TOFs for YCl_3_THF_3.5_ and YCl_3_·6H_2_O with the most active catalysts in the literature for the monomer pairs BO/CPMA,^46^ BO/PA,^47^ BO/SA,^48^ CHO/GA,^49^ CHO/CPMA,^50^ CHO/PA,^51^ CHO/CHA,^52^ CHO/SA.^53^ TOFs for BO/GA and BO/CHA not included as no prior examples could be found.

Given the absence of ancillary ligand frameworks, it is likely that the metal centers on these simple yttrium trichoride catalysts are far more Lewis acidic in comparison to traditional ligand-supported metal catalysts, which may explain their exceptionally high TOFs. In fact, to our knowledge, the air and moisture stable and commercially available salt YCl_3_·6H_2_O has the highest TOF reported in the literature to date for ROCOP of all the monomer pairs studied, except those with CPMA and CHO/PA (Fig. 5).^46–53^ Even though not the quickest catalyst for CPMA and CHO/PA, the YCl_3_·6H_2_O/[PPN]Cl catalyst pair boasts the advantage of being stable to moisture and air in addition to being much simpler than the leading catalysts for these anhydrides. The reverse in the trend of the yttrium catalysts' activity for polymerization of the tricyclic anhydride CPMA warrants future mechanistic studies, particularly in understanding how the sterics of the anhydride rather than epoxide has a greater effect on rate of polymerization.

As discussed above, comparing the TOF (which is a single point measurement) of different catalysts is complicated by the fact that different catalysts use varied optimized conditions. Therefore, to best compare our catalysts to the literature, we performed reactions with our optimized conditions (see tables above) in addition to the optimized conditions that past catalysts used (Table 5). As seen in Table 5, all past catalysts in the literature that report the highest TOF for a given monomer pair use different reactions conditions (*e.g.*, temperature, catalyst loading, epoxide loading, % conversion achieved), all of which affect the observed TOF. This highlights that optimization of conditions is just as important as catalyst design.

**Table 5:** Top ROCOP catalysts in the literature for the monomer pairs chosen for this study (catalysts and cocatalysts shown in Fig. 5)

Entry	Monomers	Loading^a^ (mol%)	[Epoxide] : [anhydride]	Temp (°C)	Time	Polyester (%)	Yield (%)	TOF (h^−1^)
1 (ref. 46)	BO/CPMA	0.5	[1000] : [200]	60	1 h	>99	46	92
2^b^	BO/CPMA	1	[500] : [100]	60	5 h	>99	93	19
3 (ref. 50)	CHO/CPMA	1	[2000] : [100]	100	N/A	N/A	>99	610
4 (ref. 47)	BO/PA	0.4	[250] : [250]	110	1 h	>99	>99	250
5^c^	BO/PA	0.4	[250] : [250]	110	1 h	>99	52	130
6^c^	BO/PA	0.4	[1250] : [250]	110	40 min	>99	>99	375
7 (ref. 51)	CHO/PA	0.017	[30 000] : [6000]	100	20 min	N/A	59	10 620
8 (ref. 48)	BO/SA	2	[200] : [50]	60	1.5 h	>99	98	33
9^c^	BO/SA	2	[200] : [50]	60	1.5 h	>99	10	3
10 (ref. 53)	CHO/SA	0.4	[250] : [250]	110	50 min	94	73	219
11^c^	CHO/SA	0.4	[250] : [250]	110	50 min	>99	73	219
12 (ref. 49)	CHO/GA	0.2	[1000] : [500]	80	3 h	>99	97	161
13^c^	CHO/GA	0.2	[1000] : [500]	80	2.5 h	>99	30	60
14 (ref. 52)	CHO/CHA	1	[100] : [100]	110	4 h	96	82	21
15^c^	CHO/CHA	1	[500] : [100]	110	5 min	>99	93	1116

a Catalyst loading with respect to anhydride.

b Catalysis performed with YCl_3_THF_3.5_/[PPN]Cl.

c Catalysis performed with YCl_3_·6H_2_O/[PPN]Cl.

For example, it has been observed for many catalytic systems that increased epoxide loading leads to an increase in TOF, yet many catalysts cannot use excess epoxide due to competitive polyether formation, such as the case with Cr-salen complex used for the polymerization of BO/PA (entry 4, Table 5). If we apply our catalyst with past literature conditions for BO/PA (entry 5, Table 5), the TOF of YCl_3_·6H_2_O/[PPN]Cl is not the largest. However, if we apply our optimized conditions (entry 6, Table 5), which features the use of excess epoxide, YCl_3_·6H_2_O/[PPN]Cl achieves the highest TOF while still maintaining >99% polyester linkages. This highlights how our catalytic system can use reaction condition that favor a faster rate without sacrificing control in % polyester and dispersity. Further discussion of each monomer pair can be found in the ESI.† Ultimately, given how a change in even one reaction condition can greatly affect TOF, comparisons to the literature are not meant necessarily as a single point measure of catalytic activity, but instead are meant to showcase how robust this catalyst system is under our optimized conditions and how such a simple catalyst system does not sacrifice control in favor of competitive TOFs.

## Conclusion

We report the identification of two simple salt catalysts that, when used in combination with a [PPN]Cl cocatalyst, are highly active for the perfectly alternating copolymerization of epoxides and cyclic anhydrides: YCl_3_THF_3.5_ and YCl_3_·6H_2_O. Both yttrium catalysts are able to efficiently polymerize a wide range of monomer pairs spanning monosubstituted and disubstituted epoxides and monocyclic, bicyclic and tricyclic anhydrides. In most cases, undesirable side reactions were kept minimal. YCl_3_THF_3.5_ was found to be fastest for cases including tricyclic anhydrides, while YCl_3_·6H_2_O was fastest for monocyclic and bicyclic anhydrides. Between the two catalysts studied, they have competitive TOFs for eight out of the ten monomer combinations studied (Fig. 4). Additionally, the YCl_3_THF_3.5_ catalyst affords the highest polymer molecular weight obtained to date with epxoide/anhydride ROCOP using very low sub-stoichiometric monomer equivalents, demonstrating the unprecedented atom economy benefits of this catalyst system.

As YCl_3_·6H_2_O is the more convenient and cost-effective catalyst, being commercially available in large scales, strategies to minimize the total number of initiators in the YCl_3_·6H_2_O/[PPN]Cl catalyst system are currently being devised to access high molecular weights. We also aim to expand the scope of efficient simple metal salt ROCOP catalysis through deployment of a diverse set of chain transfer agents and initiators, a range of Lewis acidic rare earth and transition metals and cocatalysts, and neutral donors with varied functionality.

## Data availability

General considerations for chemicals, metal salts, monomers and solvents, polymerization conditions and results, characterization data and comparisons to other catalysts in the literature.

## Author contributions

Z. A. W. and M. K. A. equally contributed to experimental work, devising experiments, and writing the manuscript. M. E. F. directed the project and helped write the manuscript. All authors discussed the results.

## Conflicts of interest

The authors declare no conflict of interest.

## Supplementary Material

SC-013-D2SC02745C-s001
