# Supplementary material for: Simple yttrium salts as highly active and controlled catalysts for the atom-efficient synthesis of high molecular weight polyesters
Source: Chem Sci. 2022 Aug 24;13(35):10437–47. doi: 10.1039/d2sc02745c (PMC9473511; doi:10.1039/d2sc02745c)
Supplement: SC-013-D2SC02745C-s001 [file SC-013-D2SC02745C-s001.pdf]

Electronic Supplementary Material (ESI) for Chemical Science.
This journal is © The Royal Society of Chemistry 2022

Electronic Supporting Information for

Simple yttrium salts as highly active and controlled catalysts for the atom-efficient synthesis of high molecular weight polyesters

Zachary A. Wood,[†a] Mikiyas K. Assefa,[†a] and Megan E. Fieser*[a,b]

[a]Department of Chemistry, University of Southern California, Los Angeles, California 90089

[b]Wrigley Institute for Environmental Studies, University of Southern California, Los Angeles, California, 90089

**1.**	**General Considerations**	**S3**
	**Fig. S1** Glassware for rare earth metal trichloride drying.	**S5**
**2.**	**General procedures for polymerizations**	**S5**
	**Scheme S1** Conditions for ^{13}C NMR spectroscopic oligomer studies	**S6**
**3.**	**Tabulated Polymerization Data**	**S7**
	**Table S1** Triplicate catalytic reactions	**S7**
	**Table S2** Timepoint data	**S8**
	**Table S3** Control reactions	**S8**
	**Table S4** Catalysis with [PPN]Cl	**S9**
	**Table S5** Prior examples of high molecular weight polyesters	**S9**
**4.**	**Discussion of TOF comparisons from Table 5**	**S10-11**
**5.**	**^{1}H NMR spectra of isolated polymers**	**S12**
	**Fig. S2** ^{1}H NMR spectrum of BO-*alt*-CPMA in $CDCl_3$ (**Table S1**, Entry 7).	**S12**
	**Fig. S3** ^{1}H NMR spectrum of BO-*alt*-CPMA in $CDCl_3$ (**Table 1**, Entry 5).	**S13**
	**Fig. S4** ^{1}H NMR spectrum of BO-*alt*-CPMA in $CDCl_3$ (**Table 1**, Entry 6).	**S14**
	**Fig. S5** ^{1}H NMR spectrum of BO-*alt*-CPMA in $CDCl_3$ (**Table 3**, Entry 3).	**S15**
	**Fig. S6** ^{1}H NMR spectrum of CHO-*alt*-CPMA in $CDCl_3$ (**Table 1**, Entry 7).	**S16**
	**Fig. S7** ^{1}H NMR spectrum of CHO-*alt*-CPMA in $CDCl_3$ (**Table 1**, Entry 8).	**S17**
	**Fig. S8** ^{1}H NMR spectrum of CHO-*alt*-CPMA in $CDCl_3$ (**Table 3**, Entry 5).	**S18**
	**Fig. S9** ^{1}H NMR spectrum of BO-*alt*-PA in $CDCl_3$ (**Table 4**, Entry 1).	**S19**
	**Fig. S10** ^{1}H NMR spectrum of CHO-*alt*-PA in $CDCl_3$ (**Table 4**, Entry 2).	**S20**
	**Fig. S11** ^{1}H NMR spectrum of BO-*alt*-GA in $CDCl_3$ (**Table 4**, Entry 3).	**S21**
	**Fig. S12** ^{1}H NMR spectrum of CHO-*alt*-GA in $CDCl_3$ (**Table 4**, Entry 4).	**S22**
	**Fig. S13** ^{1}H NMR spectrum of BO-*alt*-CHA in $CDCl_3$ (**Table 4**, Entry 5).	**S23**
	**Fig. S14** ^{1}H NMR spectrum of CHO-*alt*-CHA in $CDCl_3$ (**Table 4**, Entry 6).	**S24**
	**Fig. S15** ^{1}H NMR spectrum of BO-*alt*-SA in $CDCl_3$ (**Table 4**, Entry 7).	**S25**
	**Fig. S16** ^{1}H NMR spectrum of CHO-*alt*-SA in $CDCl_3$ (**Table 4**, Entry 8).	**S26**

	**^{13}C NMR Spectroscopic Oligomer Studies**	**S27**
	**Fig. S17** Oligomer study of BO-*alt*-CPMA	**S27**
	**Fig. S18** Oligomer study of CHO-*alt*-CPMA	**S28**
**6.**	**GPC Data**	**S29**
	**Fig. S19** GPC traces of **Table S1**, Entry 13 and THF blank overlayed.	**S29**
	**Fig. S20** GPC traces corresponding to polymerization in **Table S1**, Entry 4.	**S29**
	**Fig. S21** GPC traces corresponding to polymerization in **Table S1**, Entry 5.	**S30**
	**Fig. S22** GPC traces corresponding to polymerization in **Table S1**, Entry 6.	**S30**
	**Fig. S23** GPC traces corresponding to polymerization in **Table S1**, Entry 7.	**S31**
	**Fig. S24** GPC traces corresponding to polymerization in **Table S1**, Entry 8.	**S31**
	**Fig. S25** GPC traces corresponding to polymerization in **Table S1**, Entry 9.	**S32**
	**Fig. S26** GPC traces corresponding to polymerization in **Table 1**, Entry 5.	**S32**
	**Fig. S27** GPC traces corresponding to polymerization in **Table 1**, Entry 6.	**S33**
	**Fig. S28** GPC traces corresponding to polymerization in **Table 1**, Entry 7.	**S33**
	**Fig. S29** GPC traces corresponding to polymerization in **Table 1**, Entry 8.	**S34**
	**Fig. S30** GPC traces corresponding to polymerization in **Table 3**, Entry 1.	**S34**
	**Fig. S31** GPC traces corresponding to polymerization in **Table 3**, Entry 2.	**S35**
	**Fig. S32** GPC traces corresponding to polymerization in **Table 3**, Entry 3.	**S35**
	**Fig. S33** GPC traces corresponding to polymerization in **Table 3**, Entry 4.	**S36**
	**Fig. S34** GPC traces corresponding to polymerization in **Table 3**, Entry 5.	**S36**
	**Fig. S35** GPC traces corresponding to polymerization in **Table 3**, Entry 6.	**S37**
	**Fig. S36** GPC trace corresponding to polymerization in **Table 3**, Entry 7.	**S37**
	**Fig. S37** GPC trace corresponding to polymerization in **Table 3**, Entry 8.	**S38**
	**Fig. S38** GPC trace corresponding to polymerization in **Table 3**, Entry 9.	**S38**
	**Fig. S39** GPC trace corresponding to polymerization in **Table 3**, Entry 10.	**S39**
	**Fig. S40** GPC trace corresponding to polymerization in **Table 3**, Entry 11.	**S39**
	**Fig. S41** GPC trace corresponding to polymerization in **Table 3**, Entry 12.	**S40**
	**Fig. S42** GPC traces corresponding to polymerization in **Table S1**, Entry 10.	**S40**
	**Fig. S43** GPC traces corresponding to polymerization in **Table S1**, Entry 11.	**S41**
	**Fig. S44** GPC traces corresponding to polymerization in **Table S1**, Entry 12.	**S41**
	**Fig. S45** GPC traces corresponding to polymerization in **Table 4**, Entry 2.	**S42**
	**Fig. S46** GPC traces corresponding to polymerization in **Table 4**, Entry 3.	**S42**
	**Fig. S47** GPC traces corresponding to polymerization in **Table 4**, Entry 4.	**S43**
	**Fig. S48** GPC traces corresponding to polymerization in **Table 4**, Entry 5.	**S43**
	**Fig. S49** GPC traces corresponding to polymerization in **Table 4**, Entry 7.	**S44**
	**Fig. S50** GPC traces corresponding to polymerization in **Table 4**, Entry 9.	**S44**
	**Fig. S51** GPC trace corresponding to polymerization in **Table 4**, Entry 10.	**S45**
	**Fig. S52** GPC trace corresponding to polymerization in **Table 4**, Entry 12.	**S45**
	**Fig. S53** GPC trace corresponding to polymerization in **Table 4**, Entry 14.	**S46**
	**Fig. S54** GPC traces corresponding to polymerization in **Table 3**, Entry 1.	**S46**
	**Fig. S55** GPC traces corresponding to polymerization in **Table 3**, Entry 2.	**S47**
	**Fig. S56** GPC traces corresponding to polymerization in **Table 3**, Entry 3.	**S47**
	**Fig. S57** GPC traces corresponding to polymerization in **Table 3**, Entry 4.	**S48**
	**Fig. S58** GPC traces corresponding to polymerization in **Table 3**, Entry 5.	**S48**
	**Fig. S59** GPC traces corresponding to polymerization in **Table S3**, Entry 1.	**S49**
	**Fig. S60** GPC traces corresponding to polymerization in **Table S3**, Entry 2.	**S49**
	**Fig. S61** GPC traces corresponding to polymerization in **Table S3**, Entry 3.	**S50**
	**Fig. S62** GPC traces corresponding to polymerization in **Table S3**, Entry 4.	**S50**
**7.**	**MALDI-TOF-MS Data**	**S51**
	**Fig. S63** MALDI-TOF mass spectrum of BO-*alt*-CPMA synthesized using $YCl_3THF_{3.5}$ (**Table 1**, Entry 7).	**S51**

Fig. S64 MALDI-TOF mass spectrum of BO-*alt*-CPMA synthesized using $YCl_3{\cdot}6H_2O$ (**Table 4**, Entry 1). **S52**
Fig. S65 MALDI-TOF mass spectrum of BO-*alt*-CPMA synthesized using $YCl_3THF_{3.5}$ (**Table 1**, Entry 5). **S53**
Fig. S66 MALDI-TOF mass spectrum of CHO-*alt*-CPMA synthesized using $YCl_3THF_{3.5}$ (**Table 1**, Entry 8). **S54**
8. **Differential Scanning Calorimetry Data** **S55**
Fig. S67 DSC traces of BO-*alt*-CPMA copolymers synthesized using $YCl_3THF_{3.5}$. **S55**
Fig. S68 Linear dependence of T_g on 1/M_n for BO-*alt*-CPMA. **S56**
Fig. S69 DSC traces of CHO-*alt*-CPMA copolymers synthesized using $YCl_3THF_{3.5}$. **S57**
9. **References** **S58**

1. General Considerations

All polymerization reactions were set up under anaerobic and anhydrous conditions in a Vacuum Atmospheres OMNI glovebox, unless stated otherwise. Protio and deuterated chloroform were purchased from commercial suppliers and used as received. 1-Butene oxide (BO) and cyclohexene oxide (CHO) were dried over CaH_2, degassed by three freeze-pump-thaw cycles and vacuum transferred prior to use. Carbic anhydride (CPMA) was recrystallized from hot 30:70 ethyl acetate/hexanes and dried under reduced pressure for 24 h prior to use. Phthalic anhydride (PA) and succinic anhydride (SA) were sublimed prior to use. Glutaric anhydride (GA) and cyclohexane anhydride (CHA) were purchased from commercial suppliers and used as received. Bis(triphenylphosphine)iminium chloride ([PPN]Cl) was recrystallized from CH_2Cl_2/hexanes and dried under reduced pressure for 24 h prior to use.

Methods. ^1H and $^{13}C\{^1H\}$ NMR spectra were recorded on a Varian 400-MR 2-Channel NMR spectrometer and referenced against residual protio solvent resonances. Polymer molecular weights and dispersities were determined using a SEC-MALS instrument equipped with an Agilent 1260 Infinity II HPLC System and autosampler, an Agilent PolyPore column (5 micron, 4.6 mm ID), a Wyatt DAWN HELEOS-II light scattering detector, and a Wyatt Optilab T-rEX refractive index detector. The columns were eluted with HPLC grade THF at 30 °C at a flow rate of 0.3 mL/min, and polymer samples were dissolved in this solvent and filtered through a 0.2 micron PTFE membrane before SEC-MALS analyses. dn/dc values were calculated from the RI signal by using

the 100% mass recovery method in the Astra software and a known sample concentration. When quality light scattering (LS) traces were unobtainable (Table 3, Entries 10-12; Table 4, Entries 10, 12, 14) GPC data was obtained using the RI signal and calibrating against a polystyrene standard curve. MALDI-TOF analyses were performed by the Mass Spectrometry Lab of the School of Chemical Sciences at the University of Illinois, Urbana Champaign on a Bruker Autoflex Speed LRF instrument. Differential scanning calorimetry (DSC) traces were recorded using a Perkin-Elmer DSC 8000 instrument and processed with a Pyris software. The DSC measurements were made at a heating rate of 10 °C/min and N_2 flow rate of 20 ml/min, and T_g values were obtained from the midpoint of the glass transition in the second heating curve. Finally, to demonstrate reproducibility of catalysis, the copolymerization of BO/CPMA was carried out in triplicate for both yttrium catalysts.

Metal Salt Synthesis. $YCl_3{\cdot}6H_2O$ was purchased from commercial suppliers and used as received. $YCl_3THF_{3.5}$ was prepared according to the reported literature procedure.[1]

Anhydrous YCl_3 was prepared using literature methods and a custom drying setup.[2,3] $YCl_3{\cdot}6H_2O$ (100 g) and 9 equivalents of NH_4Cl were ground together with a mortar and pestle. The solids were added to a custom glassware piece (Fig. S1), designed by Jorg Meyer. The solids were spread into the long tubing portion of the glassware, which was placed into a Linberg Blue tube furnace. The round bottom portion of the glassware was protruded out of the tube furnace, and connected to a secondary trap and a high vacuum line equipped with a custom three-stage diffusion pump and a primary trap. The connections leading to the custom glassware (which remained closed) were evacuated using a standard vacuum (~19 mTorr) for 12 hours. The Teflon-lined adapter to the custom glassware was then slowly opened as liquid nitrogen was poured into the primary trap. Dry ice and isopropanol were subsequently added to the secondary trap. Vacuum was switched to run through the diffusion pump, in which the pressure was kept below the mTorr limit of the pressure gauge. The glassware was heated to 100 °C for 24 hours, with traps kept full continuously. The temperature was then raised by 50 °C every hour until 230 °C is reached, which was held for 5-6 days to sublime off remaining

NH_4Cl into the round bottom portion outside the furnace. Once the YCl_3 was pale gray and appeared sandy, the setup was slowly cooled to room temperature. The adapter to the custom glassware was closed, which was then pumped into a glovebox. The custom glassware was scored and broken between the long tubing and round bottom portions. The YCl_3 was poured into a bottle to be stored in the glovebox. FT-IR spectroscopy of the YCl_3 confirmed no remaining water or NH_4Cl. The custom glassware is cleaned and then reformed by a glassblower.

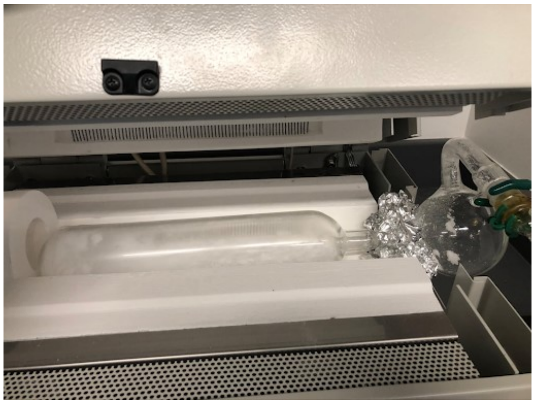

Fig. S1 Glassware for rare earth metal trichloride drying.

2. General Procedures for Polymerization

In a glovebox, $YCl_3THF_{3.5}$ (1 equiv), [PPN]Cl (1 equiv), anhydride, epoxide and a stir bar were charged into a vial equipped with a Teflon-lined cap. The vial was then taped with electrical tape, brought out of the glovebox, and placed inside a Chemglass high throughput tray or an oil bath that was preheated to 60 or 110 °C for at least 1 h. After the desired time, the vial was cooled to room temperature and the resulting mixture was dissolved in 1 mL of chloroform. Hexanes was then added in excess until the polymer started to precipitate out. The precipitate was allowed to settle, and the supernatant was pipette away. The isolated polymer was then dried under reduced pressure at 50 °C overnight. Polymerizations using the $YCl_3 \cdot 6H_2O$ catalyst were carried out using these same procedures but outside the glovebox. In addition, epoxides and anhydrides used for $YCl_3 \cdot 6H_2O$ reactions were purified and stored outside the glovebox. Conversions were calculated via analysis of crude ^1H NMR spectra in $CDCl_3$. ^1H NMR spectra of isolated

polymers for BO/CPMA,[4] BO/PA,[5] BO/SA,[6] CHO/CHA,[7] CHO/CPMA,[8] CHO/GA,[9] CHO/PA,[10] and CHO/SA[11] were in agreement with prior reports.

^{13}C NMR Spectroscopic Oligomer Studies

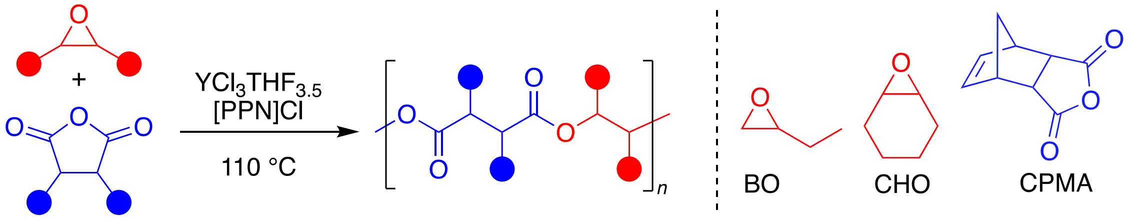

Scheme S1: Conditions used for ^{13}C spectroscopic NMR oligomer studies. Reactions performed in a J-young NMR tube. A 1:1:50:250 ratio of [$YCl_3THF_{3.5}$]:[[PPN]Cl]:[CPMA]:[epoxide] was used.

In a glovebox, $YCl_3THF_{3.5}$ (1 equiv), [PPN]Cl (1 equiv), CPMA (50 equiv), BO or CHO (250 equiv) and a stir bar were charged into a 2 dram vial. The mixture was stirred for 10 sec, and the resulting homogenous solution was transferred to a J-young NMR tube equipped with a sealed capillary tube containing a 15.7 mM solution of 1,4-bis(trimethylsilyl) benzene in C_6D_6. The sample was brought out of the glovebox, placed inside an oil bath, and heated at 110 °C for 5 min. An inverse-gated $^{13}C\{^1H\}$ NMR spectrum of the resulting solution was then recorded using 5 sec relaxation delay and 2000 scans.[12] The NMR tube was subsequently heated for a further 10 min (15 min total) and a $^{13}C\{^1H\}$ NMR spectrum was recorded again.

Table S1 Triplicate catalytic reactions for the copolymerization of BO and CPMA with yttrium trichloride catalysts and [PPN]Cl cocatalyst.[a]

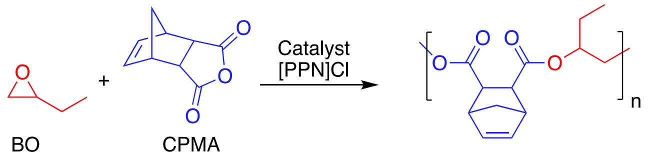

Entry	Catalyst	Temp.	Time	% yield[b]	TOF (h^{-1})[c]	% Ester[d]	% Epim.[e]	$^{Theor}M_n$ (kDa)[f]	$^{Exp}M_n$ (kDa)[g]	*Đ*[g]
1	YCl_3	60 °C	5 h	34	7	>99	<1	–	–	–
2	YCl_3	60 °C	5 h	85	17	>99	<1	–	–	–
3	YCl_3	60 °C	5 h	43	9	>99	<1	–	–	–
4	$YCl_3THF_{3.5}$	60 °C	5 h	95	19	>99	<1	5.3	6.4	1.02
5	$YCl_3THF_{3.5}$	60 °C	5 h	93	19	>99	<1	5.4	6.8	1.04
6	$YCl_3THF_{3.5}$	60 °C	5 h	91	18	>99	<1	5.3	5.7	1.05
7	$YCl_3THF_{3.5}$	110 °C	10 min	67	402	>99	<1	3.8	4.8	1.02
8	$YCl_3THF_{3.5}$	110 °C	10 min	67	402	>99	<1	3.8	4.8	1.09
9	$YCl_3THF_{3.5}$	110 °C	10 min	66	396	>99	<1	3.9	4.8	1.13
10	$YCl_3{\cdot}6H_2O$	110 °C	30 min	72	144	>99	<1	1.8[h]	1.7	1.05
11	$YCl_3{\cdot}6H_2O$	110 °C	30 min	77	154	>99	<1	1.8[h]	1.8	1.06
12	$YCl_3{\cdot}6H_2O$	110 °C	30 min	78	156	>99	<1	1.8[h]	1.9	1.07

[a] [BO]:[CPMA]:[Catalyst]:[[PPN]Cl] was 500:100:1:1. [b] Determined using ^{1}H NMR spectra of crude reaction mixtures, comparing the conversion of CPMA monomer to polymer. [c] Defined as mol CPMA consumed/(mol catalyst x h). [d] Determined using ^{1}H NMR spectra of crude reaction mixtures, comparing the polyether signal to a polyester signal. [e] Determined using ^{1}H NMR spectra of purified polymers as described previously: % Epim. = {2 x $A_{2.7\ ppm}$/($A_{6.0\text{-}6.5\ ppm}$)} x 100.[13] [f] Calculated for 4 initiating chlorides. [g] Identified by gel permeation chromatography (GPC), using a Wyatt MALS detector. [h] Calculated for 4 initiating chlorides and 6 water chain transfer agents.

3. Tabulated Polymerization Data

Table S2 Raw data for timepoint experiments (Fig. 3).[a]

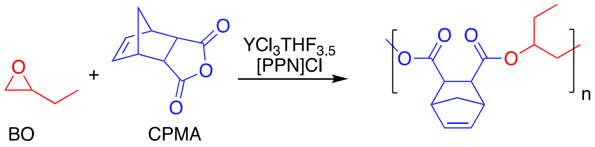

Entry	Time (min)	% yield[b]	$^{Theor}M_n$ (kDa)[c]	$^{Exp}M_n$ (kDa)[d]	$Đ$[d]
1	5	42	2.4	3.3	1.11
2	8	59	3.4	4.3	1.04
3	10	67	3.8	4.8	1.09
4	12	75	4.3	5.3	1.05
5	15	89	5.0	6.2	1.10

[a] [BO]:[CPMA]:[$YCl_3THF_{3.5}$]:[[PPN]Cl] was 500:100:1:1 at 110 °C . [b] Determined using ^1H NMR spectra of crude reaction mixtures, comparing the conversion of CPMA monomer to polymer. [c] Calculated for 4 initiating chlorides. [d] Identified by gel permeation chromatography (GPC), using a Wyatt MALS detector.

Table S3 Control reactions with $YCl_3THF_{3.5}$ under conditions of $YCl_3·6H_2O$ reactions.[a]

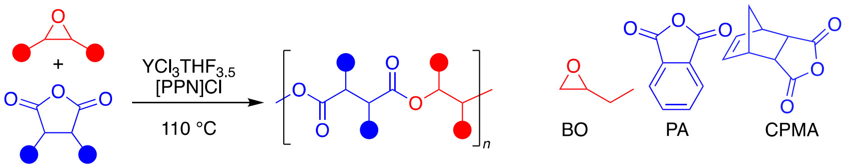

Entry	Catalyst	Monomers	Water Equiv.	Time (min)	% yield[b]	TOF (h^{-1})[c]	% Ester[d]	% Epimer.[e]	$^{Theor}M_n$ (kDa)[f]	$^{Exp}M_n$ (kDa)[g]	$Đ$[g]
1	$YCl_3THF_{3.5}$	BO/CPMA	None	30	67	134	>99	<1	4.0	4.5	1.15
2	$YCl_3THF_{3.5}$	BO/CPMA	6	30	67	134	>99	<1	1.6[h]	2.4	1.05
3	$YCl_3THF_{3.5}$	BO/PA	None	15	49	196	>99	-	2.8	2.9	1.01
4	$YCl_3THF_{3.5}$	BO/PA	6	15	89	356	>99	-	1.9[h]	1.4	1.01

[a] [Epoxide]:[Anhydride]:[Catalyst]:[[PPN]Cl] was 500:100:1:1 at 110 °C. Polymerizations performed outside the glove box using monomers stored on the lab bench. [b] Determined using ^1H NMR spectra of crude reaction mixtures, comparing the conversion of anhydride to polymer. [c] Defined as mol anhydride consumed/(mol catalyst x h). [d] Determined using ^1H NMR spectra of crude reaction mixtures, comparing the polyether signal to a polyester signal. [e] Determined using ^1H NMR spectra of purified polymers as described previously: % Epim. = {2 x $A_{2.7\ ppm}$/($A_{6.0\text{-}6.5\ ppm}$)} x 100.[13] [f] Calculated for 4 initiating chlorides. [g] Identified by gel permeation chromatography (GPC), using a Wyatt MALS detector. [h] Calculated for 4 initiating chlorides and 6 water chain transfer agents.

Table S4 Control reactions with [PPN]Cl as the only catalyst present.[a]

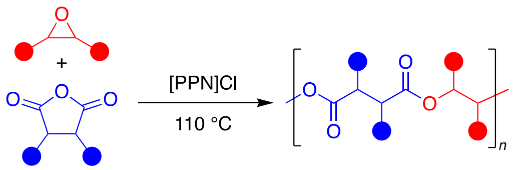

Entry	Monomers	Time (min)	% yield[b]
1	BO/CPMA	30	34
2	CHO/CPMA	20	13
3	BO/PA	15	95
4	CHO/PA	15	>99
5	BO/GA	50	69
6	CHO/GA	25	41
7	BO/SA	30	44
8	CHO/SA	17	38

[a] [Epoxide]:[Anhydride]:[[PPN]Cl] was 500:100:1 at 110 °C. Polymerizations performed under similar conditions to $YCl_3 \cdot 6H_2O$ reactions. [b] Determined using ^1H NMR spectra of crude reaction mixtures, comparing the conversion of anhydride to polymer.

Table S5 Prior examples of high molecular weight polyesters synthesized via ROCOP.

Entry	Monomers	Loading (mol %)[a]	Temp (° C)	TOF (h^{-1})[b]	$^{Theor}M_n$ (kDa)[c]	$^{Exp}M_n$ (kDa)	$Đ$	Ref
1	PO/PA	0.005	130	222	4123.9	200.0	1.25	14
2	PO/PA	0.013	80	1500	309.4	167.0	1.21	15
3	PO/PA	0.02	100	500	1030.9	118.5	1.91	16
4	PGE/MA	–	80	–	–	152.0	3.3	17
5	CBO/PA	0.006	50	70	462.4	223.5	1.11	18

[a] Based on the anhydride. [b] Defined as mol anhydride consumed/(mol catalyst x h). [c] Calculated as mol anhydride consumed x mol initiator^{-1} x MW repeat unit.

4. Discussion of TOF comparisons from Table 5

BO/CPMA

Despite $YCl_3THF_{3.5}$/[PPN]Cl having a lower TOF at 60 °C (19 vs. 92 h^{-1}), a much greater TOF (402 h^{-1}) is achieved at 110 °C while also keeping >99% polyester content and a dispersity of 1.08. This indicates the $YCl_3THF_{3.5}$/[PPN]Cl pair is temperature stable up to at least 110 °C, and that this temperature is optimal for our catalyst.

BO/PA

If we apply our catalyst with past literature conditions for BO/PA (Entry 5, Table 5), the TOF of $YCl_3{\cdot}6H_2O$/[PPN]Cl is not the largest. However, if we apply our optimized conditions (Entry 6, Table 5), which features the use of excess epoxide, $YCl_3{\cdot}6H_2O$/[PPN]Cl achieves the highest TOF while still maintaining >99% polyester linkages. This highlights how our catalytic system can use reaction condition that favor a faster rate without sacrificing control in % polyester and dispersity, unlike that of the past leading catalytic system.

BO/SA

The $YCl_3{\cdot}6H_2O$/[PPN]Cl pair achieves a much lower TOF (Entry 9, Table 5) compared to the past leading catalyst system (Entry 8, Table 5). This can be attributed to the fact that SA is not easily soluble in minimal amounts of epoxide, and especially toluene, as noted by the excess solids left over after 1.5 hours of reaction time for our catalytic run (Entry 9, Table S5). Therefore, it is hard to determine if the TOF can be attributed to catalyst efficiency, or also solubility issues.

CHO/SA

The $YCl_3{\cdot}6H_2O$/[PPN]Cl pair achieves the same TOF (Entry 11, Table 5) as the leading example in the literature (Entry 10, Table 5). One benefit of the $YCl_3{\cdot}6H_2O$/[PPN]Cl pair is that there is no polyether formation under the past literature conditions or our optimized conditions, making it the preferred catalyst choice as polyester control does not suffer as rate increases.

CHO/GA

The $YCl_3{\cdot}6H_2O$/[PPN]Cl pair achieves the lower TOF (Entry 13, Table 5) as the leading example in the literature (Entry 12, Table 5). Despite this lower TOF, running the

reaction at 110 °C with a higher [epoxide]:[anhydride] ratio raises the TOF to 321 h^{-1} while maintaining >99% polyester linkages (Entry 8, Table 5).

CHO/CHA

The $YCl_3 \cdot 6H_2O$/[PPN]Cl pair achieves a significantly higher TOF (Entry 15, Table 5) as the leading example in the literature (Entry 14, Table 5).

5. ^{1}H NMR Spectra of Isolated Polymers

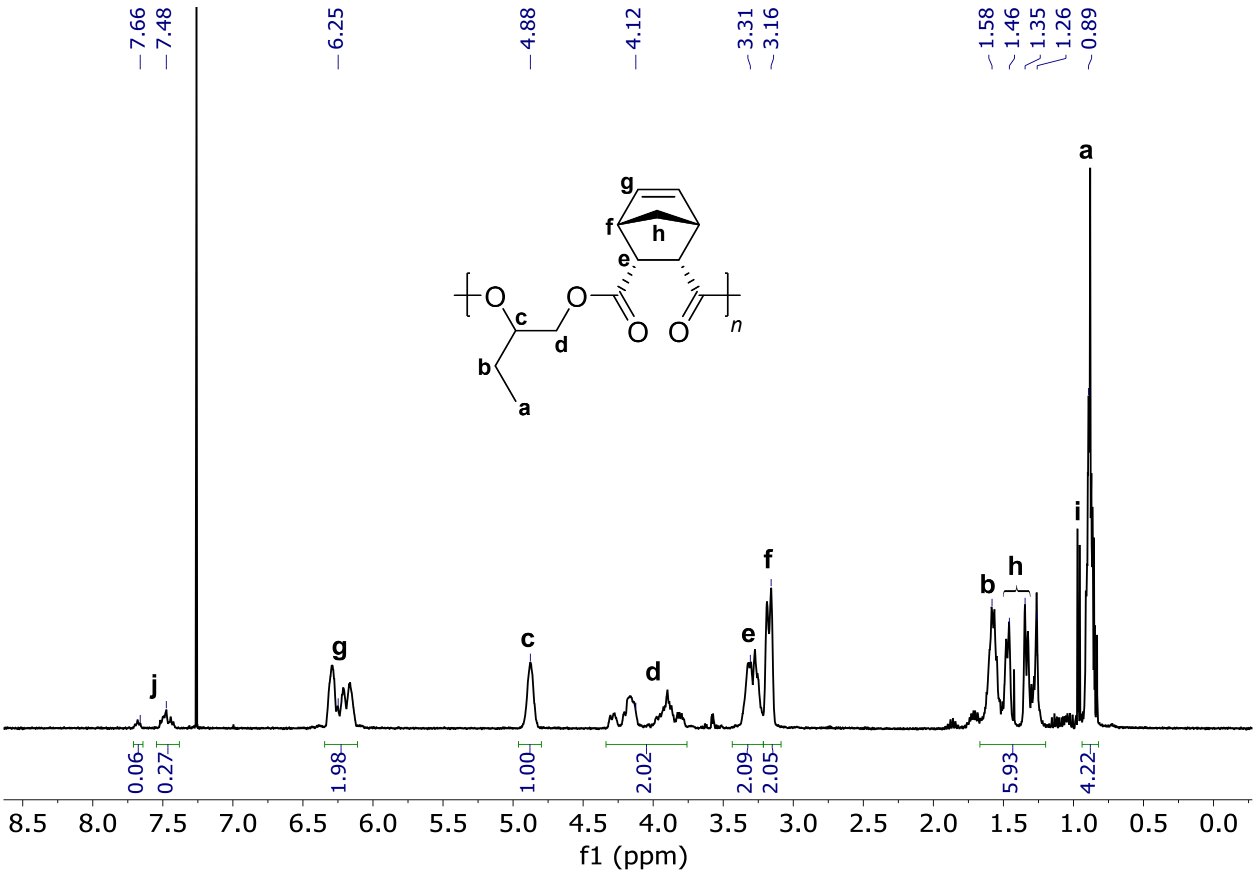

Fig. S2 ^{1}H NMR spectrum of BO-*alt*-CPMA in $CDCl_3$ (**Table S1**, Entry 7). Resonance **i** is assignable to hexanes; Resonance **j** is assignable to PPN phenyl groups.

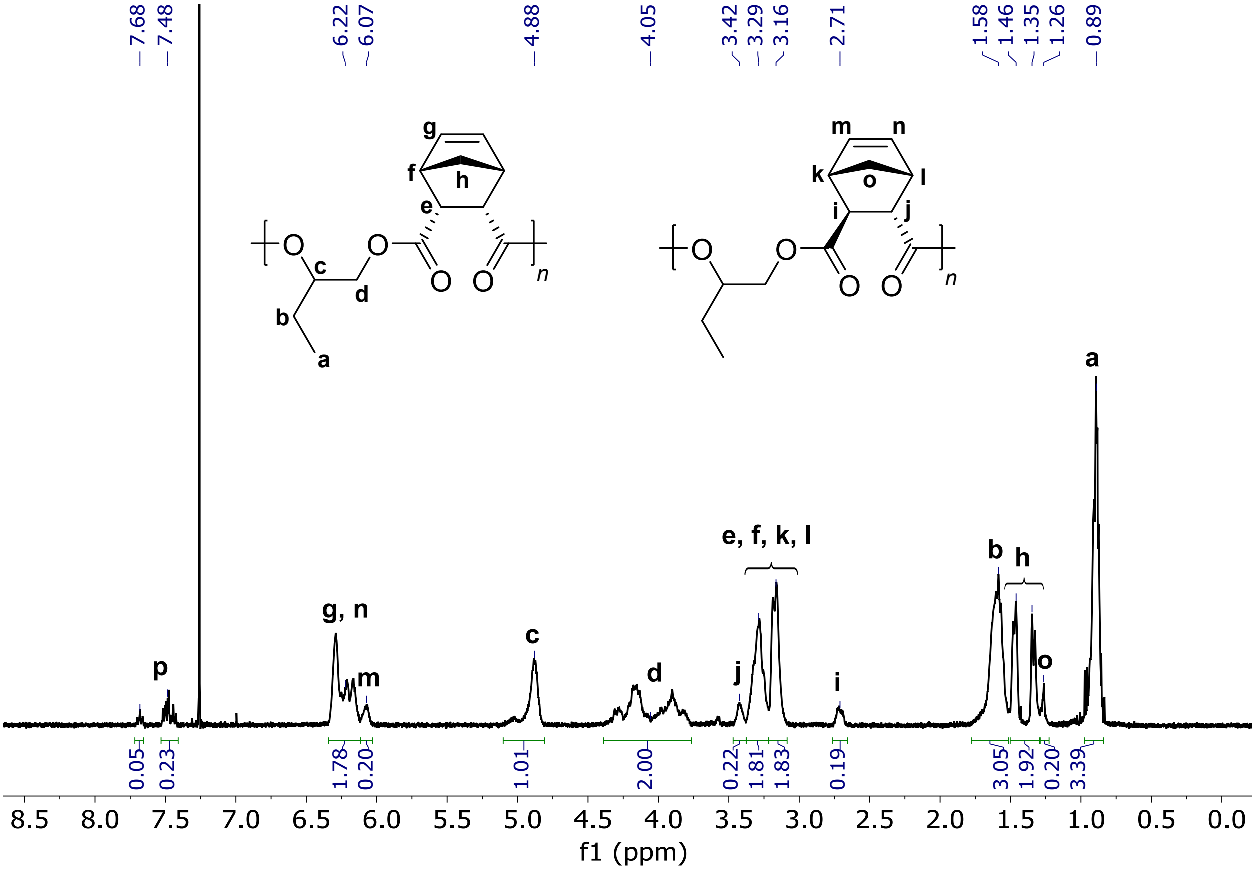

Fig. S3 ^{1}H NMR spectrum of BO-*alt*-CPMA in $CDCl_3$ (**Table 1**, Entry 5). Resonance **p** is assignable to PPN phenyl groups.

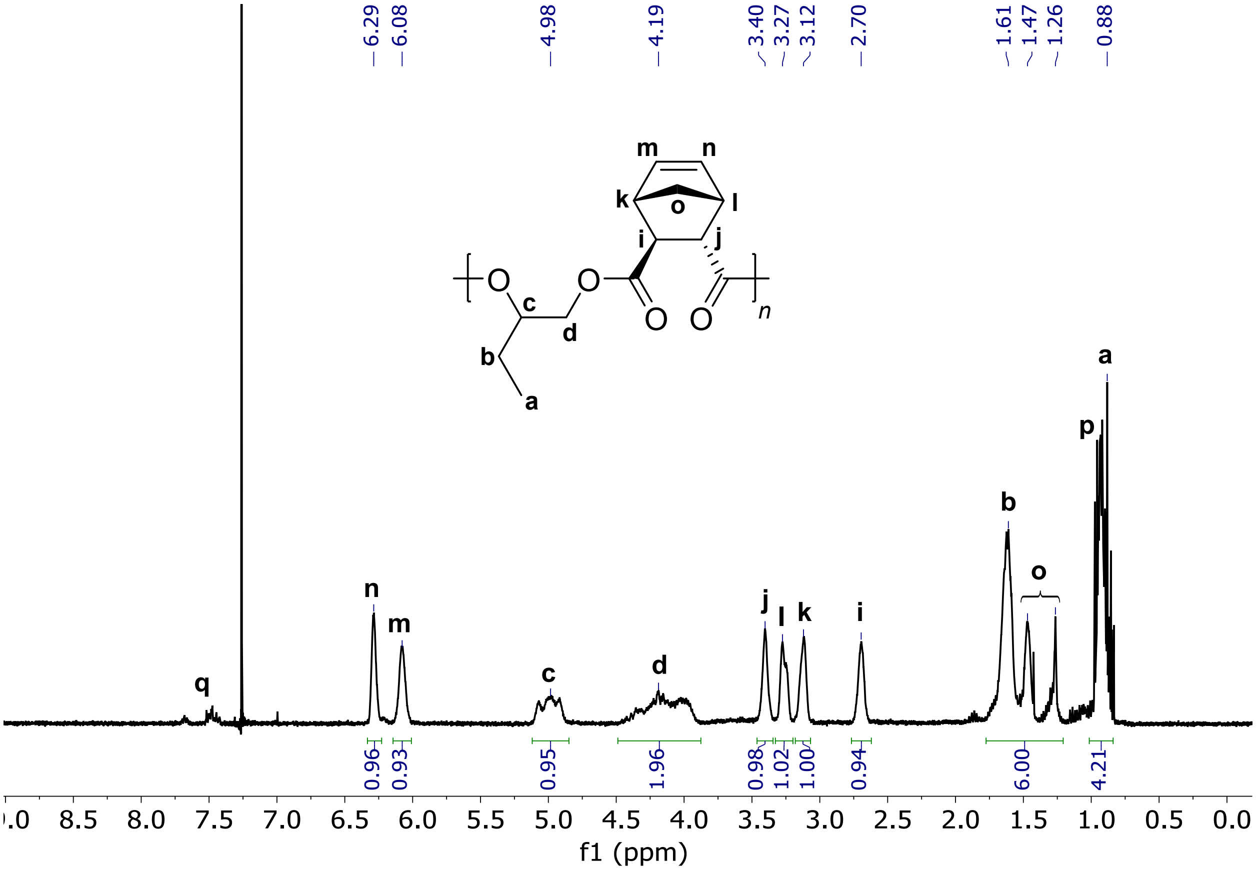

Fig. S4 ^{1}H NMR spectrum of BO-*alt*-CPMA in $CDCl_3$ (**Table 1**, Entry 6). Resonance **p** is assignable to hexanes; Resonances **q** is assignable to PPN phenyl groups.

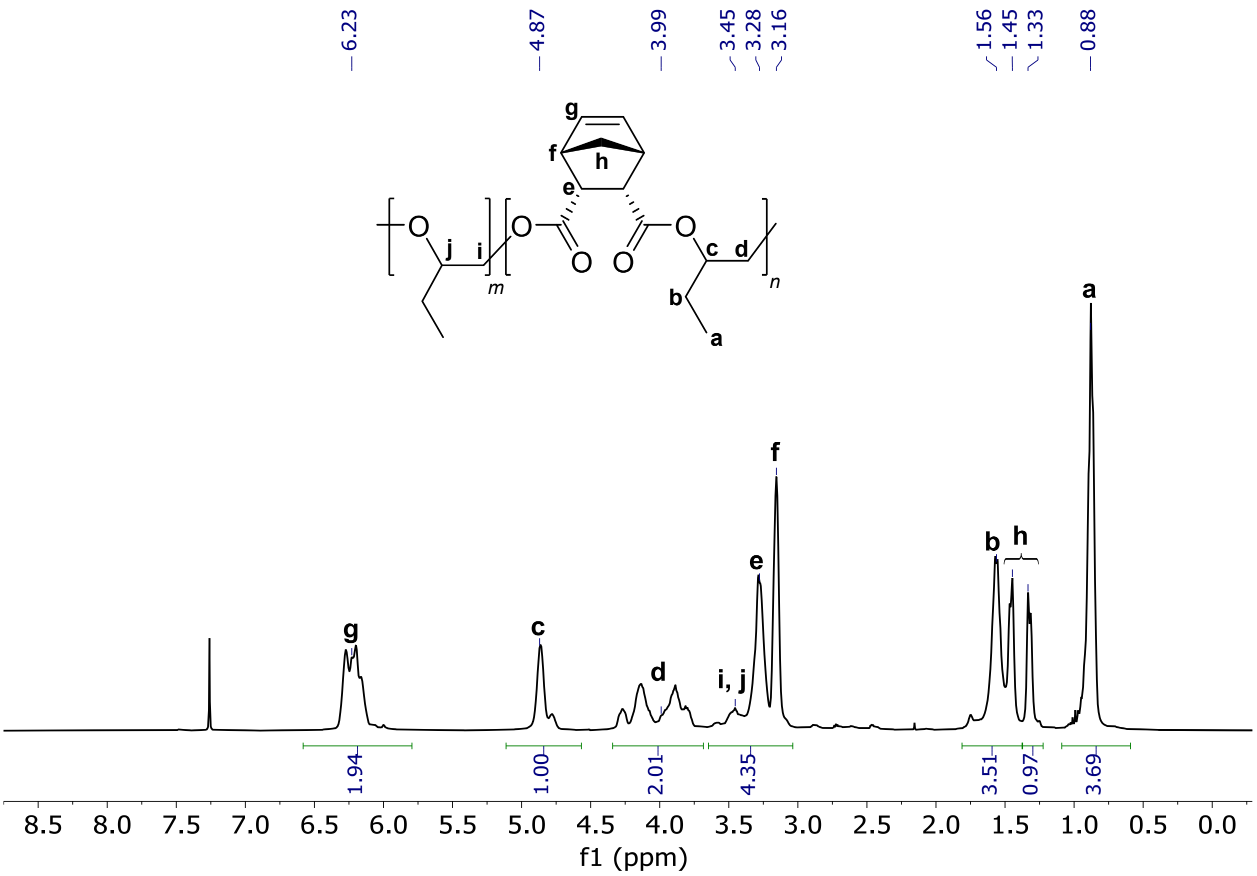

Fig. S5 ^{1}H NMR spectrum of high molecular weight BO-*alt*-CPMA in $CDCl_3$ (**Table 3**, Entry 3).

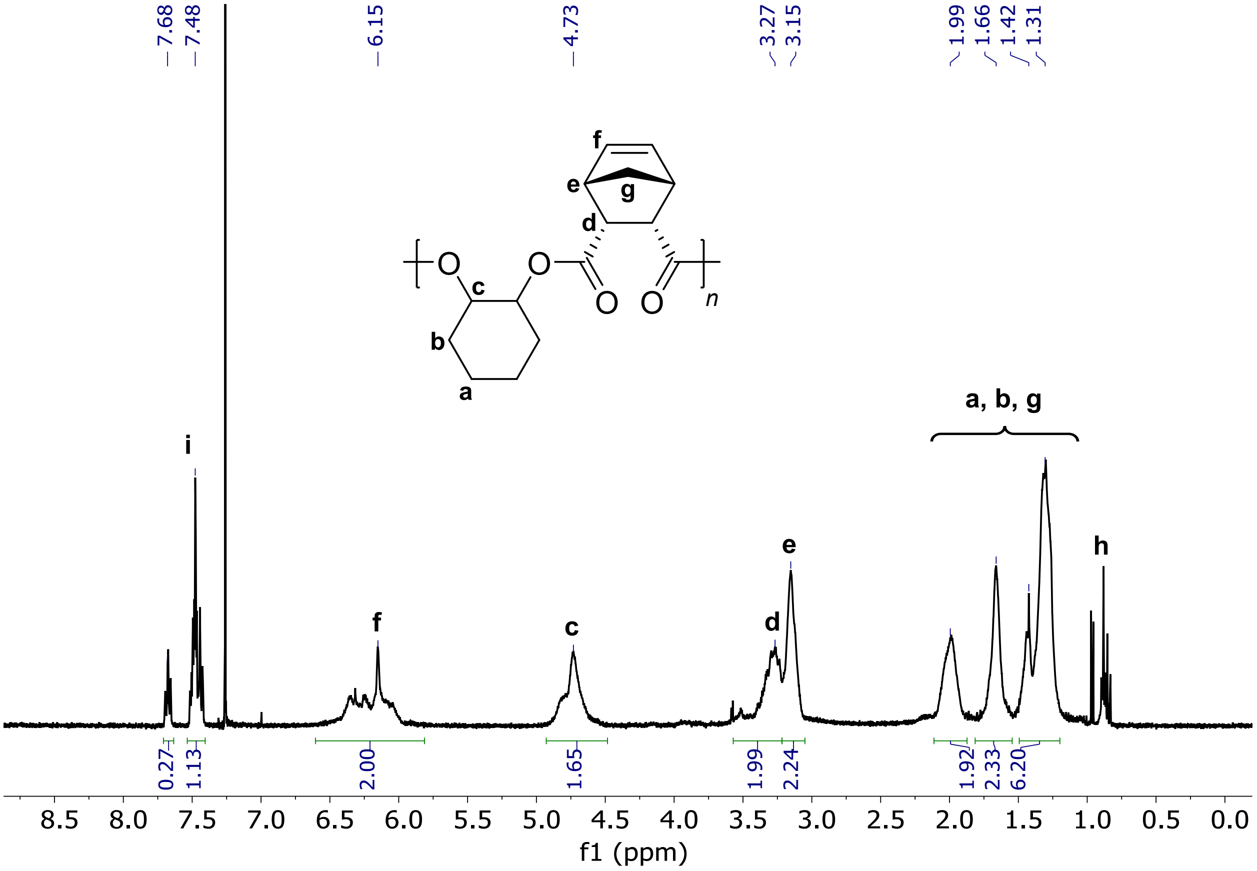

Fig. S6 ^{1}H NMR spectrum of CHO-*alt*-CPMA in $CDCl_3$ (**Table 1**, Entry 7). Resonance **h** is assignable to hexanes; Resonances **i** is assignable to PPN phenyl groups.

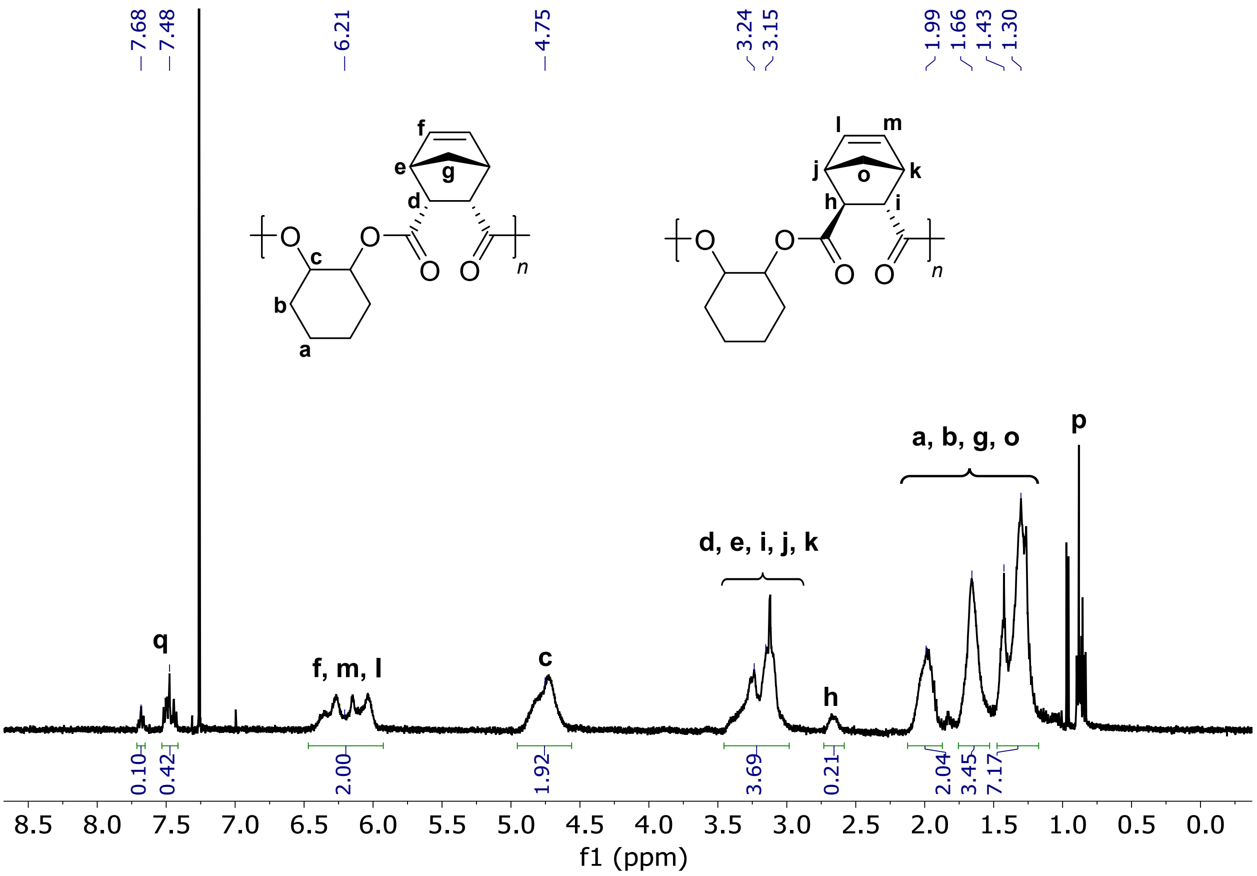

Fig. S7 ^{1}H NMR spectrum of CHO-*alt*-CPMA in $CDCl_3$ (**Table 1**, Entry 8). Resonance **p** is assignable to hexanes; Resonance **q** is assignable to PPN phenyl groups.

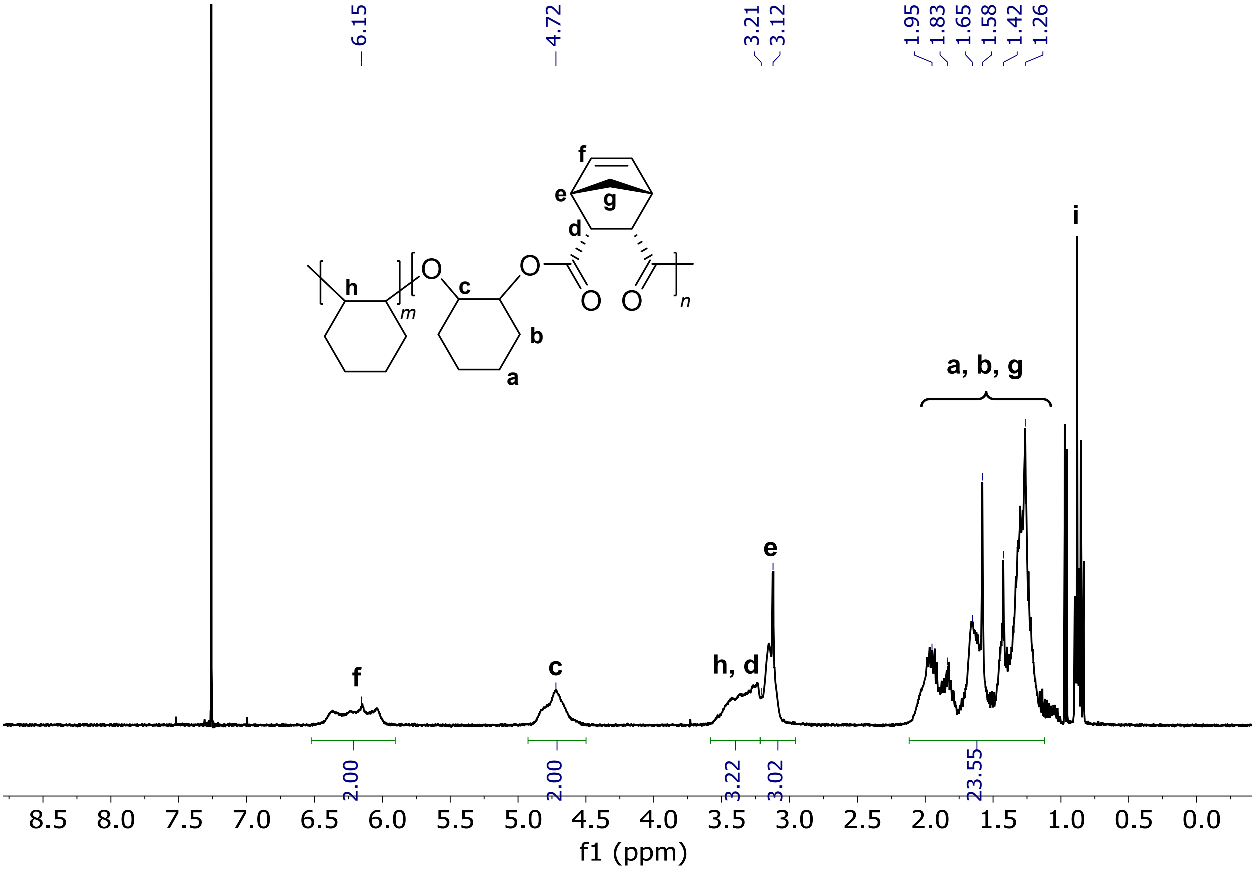

Fig. S8 ^{1}H NMR spectrum of high molecular weight CHO-*alt*-CPMA in $CDCl_3$ (**Table 3**, Entry 5). Resonance **i** is assignable to hexanes.

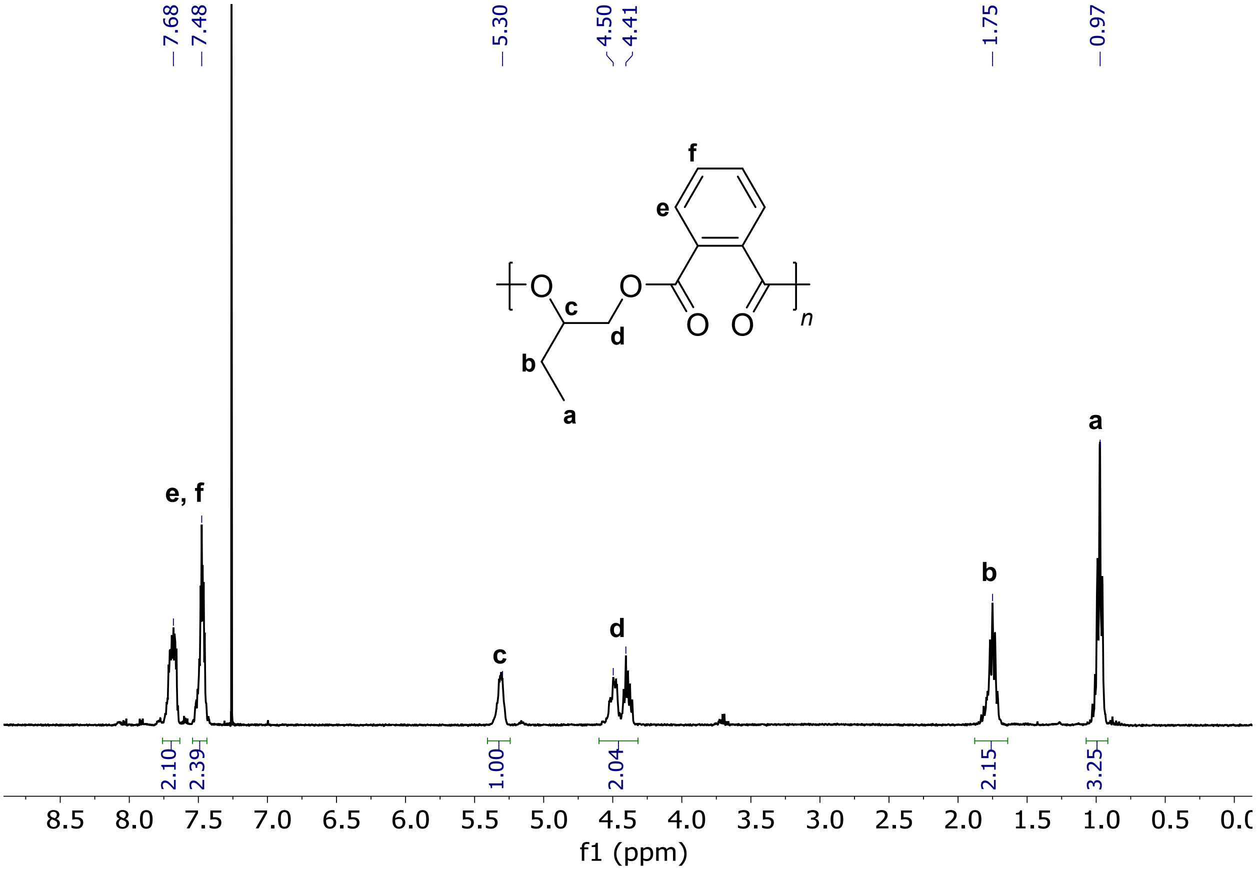

Fig. S9 ^{1}H NMR spectrum of BO-*alt*-PA in $CDCl_3$ (**Table 4**, Entry 1).

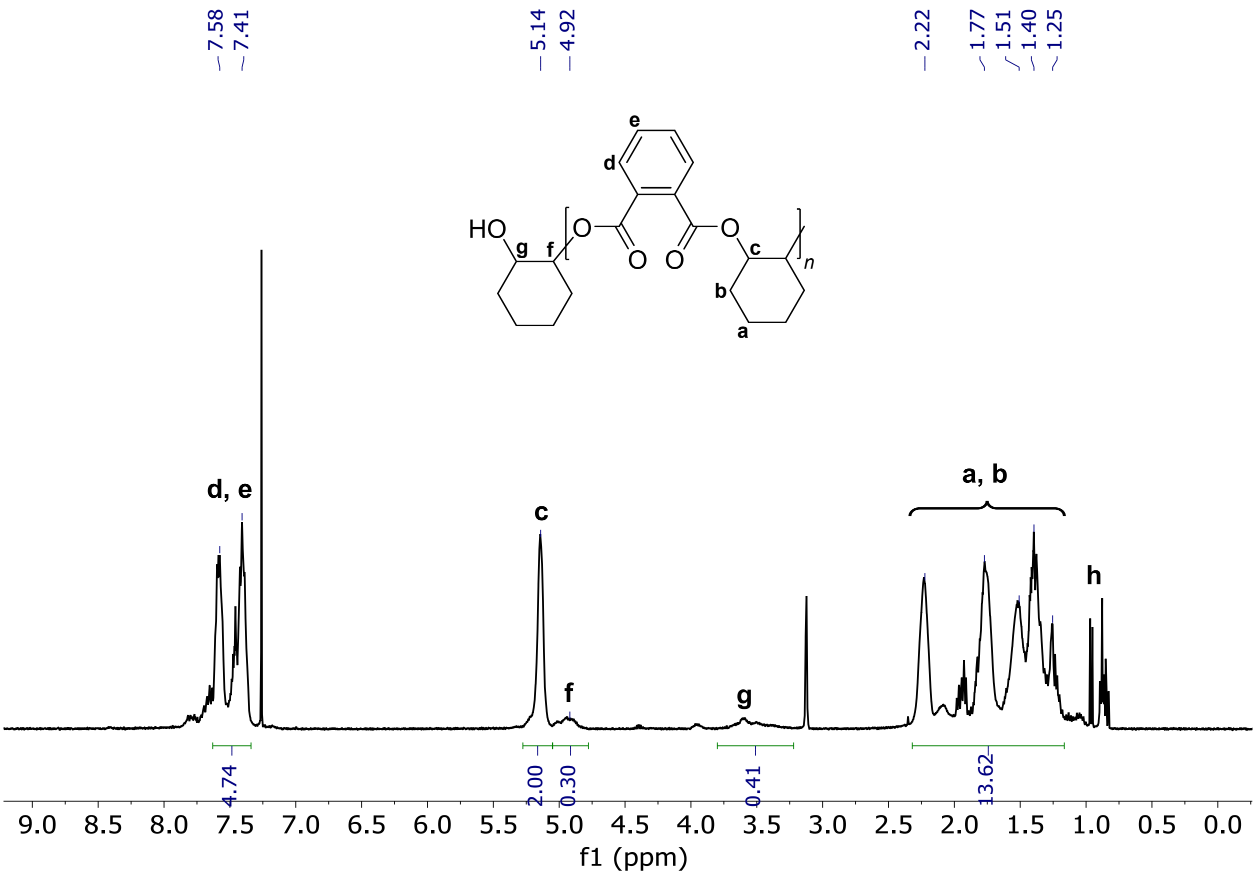

Fig. S10 ^{1}H NMR spectrum of CHO-*alt*-PA in $CDCl_3$ (**Table 4**, Entry 2). Resonance **h** is assignable to hexanes.

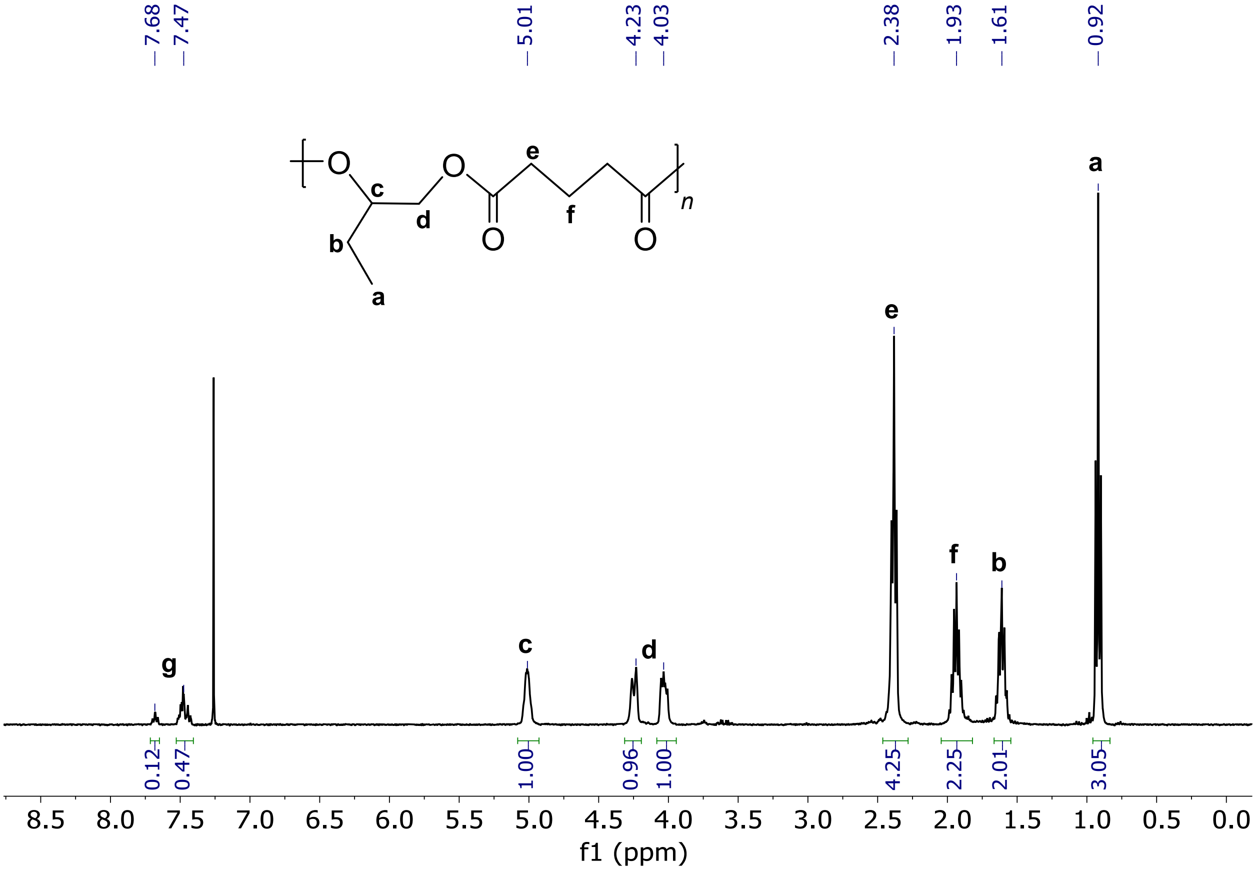

Fig. S11 ^{1}H NMR spectrum of BO-*alt*-GA in $CDCl_3$ (**Table 4**, Entry 3). Resonance **g** is assignable to PPN phenyl groups.

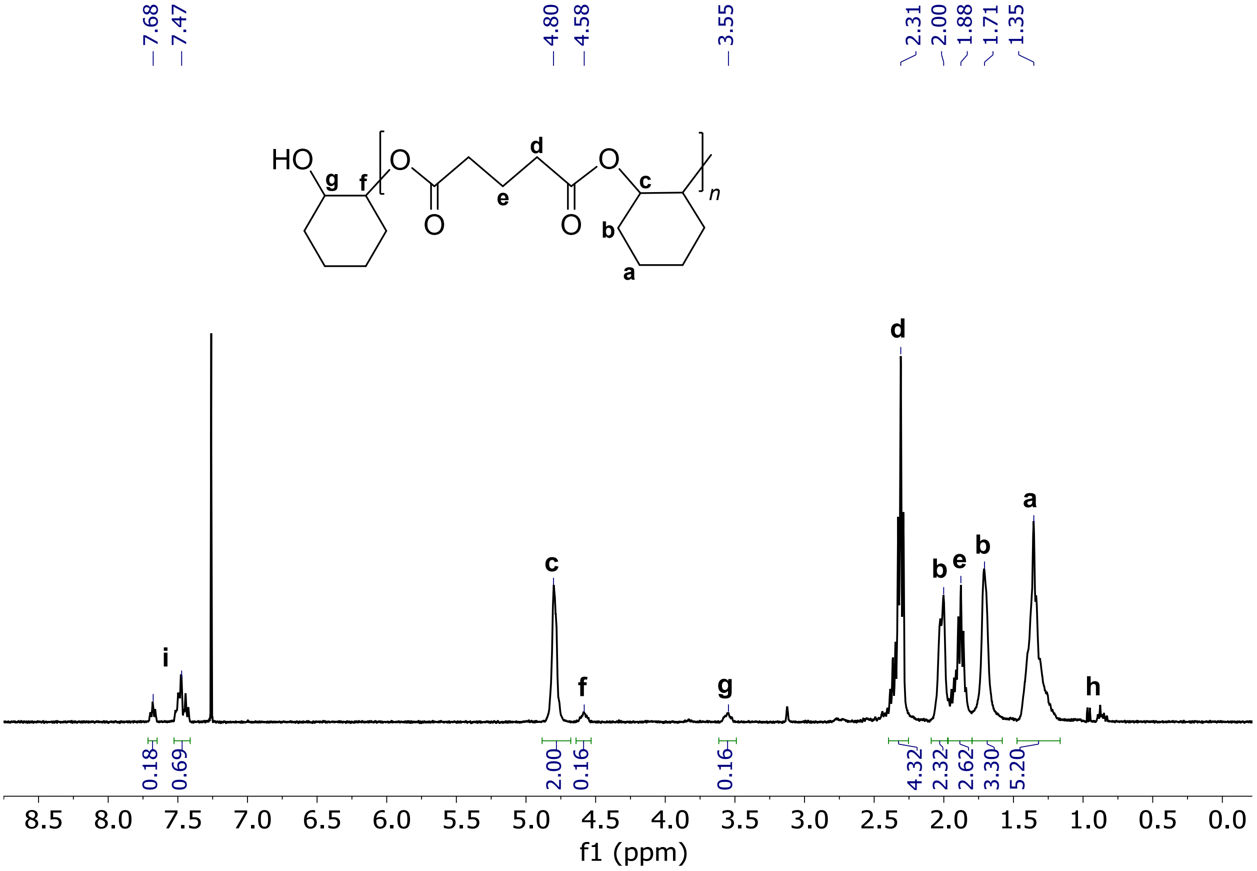

Fig. S12 ^{1}H NMR spectrum of CHO-*alt*-GA in $CDCl_3$ (**Table 4**, Entry 4). Resonance **h** is assignable to hexanes; Resonance **i** is assignable to PPN phenyl groups.

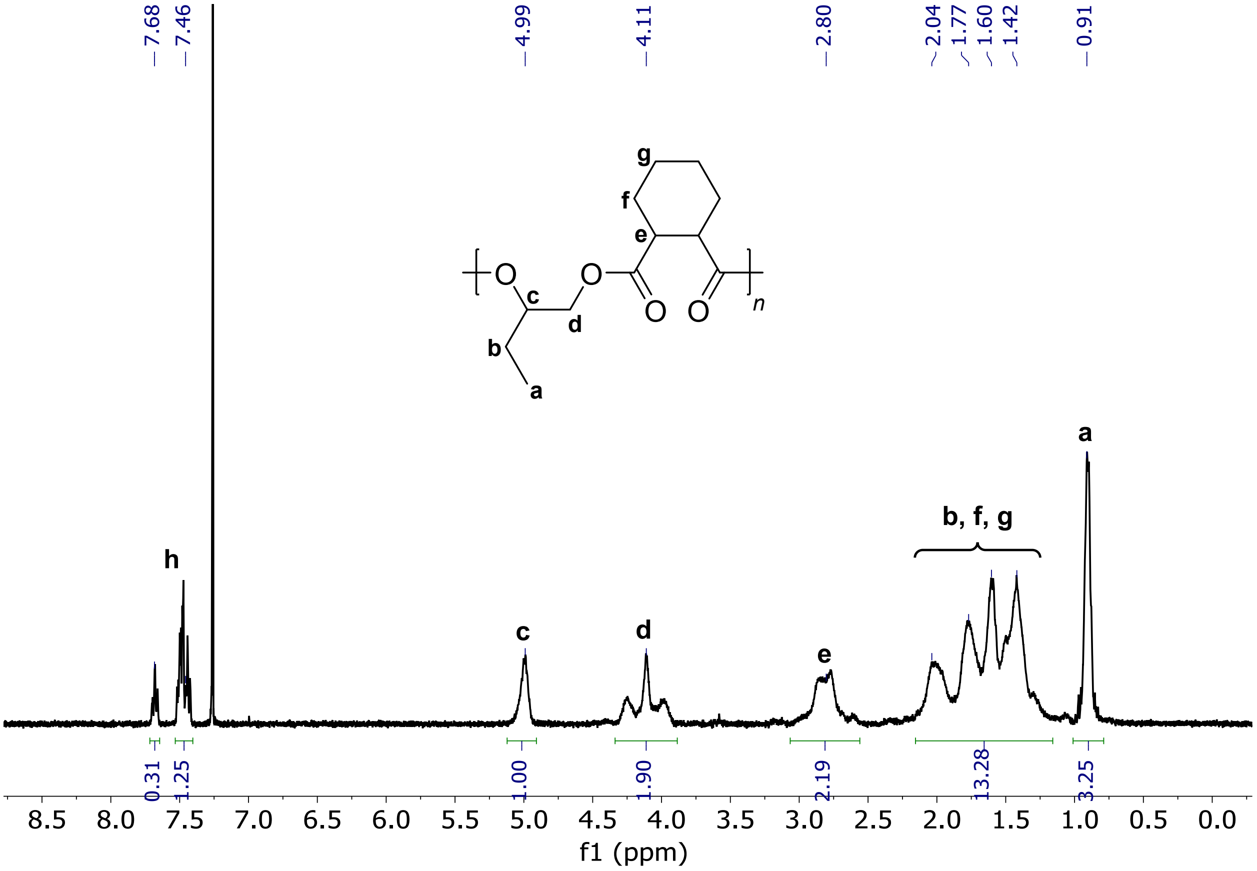

Fig. S13 ^{1}H NMR spectrum of BO-*alt*-CHA in $CDCl_3$ (**Table 4**, Entry 5). Resonance **h** is assignable to PPN phenyl groups.

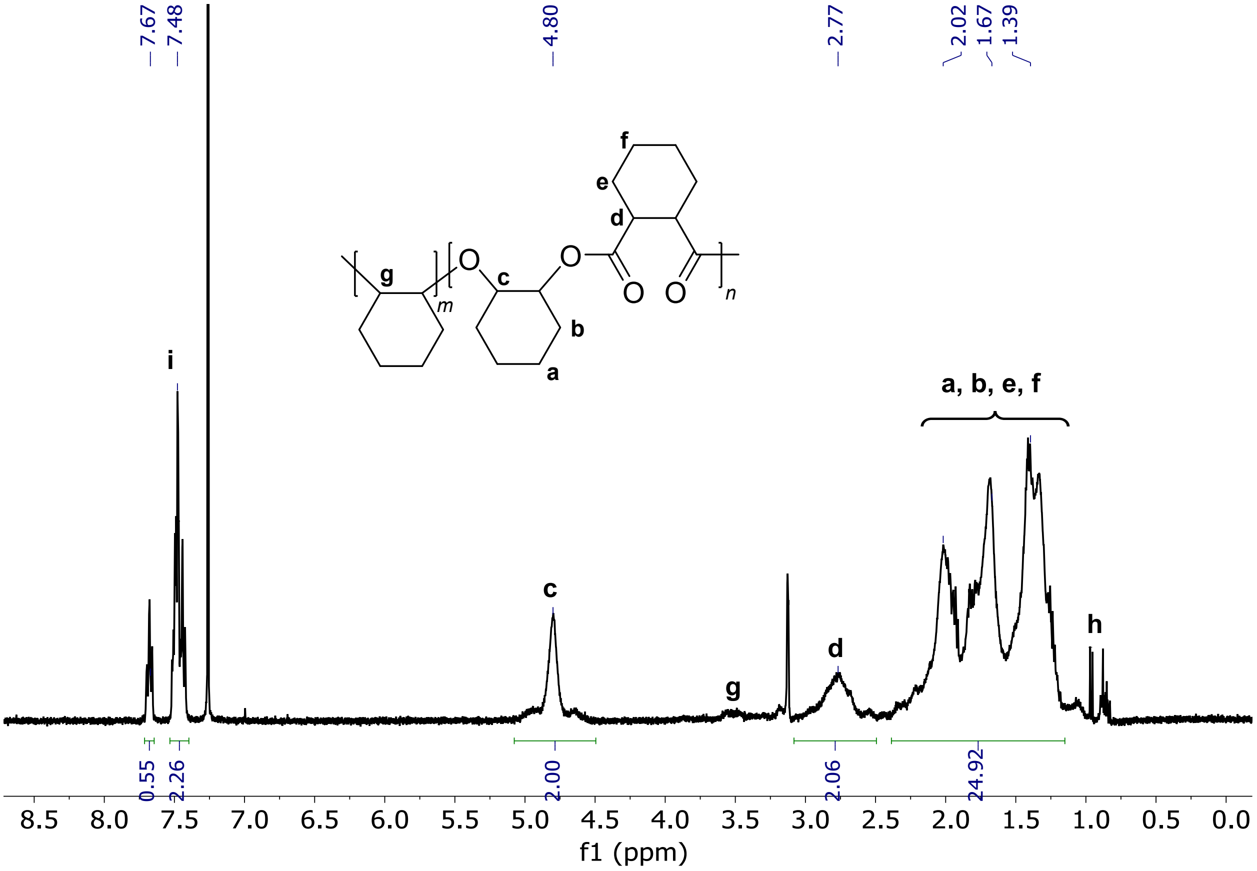

Fig. S14 ^{1}H NMR spectrum of CHO-*alt*-CHA in $CDCl_3$ (**Table 4**, Entry 6). Resonance **h** is assignable to hexanes; Resonance **i** is assignable to PPN phenyl groups.

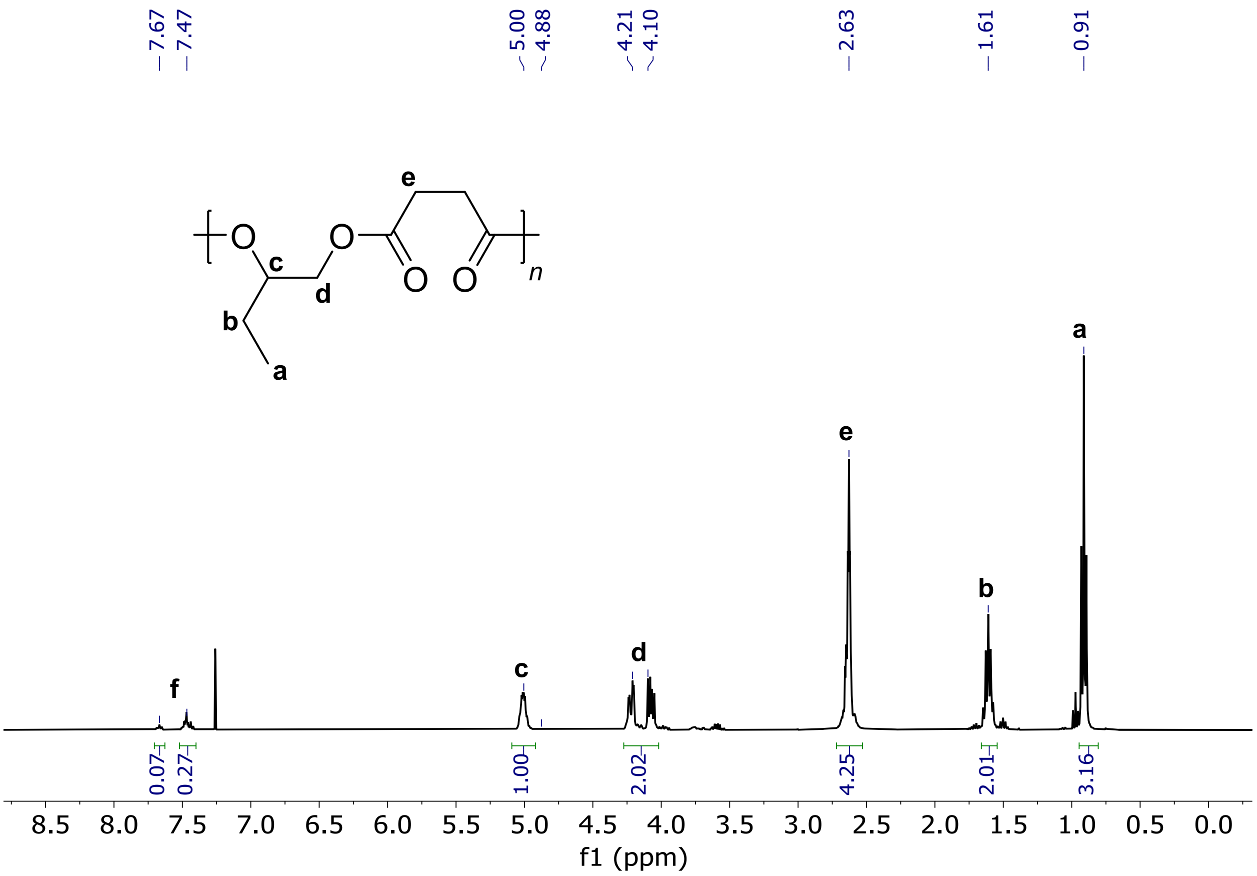

Fig. S15 ^{1}H NMR spectrum of BO-*alt*-SA in $CDCl_3$ (**Table 4**, Entry 7). Resonance **f** is assignable to PPN phenyl groups.

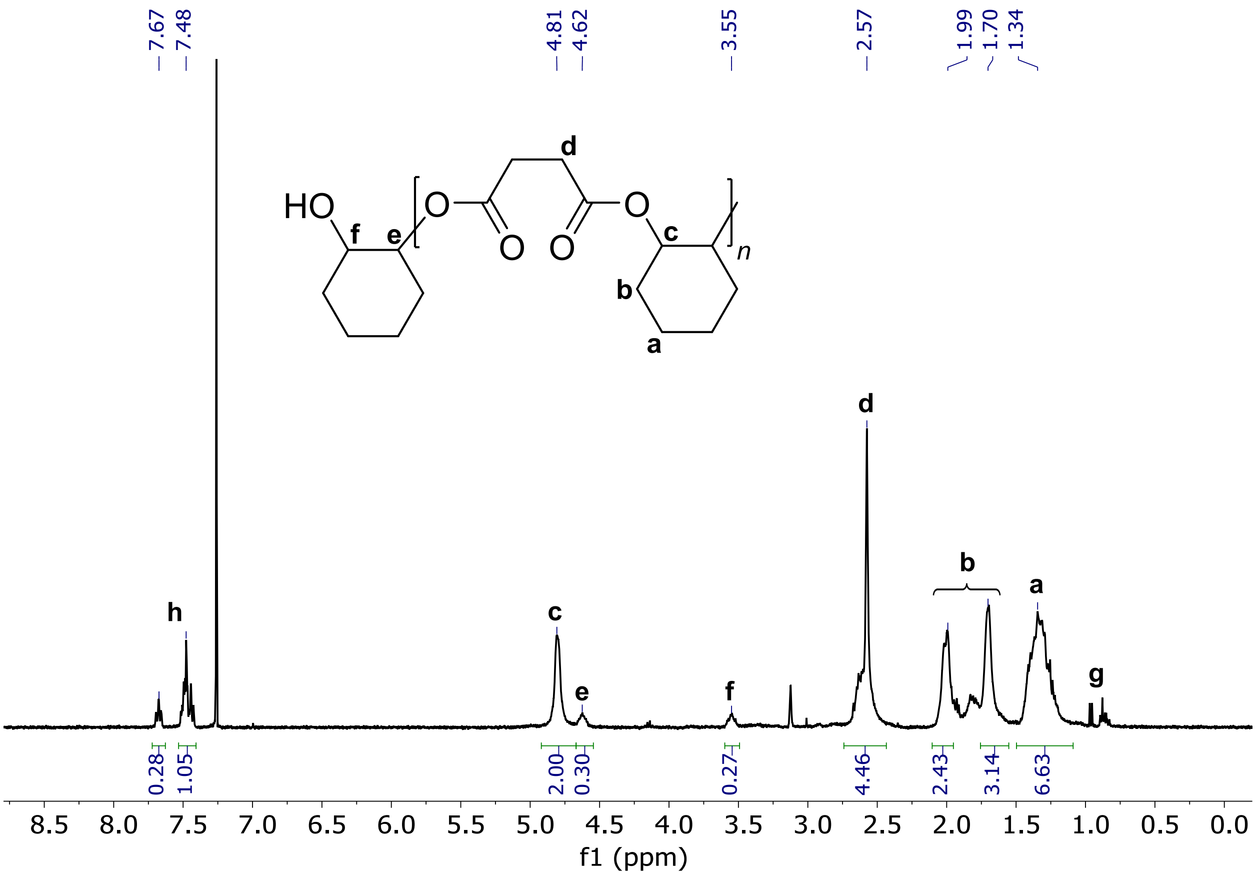

Fig. S16 ^{1}H NMR spectrum of CHO-*alt*-SA in $CDCl_3$ (**Table 4**, Entry 8). Resonance **g** is assignable to hexanes; Resonance **h** is assignable to PPN phenyl groups**.**

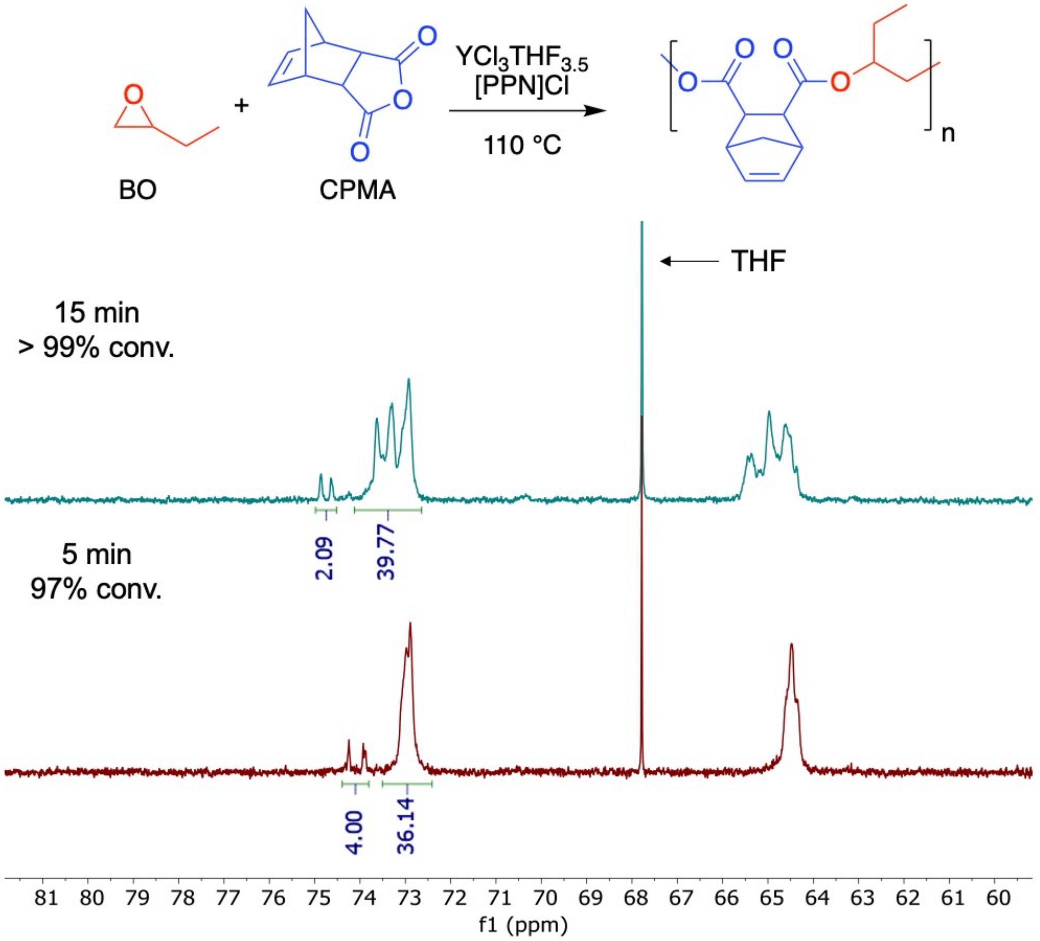

Fig. S17 In situ inverse-gated $^{13}C\{^1H\}$ NMR spectrum of BO-*alt*-CPMA copolymerization in C_6D_6 after 5 and 15 min of reaction time (1:1:50:250 ratio of $[YCl_3THF_{3.5}]$:[[PPN]Cl]:[CPMA]:[BO]). Resonances are integrated in reference to a 1,4-bis(trimethylsilyl) benzene internal standard.

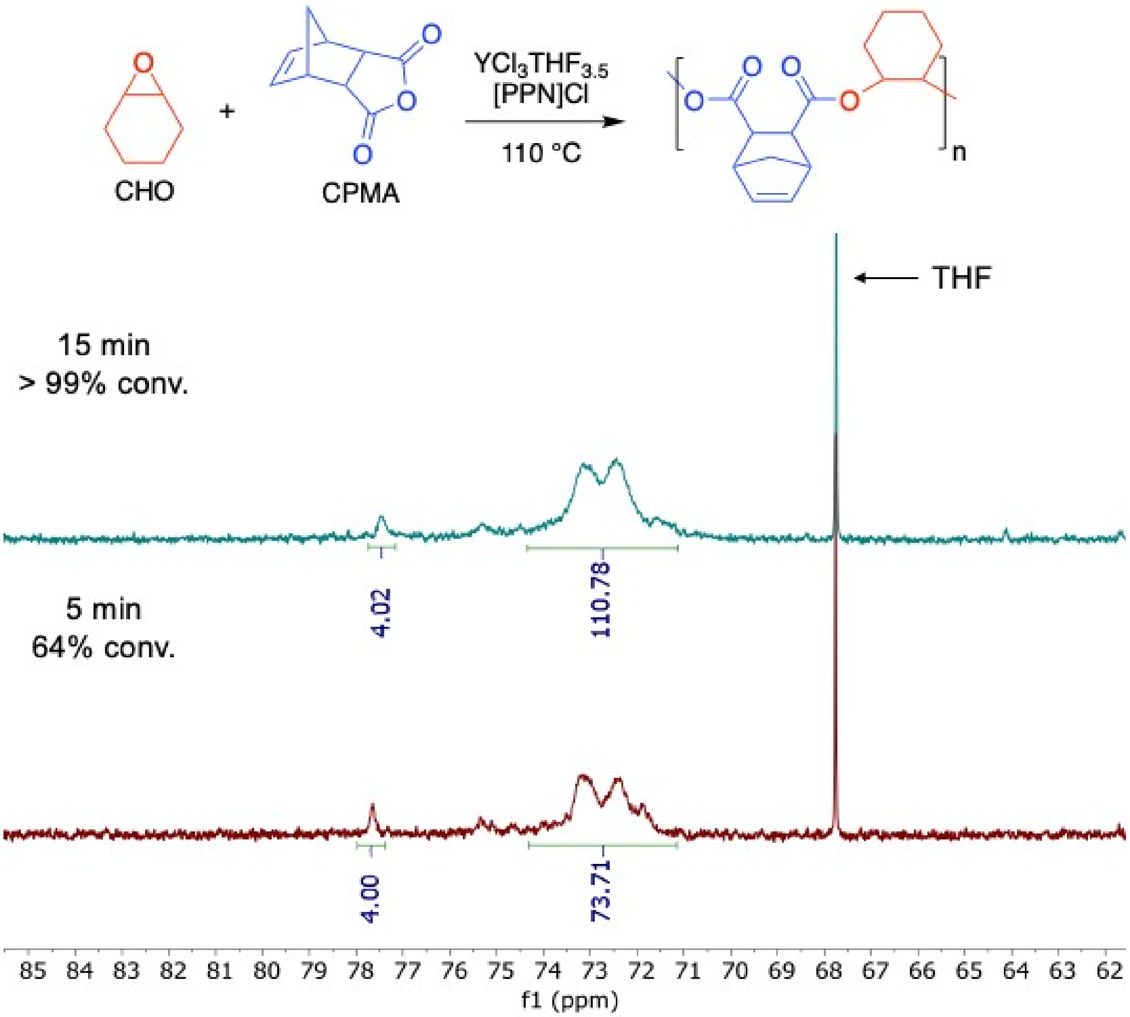

Fig. S18 In situ inverse-gated $^{13}C\{^{1}H\}$ NMR spectrum of CHO-*alt*-CPMA copolymerization in C_6D_6 after 5 and 15 min of reaction time (1:1:50:250 ratio of [$YCl_3THF_{3.5}$]:[[PPN]Cl]:[CPMA]:[CHO]). Resonances are integrated in reference to a 1,4-bis(trimethylsilyl) benzene internal standard.

6. GPC Data

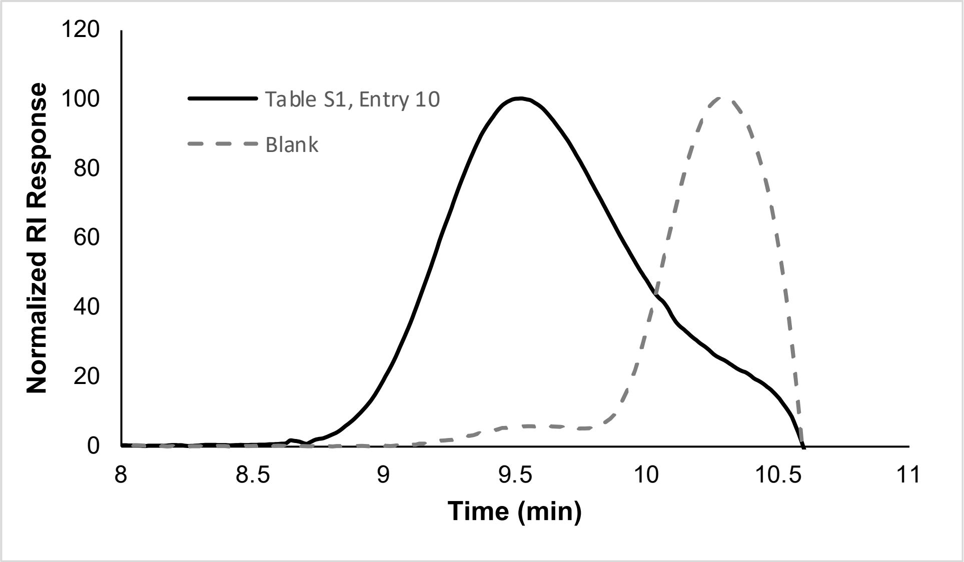

Fig. S19 GPC traces corresponding to polymerization in **Table S1**, Entry 10 (solid line) and blank of THF (dotted line).

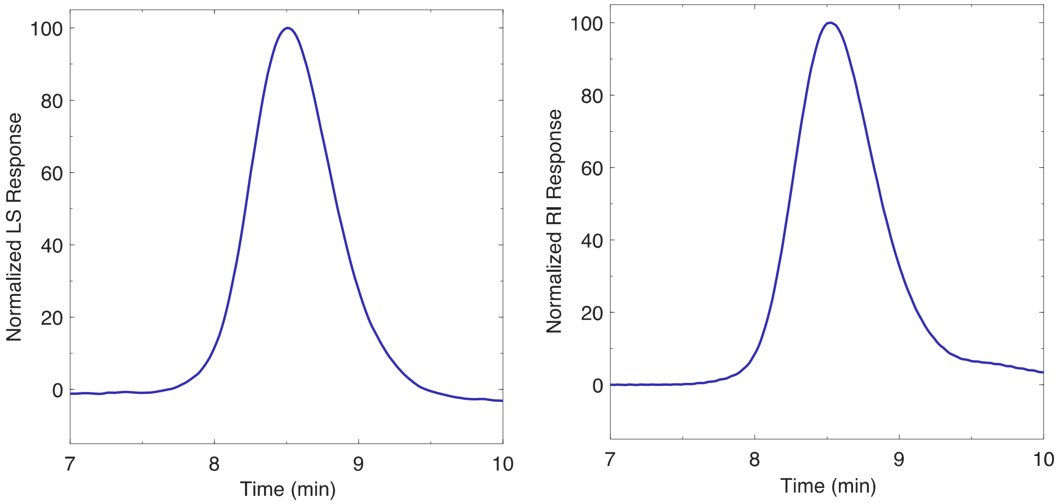

Fig. S20 GPC traces (MALS detector left, RI detector right) corresponding to polymerization in **Table S1**, Entry 4.

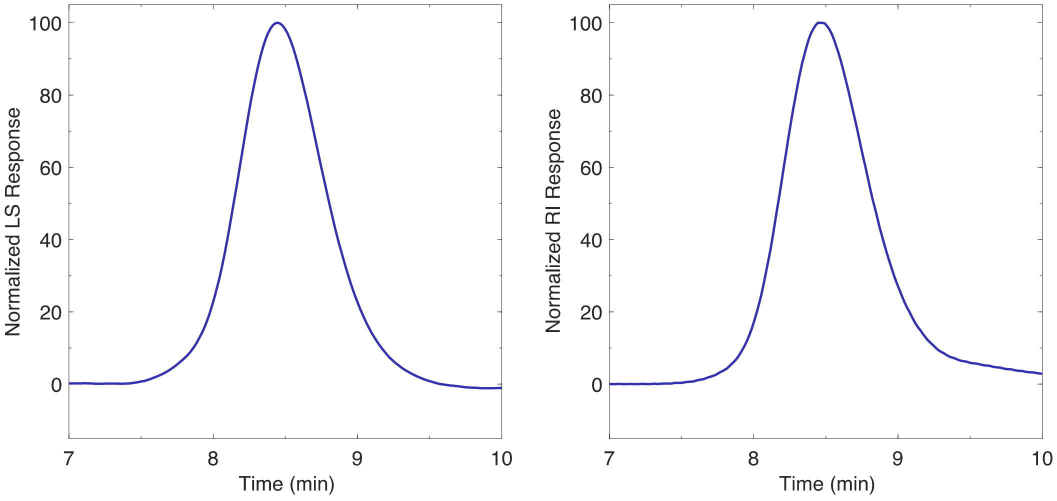

Fig. S21 GPC traces (MALS detector left, RI detector right) corresponding to polymerization in **Table S1**, Entry 5.

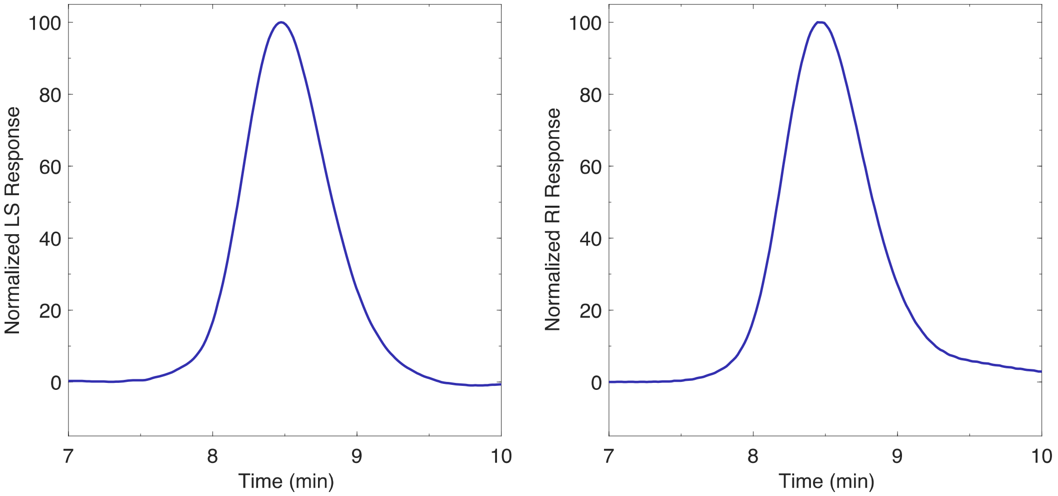

Fig. S22 GPC traces (MALS detector left, RI detector right) corresponding to polymerization in **Table S1**, Entry 6.

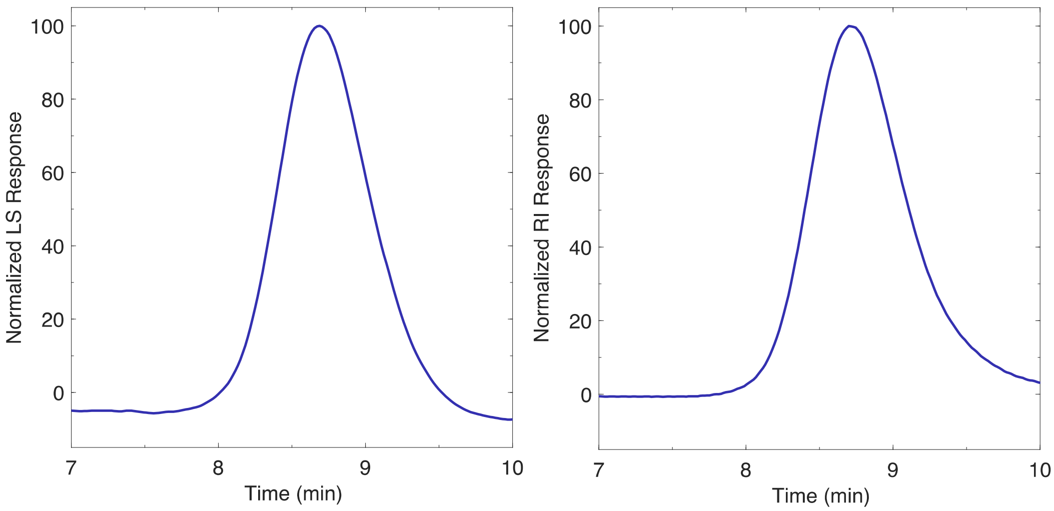

Fig. S23 GPC traces (MALS detector left, RI detector right) corresponding to polymerization in **Table S1**, Entry 7.

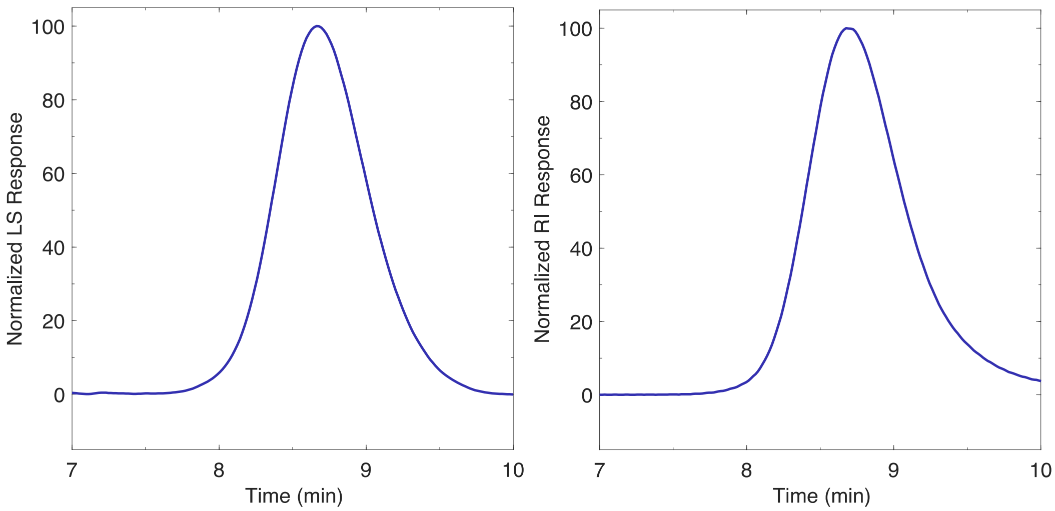

Fig. S24 GPC traces (MALS detector left, RI detector right) corresponding to polymerization in **Table S1**, Entry 8.

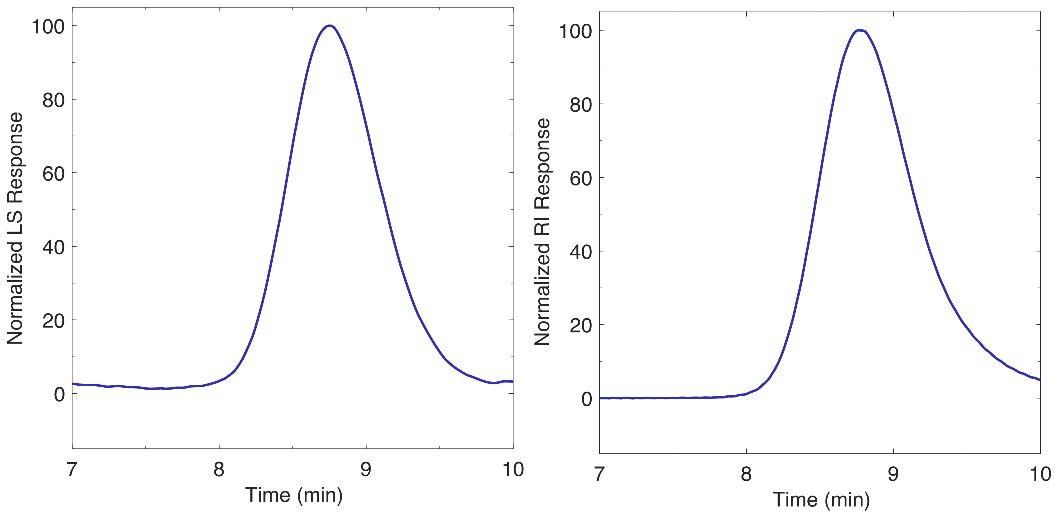

Fig. S25 GPC traces (MALS detector left, RI detector right) corresponding to polymerization in **Table S1**, Entry 9.

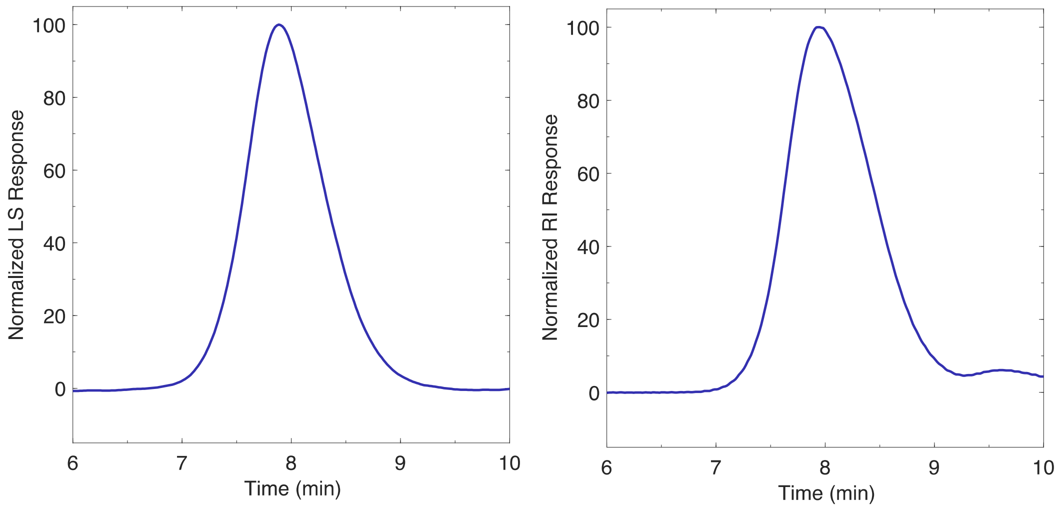

Fig. S26 GPC traces (MALS detector left, RI detector right) corresponding to polymerization in **Table 1**, Entry 5.

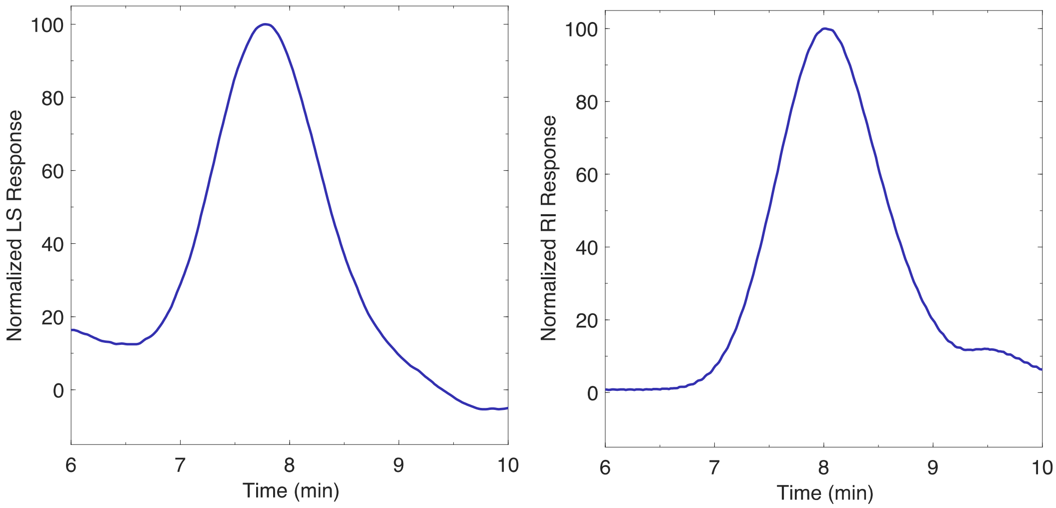

Fig. S27 GPC traces (MALS detector left, RI detector right) corresponding to polymerization in **Table 1**, Entry 6.

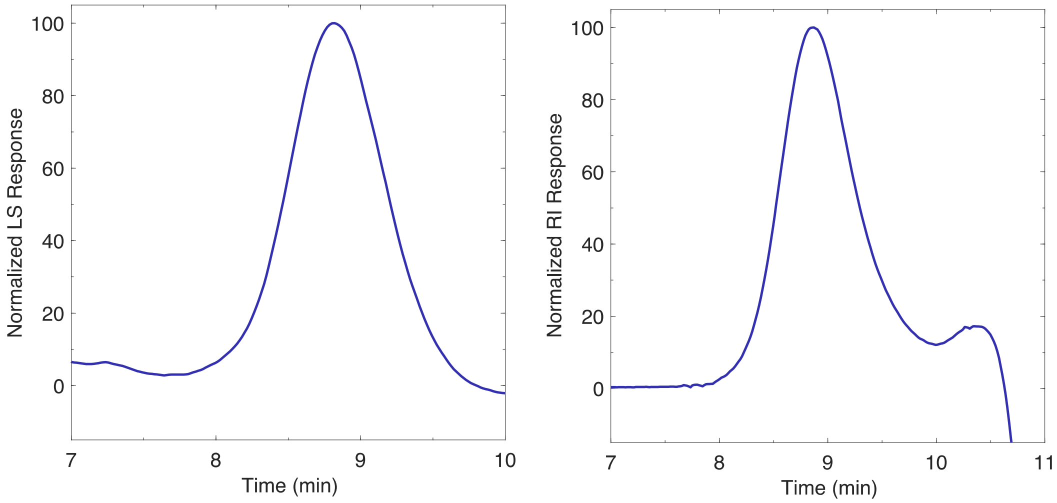

Fig. S28 GPC traces (MALS detector left, RI detector right) corresponding to polymerization in **Table 1**, Entry 7.

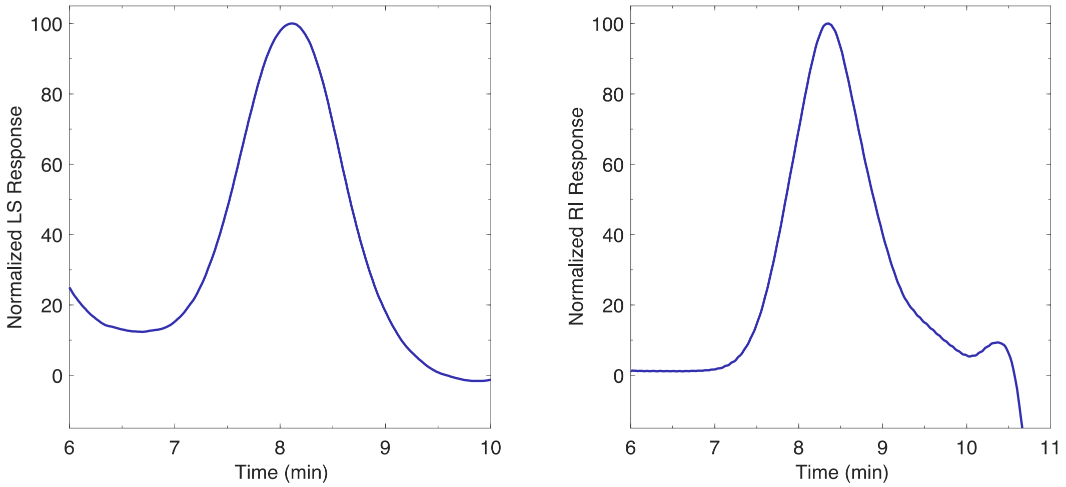

Fig. S29 GPC traces (MALS detector left, RI detector right) corresponding to polymerization in **Table 1**, Entry 8. Peak onset at 6 min on the LS trace is due to column shedding.

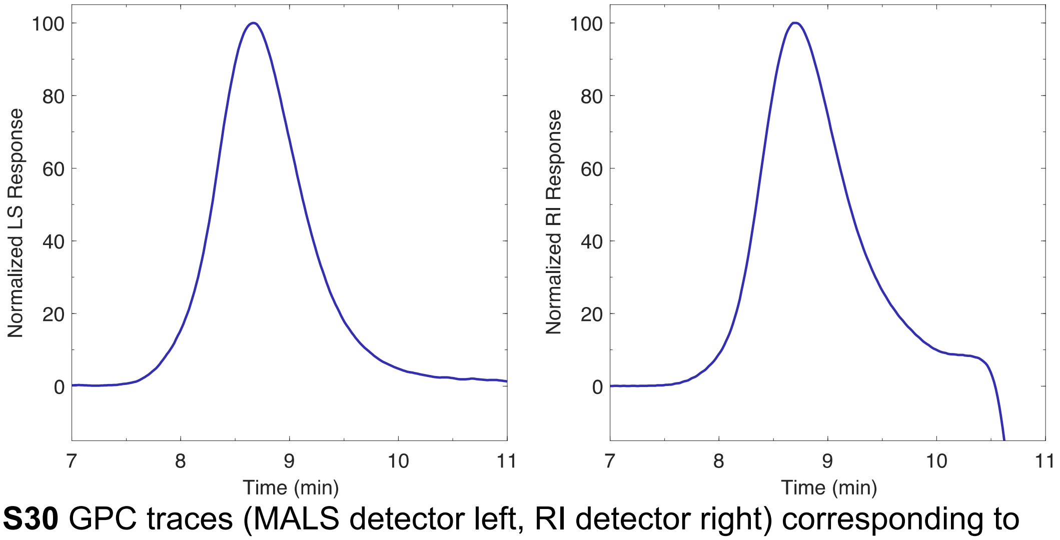

Fig. S30 GPC traces (MALS detector left, RI detector right) corresponding to polymerization in **Table 3**, Entry 1.

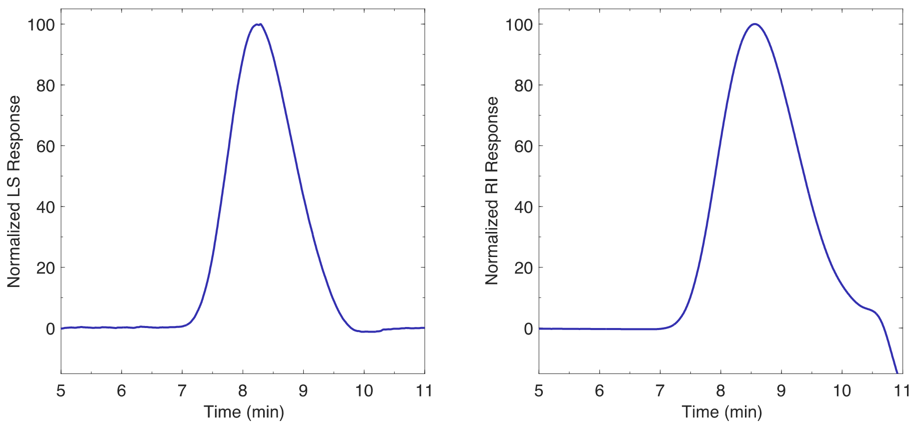

Fig. S31 GPC traces (MALS detector left, RI detector right) corresponding to polymerization in **Table 3**, Entry 2.

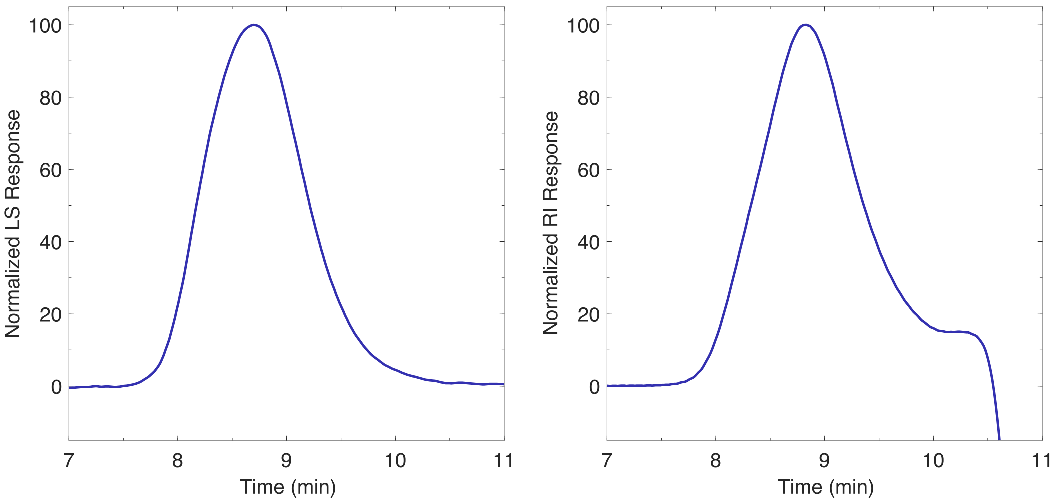

Fig. S32 GPC traces (MALS detector left, RI detector right) corresponding to polymerization in **Table 3**, Entry 3.

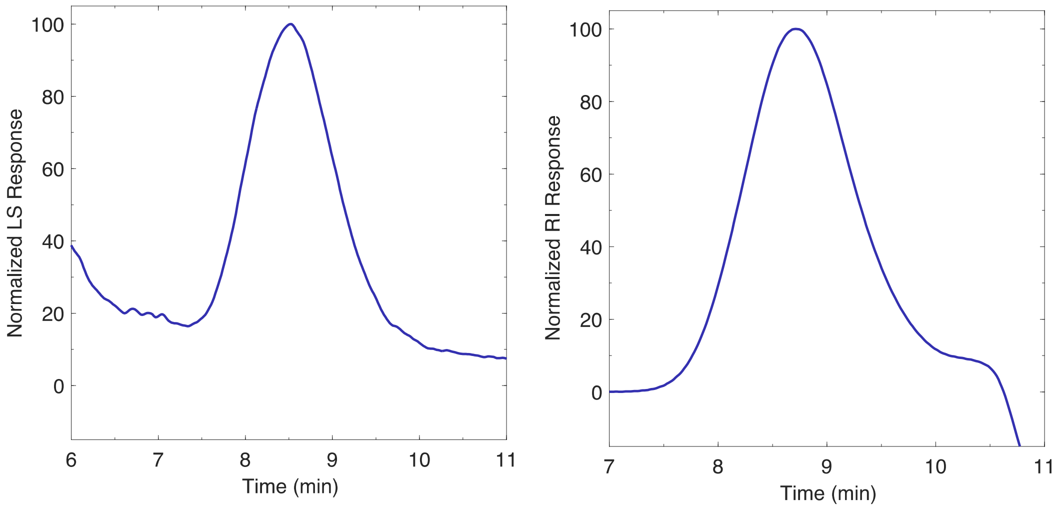

Fig. S33 GPC traces (MALS detector left, RI detector right) corresponding to polymerization in **Table 3**, Entry 4. Peak onset at 6 min on the LS trace is due to column shedding.

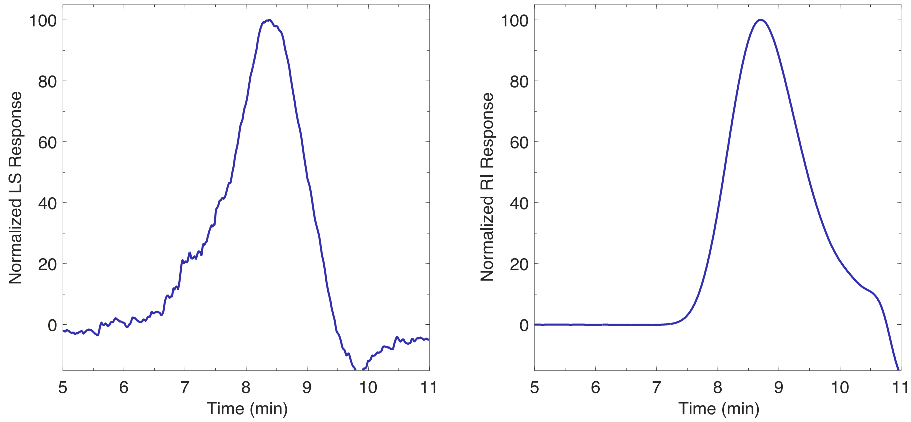

Fig. S34 GPC traces (MALS detector left, RI detector right) corresponding to polymerization in **Table 3**, Entry 5.

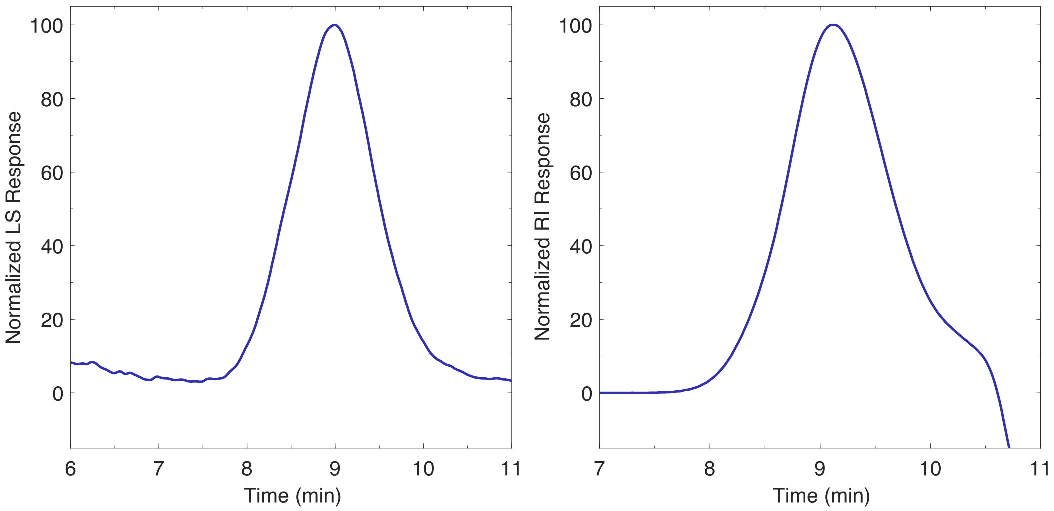

Fig. S35 GPC traces (MALS detector left, RI detector right) corresponding to polymerization in **Table 3**, Entry 6.

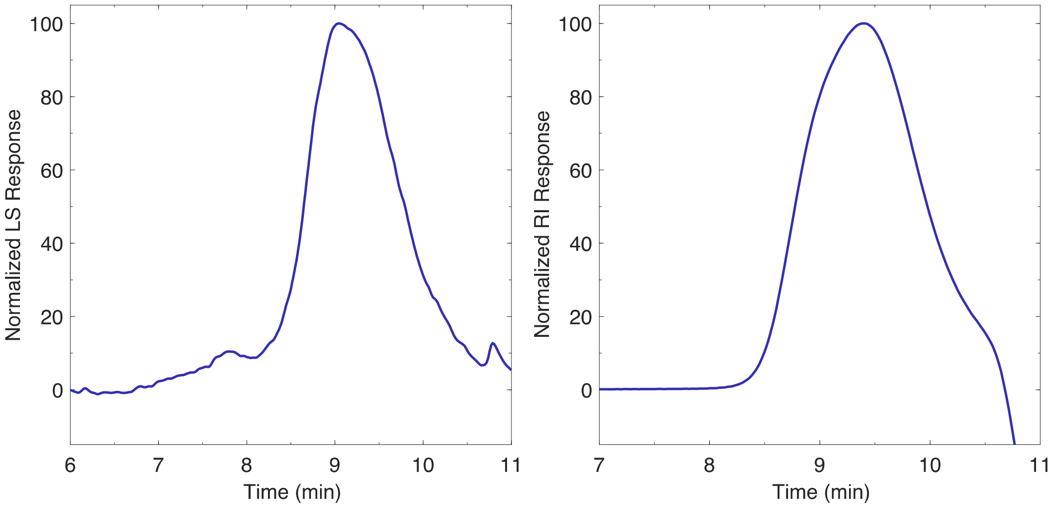

Fig. S36 GPC traces (MALS detector left, RI detector right) corresponding to polymerization in **Table 3**, Entry 7.

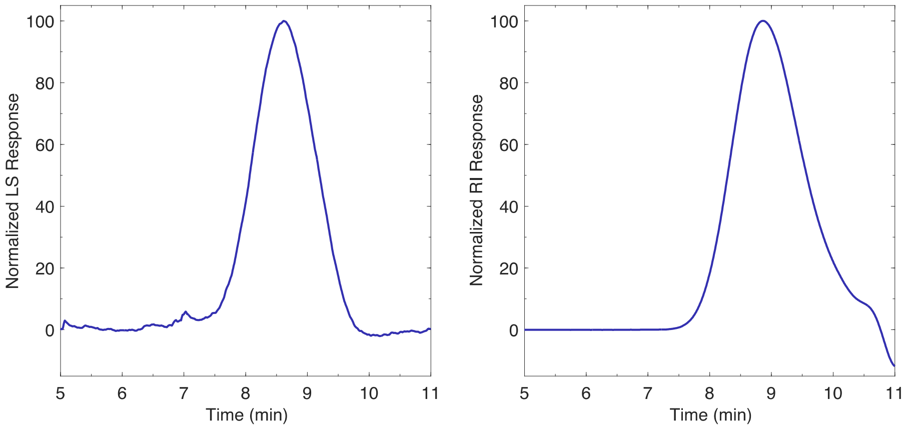

Fig. S37 GPC traces (MALS detector left, RI detector right) corresponding to polymerization in **Table 3**, Entry 8.

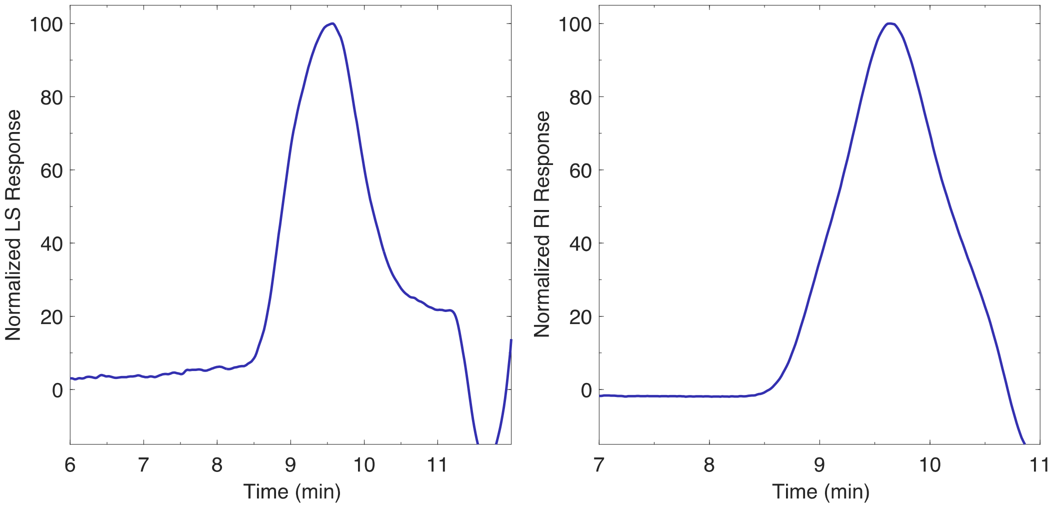

Fig. S38 GPC traces (MALS detector left, RI detector right) corresponding to polymerization in **Table 3**, Entry 9.

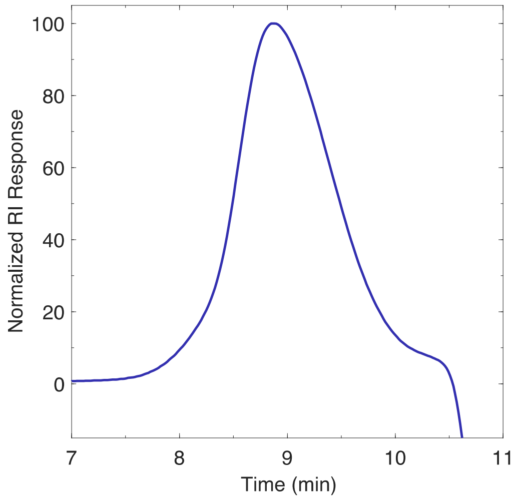

Fig. S39 GPC trace (RI detector) corresponding to polymerization in **Table 3**, Entry 10.

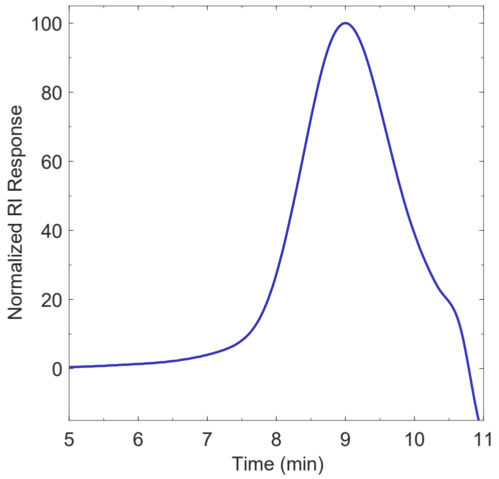

Fig. S40 GPC trace (RI detector) corresponding to polymerization in **Table 3**, Entry 11.

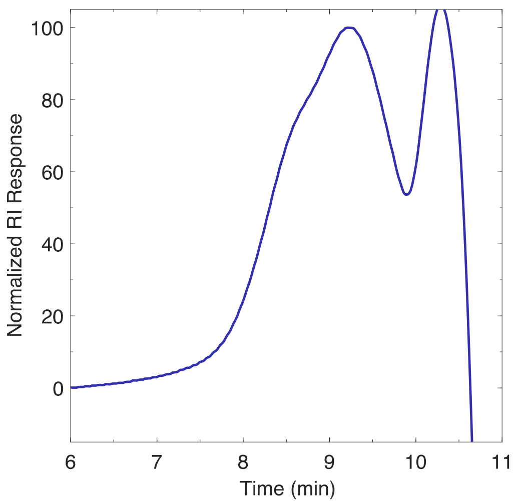

Fig. S41 GPC trace (RI detector) corresponding to polymerization in **Table 3**, Entry 12.

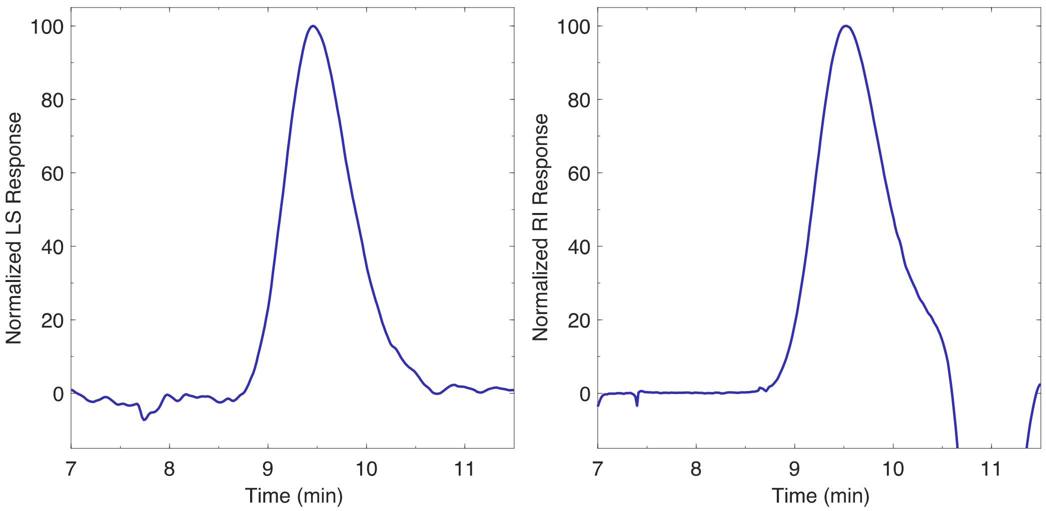

Fig. S42 GPC traces (MALS detector left, RI detector right) corresponding to polymerization in **Table S1**, Entry 10.

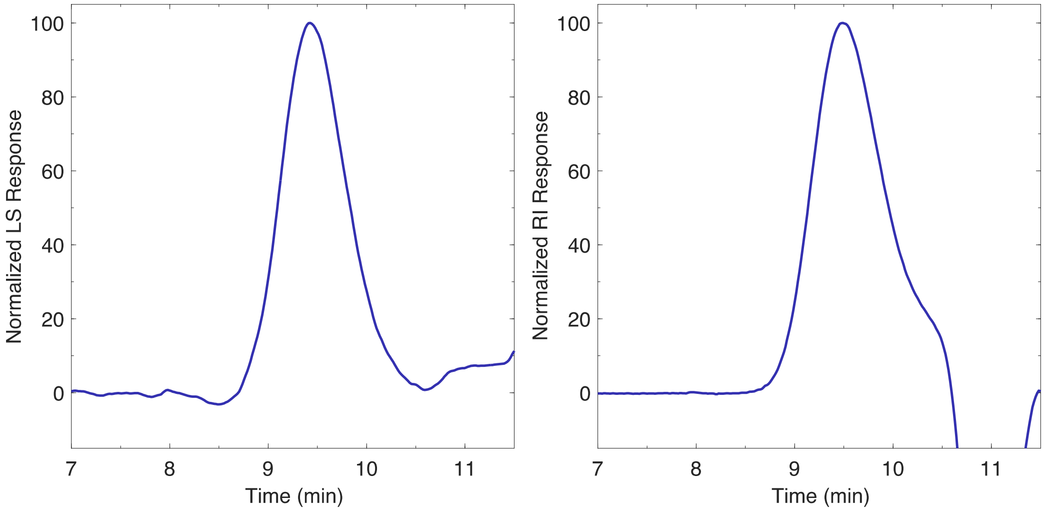

Fig. S43 GPC traces (MALS detector left, RI detector right) corresponding to polymerization in **Table S1**, Entry 11.

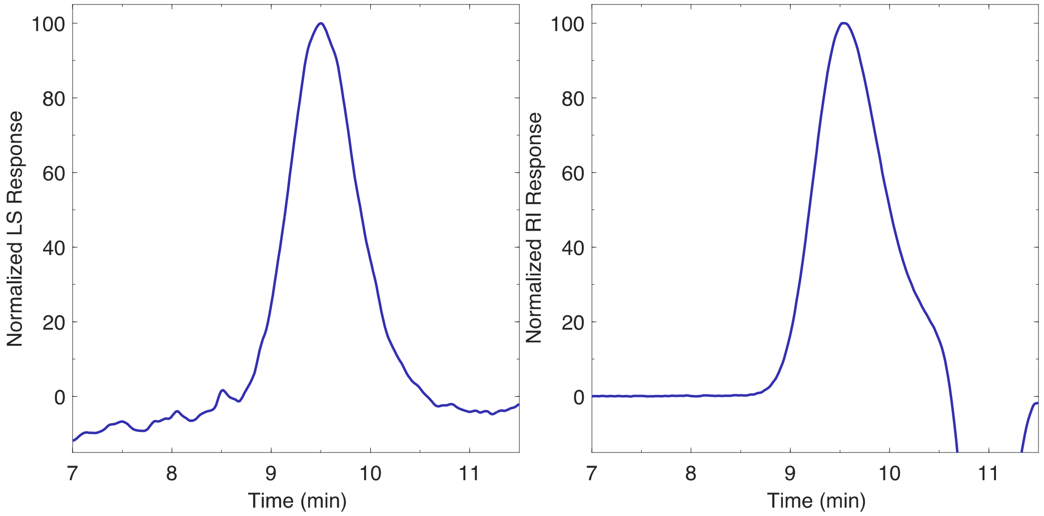

Fig. S44 GPC traces (MALS detector left, RI detector right) corresponding to polymerization in **Table S1**, Entry 12.

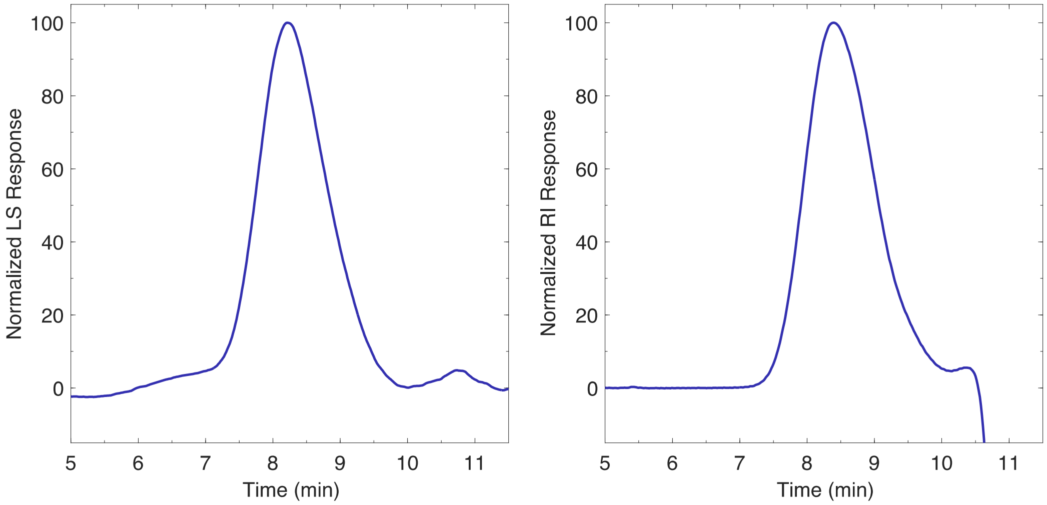

Fig. S45 GPC traces (MALS detector left, RI detector right) corresponding to polymerization in **Table 4**, Entry 2.

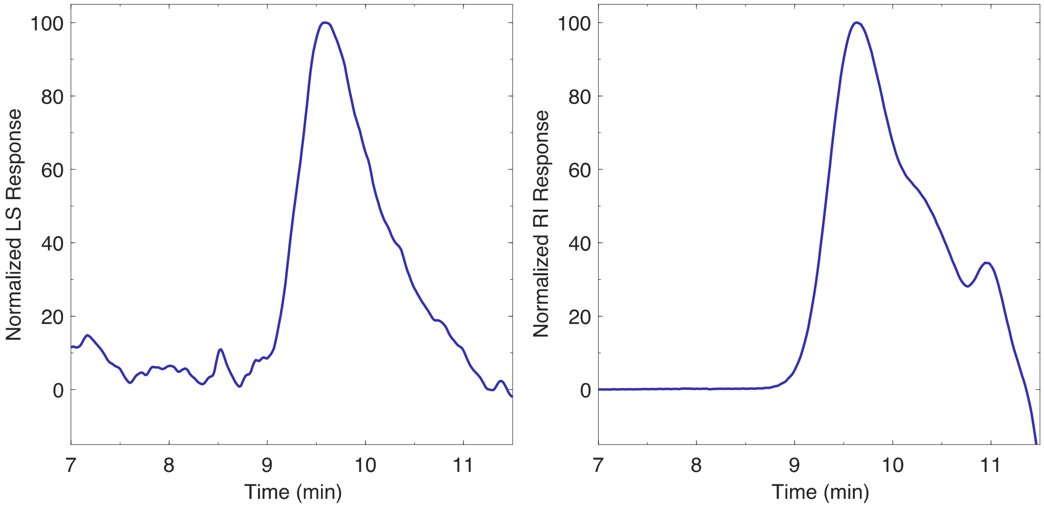

Fig. S46 GPC traces (MALS detector left, RI detector right) corresponding to polymerization in **Table 4**, Entry 3.

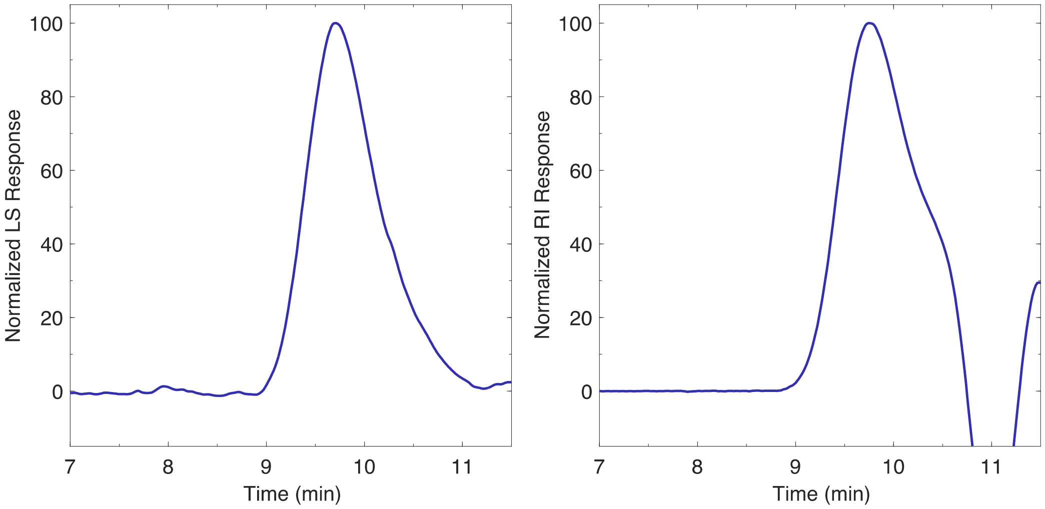

Fig. S47 GPC traces (MALS detector left, RI detector right) corresponding to polymerization in **Table 4**, Entry 4.

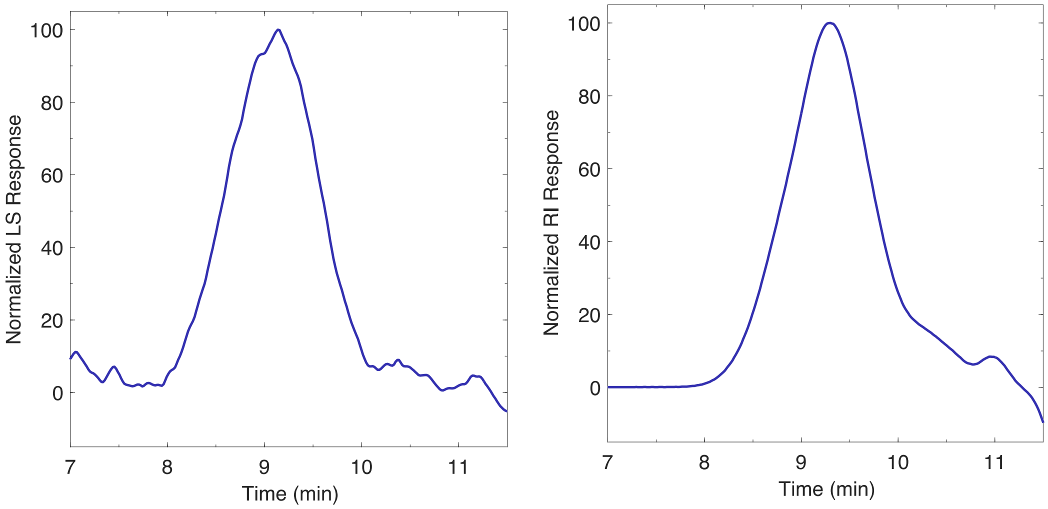

Fig. S48 GPC traces (MALS detector left, RI detector right) corresponding to polymerization in **Table 4**, Entry 5.

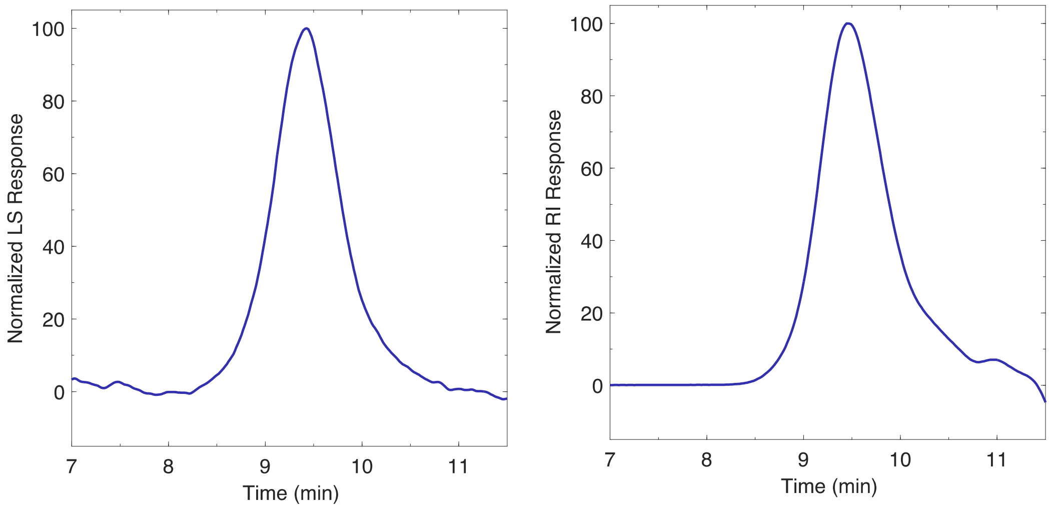

Fig. S49 GPC traces (MALS detector left, RI detector right) corresponding to polymerization in **Table 4**, Entry 7.

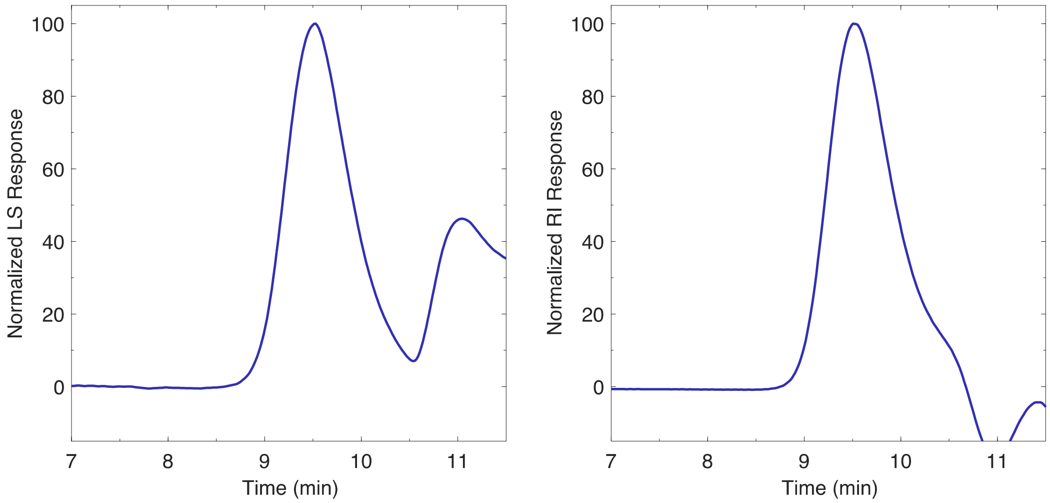

Fig. S50 GPC traces (MALS detector left, RI detector right) corresponding to polymerization in **Table 4**, Entry 9.

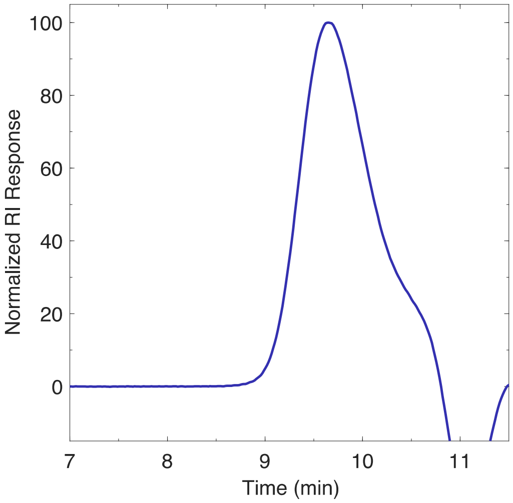

Fig. S51 GPC trace (RI detector) corresponding to polymerization in **Table 4**, Entry 10.

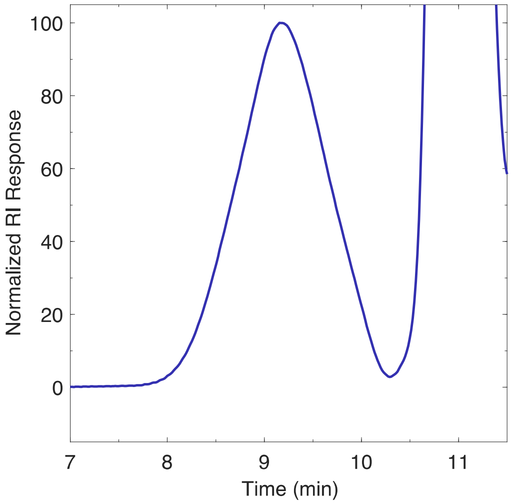

Fig. S52 GPC trace (RI detector) corresponding to polymerization in **Table 4**, Entry 12.

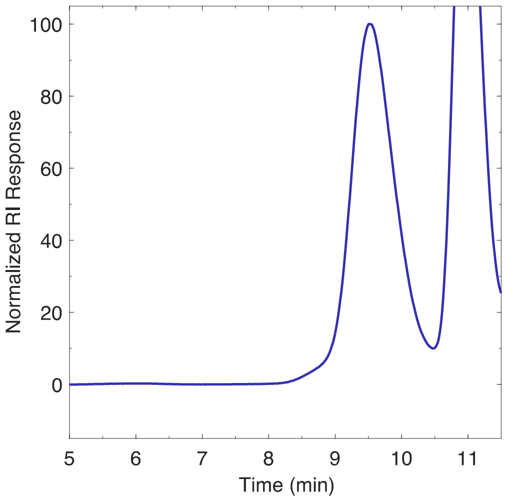

Fig. S53 GPC trace (RI detector) corresponding to polymerization in **Table 4**, Entry 14.

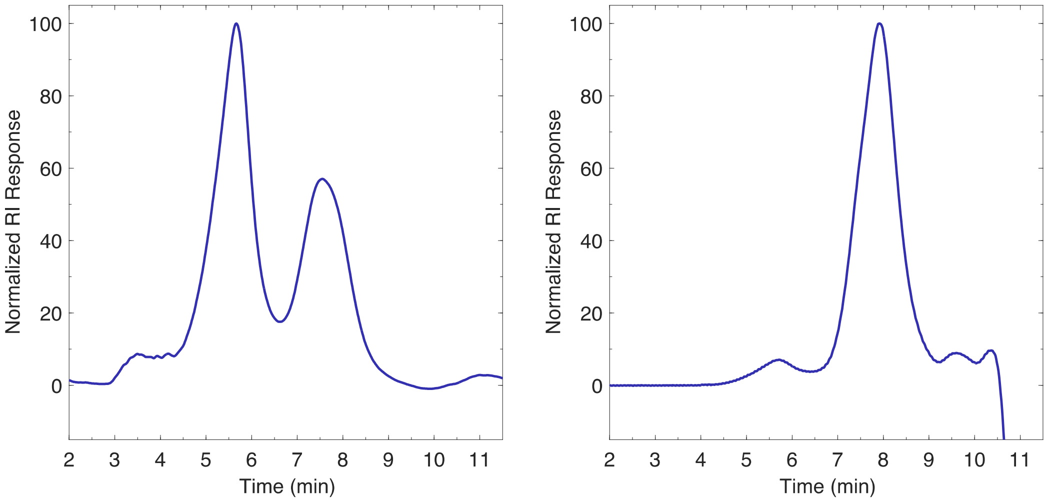

Fig. S54 GPC traces (MALS detector left, RI detector right) corresponding to polymerization in **Table 2**, Entry 1.

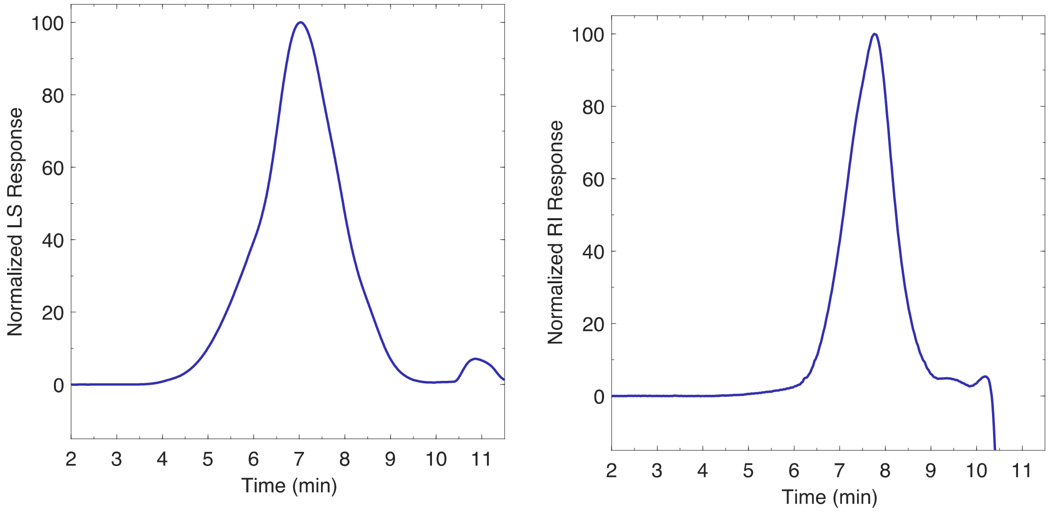

Fig. S55 GPC traces (MALS detector left, RI detector right) corresponding to polymerization in **Table 2**, Entry 2.

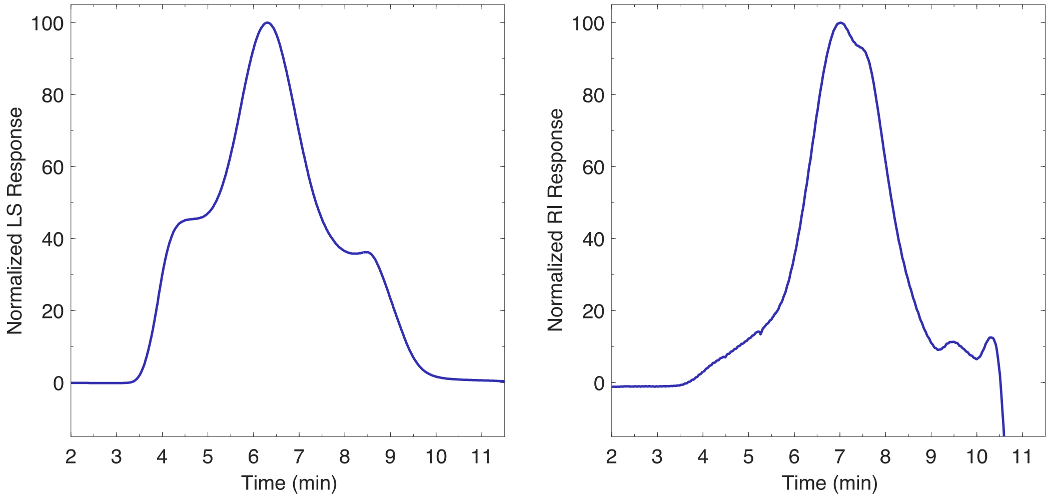

Fig. S56 GPC traces (MALS detector left, RI detector right) corresponding to polymerization in **Table 2**, Entry 3.

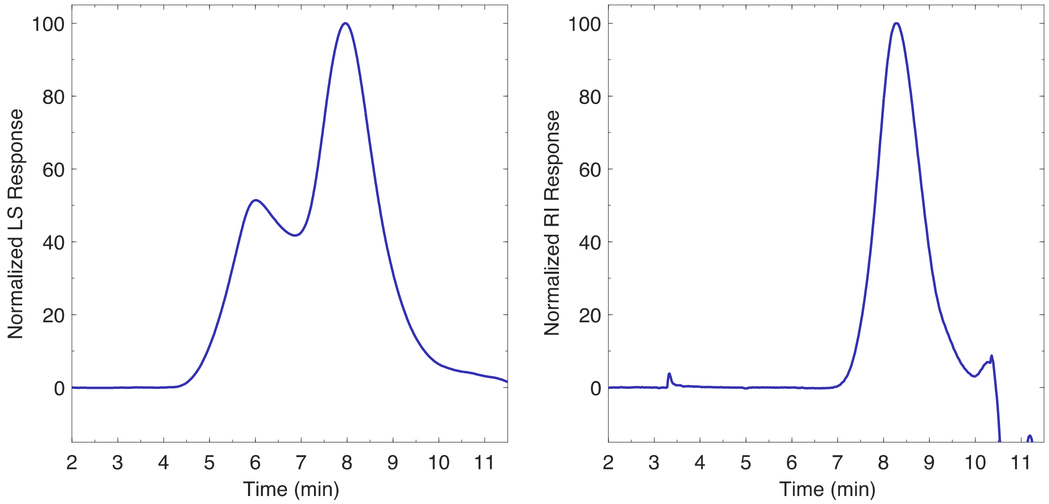

Fig. S57 GPC traces (MALS detector left, RI detector right) corresponding to polymerization in **Table 2**, Entry 4.

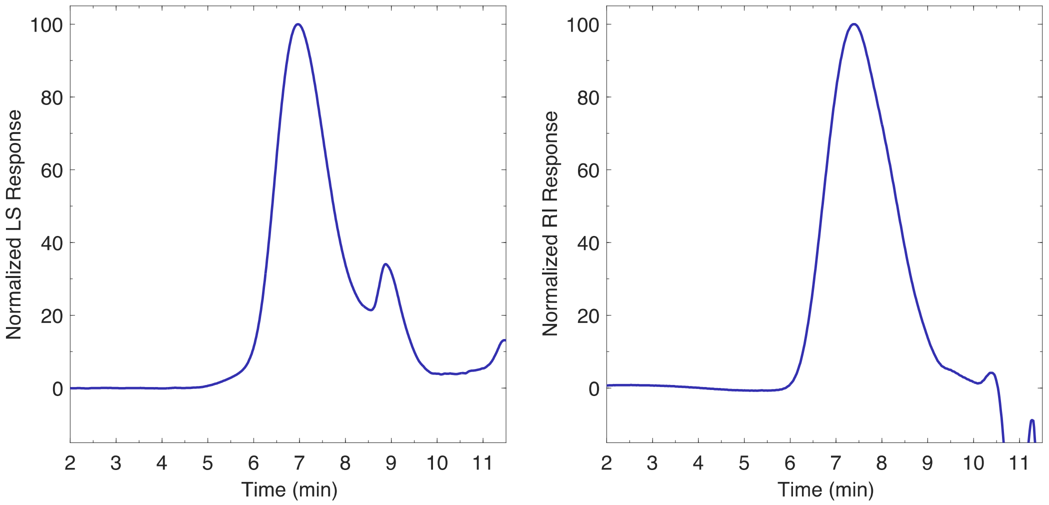

Fig. S58 GPC traces (MALS detector left, RI detector right) corresponding to polymerization in **Table 2**, Entry 5.

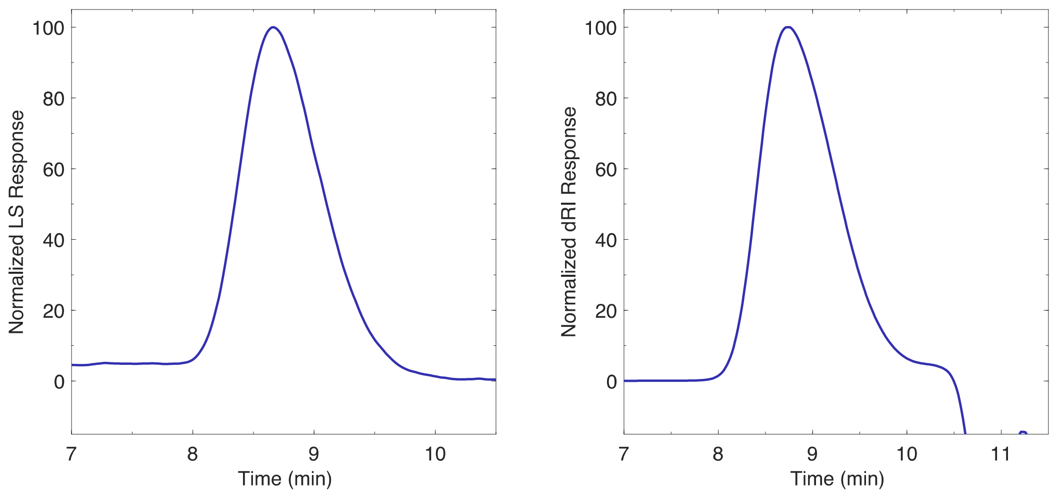

Fig. S59 GPC traces (MALS detector left, RI detector right) corresponding to polymerization in **Table S3**, Entry 1.

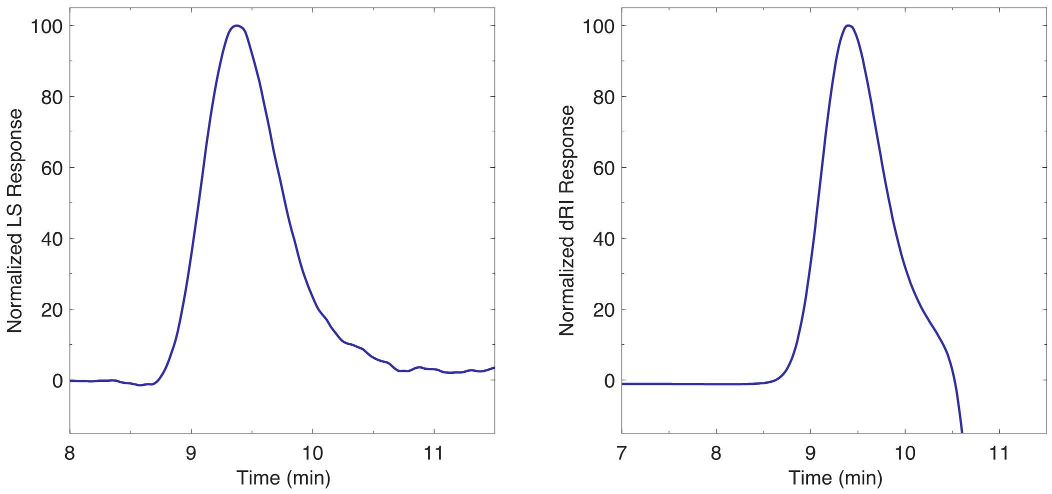

Fig. S60 GPC traces (MALS detector left, RI detector right) corresponding to polymerization in **Table S3**, Entry 2.

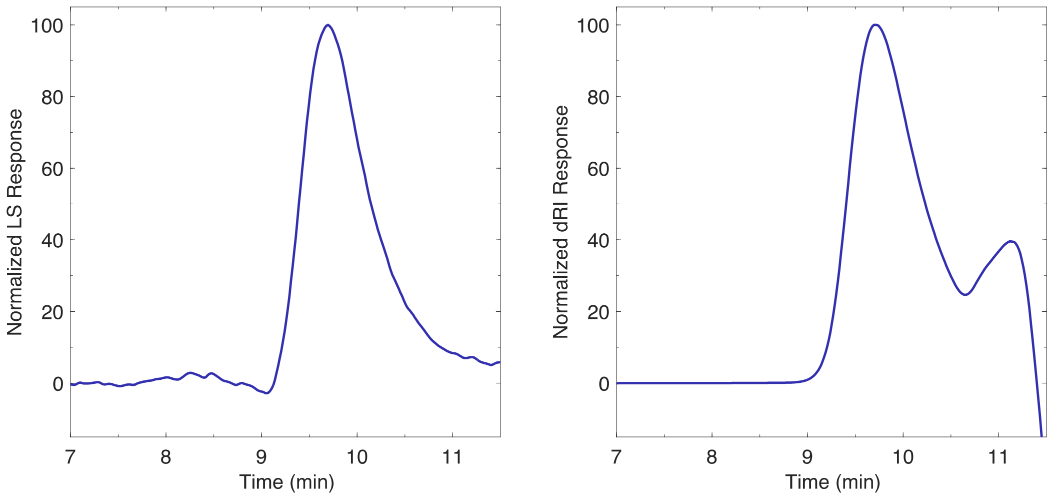

Fig. S61 GPC traces (MALS detector left, RI detector right) corresponding to polymerization in **Table S3**, Entry 3.

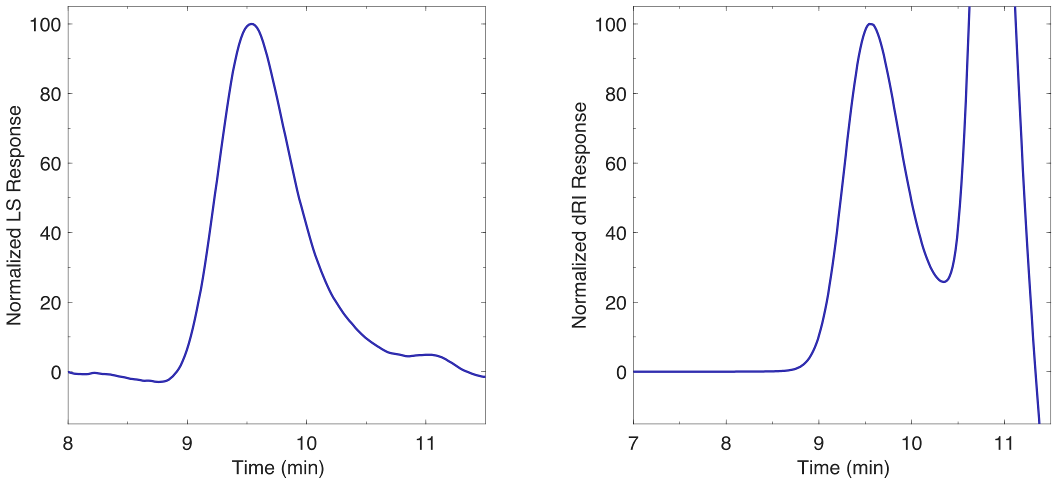

Fig. S62 GPC traces (MALS detector left, RI detector right) corresponding to polymerization in **Table S3**, Entry 4.

7. MALDI-TOF-MS Data

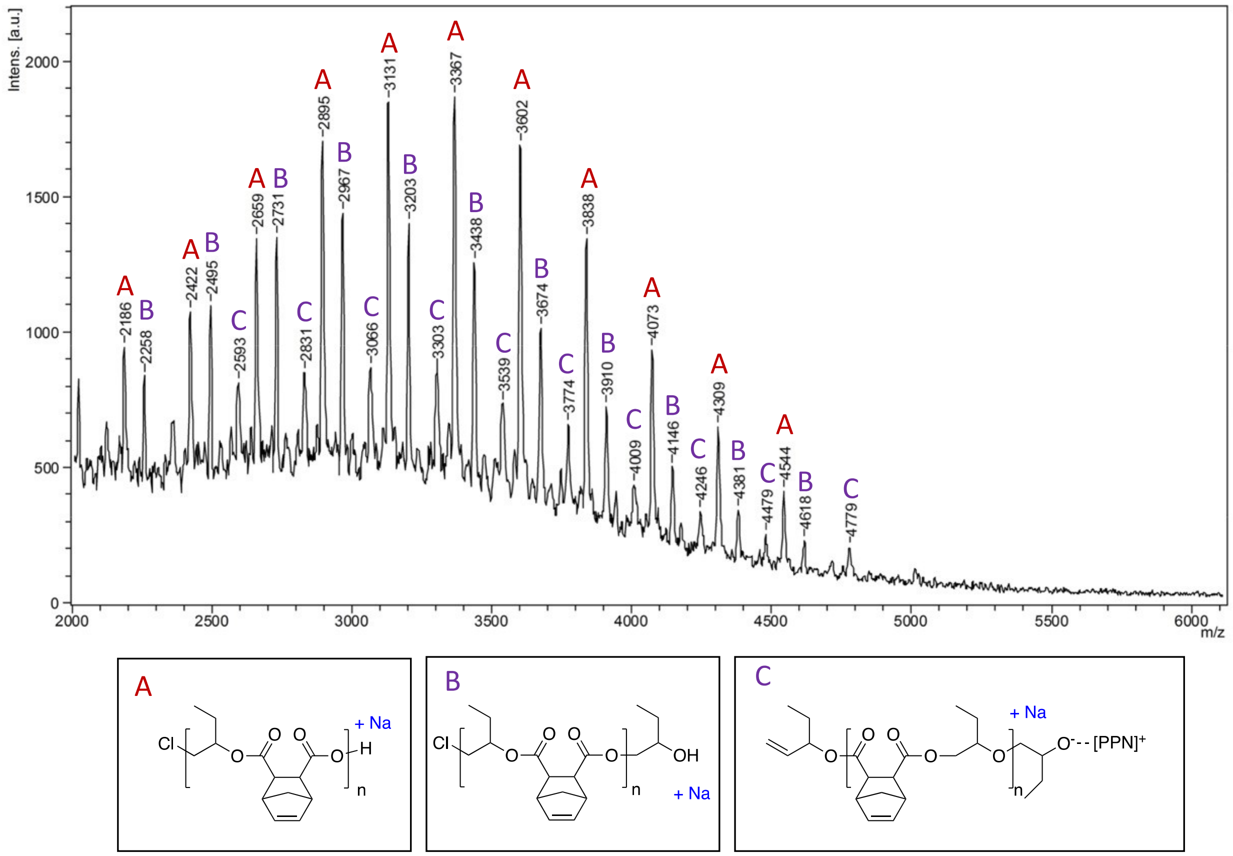

Fig. S63 MALDI-TOF mass spectrum of BO-*alt*-CPMA synthesized using $YCl_3THF_{3.5}$ (Table 1, Entry 2).

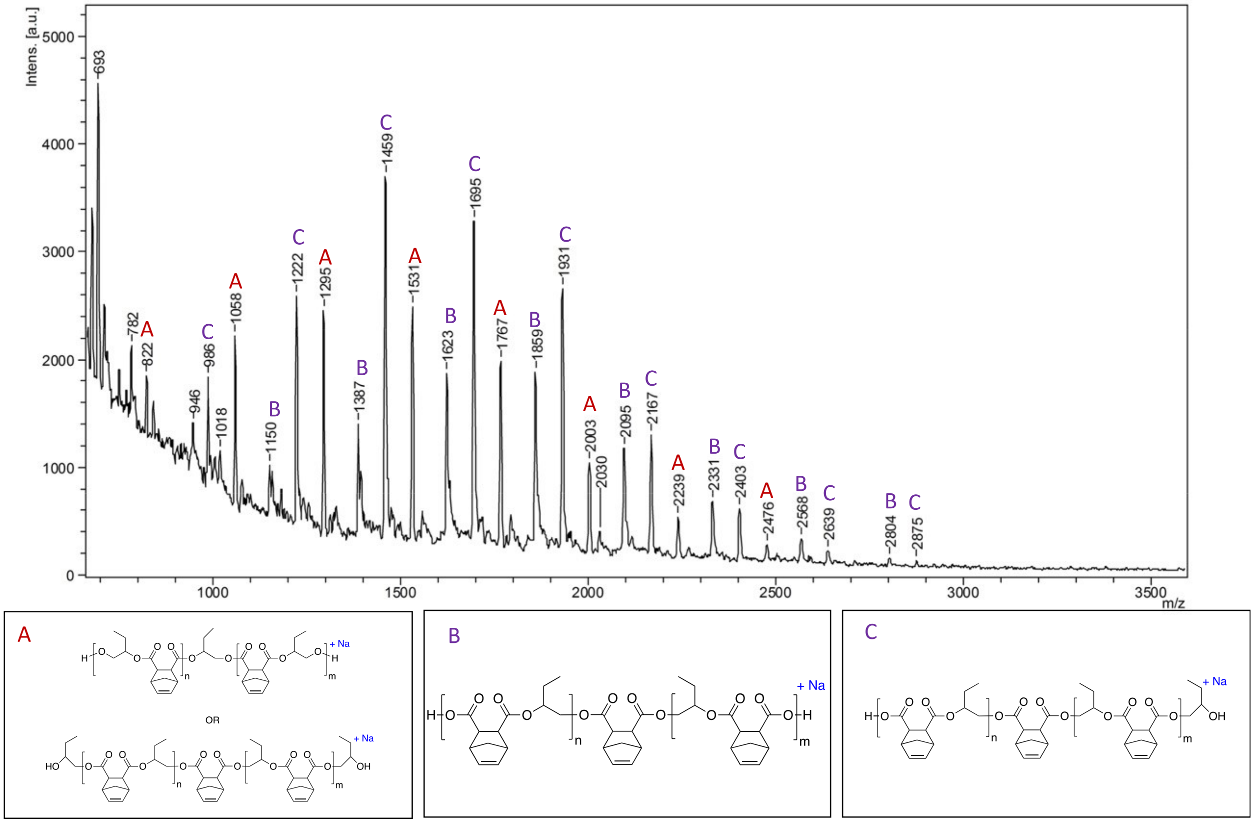

Fig. S64 MALDI-TOF mass spectrum of BO-*alt*-CPMA synthesized using $YCl_3{\cdot}6H_2O$ (Table 4, Entry 1).

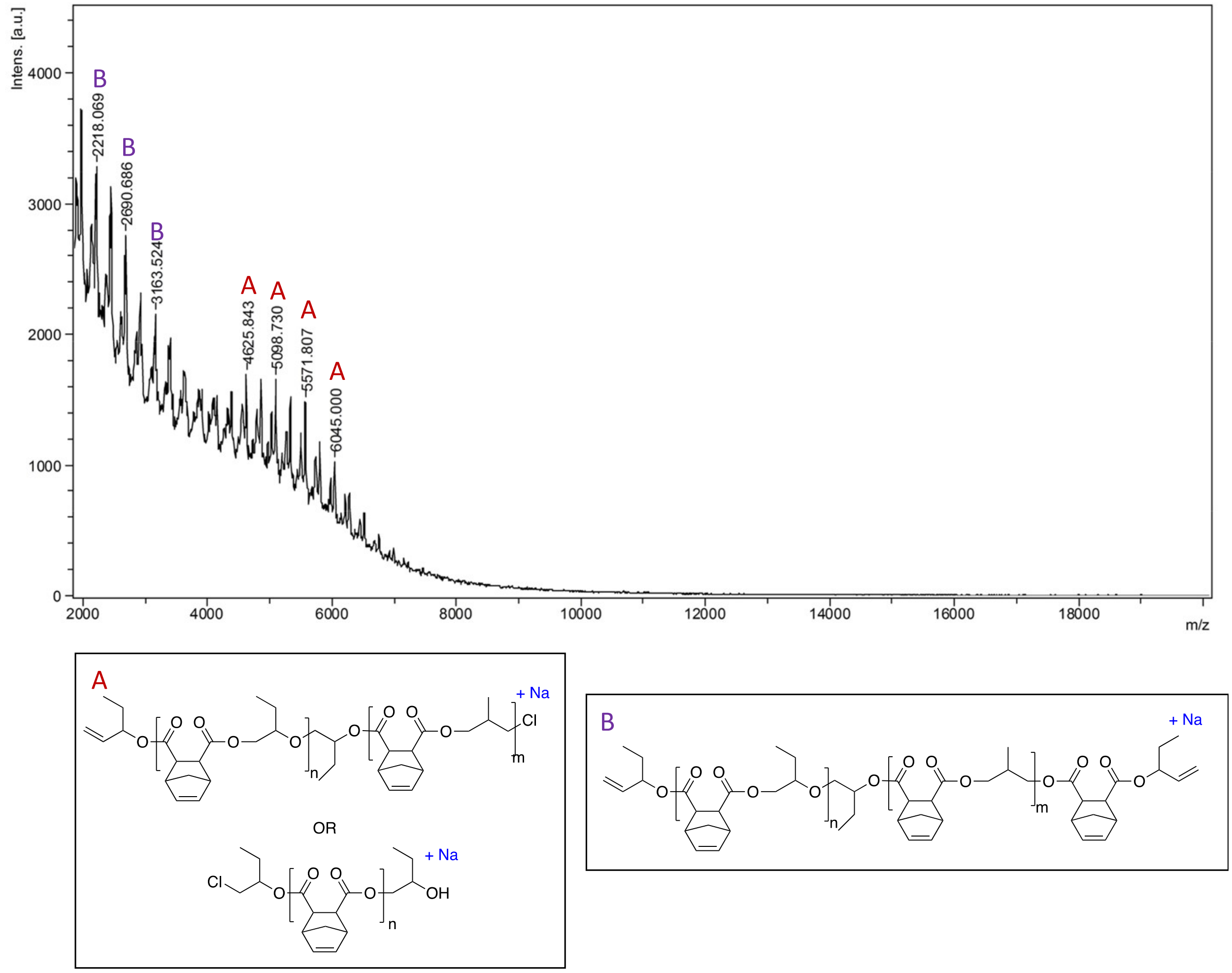

Fig. S65 MALDI-TOF mass spectrum of BO-*alt*-CPMA synthesized using $YCl_3THF_{3.5}$ (Table 1, Entry 5).

In accordance with prior reports by Duchateau, Coates and others,[19-22] the Cl chain end groups for the polymers above could be eliminated during MALDI-TOF-MS characterization, wherein the α- and/or ω-Cl chain end of the catalyst-derived polymer gets transformed to an -ene group. In view of these reports, the MALDI-TOF mass spectrum shown above is suggestive of *α*,*ω*-Cl,Cl end groups.

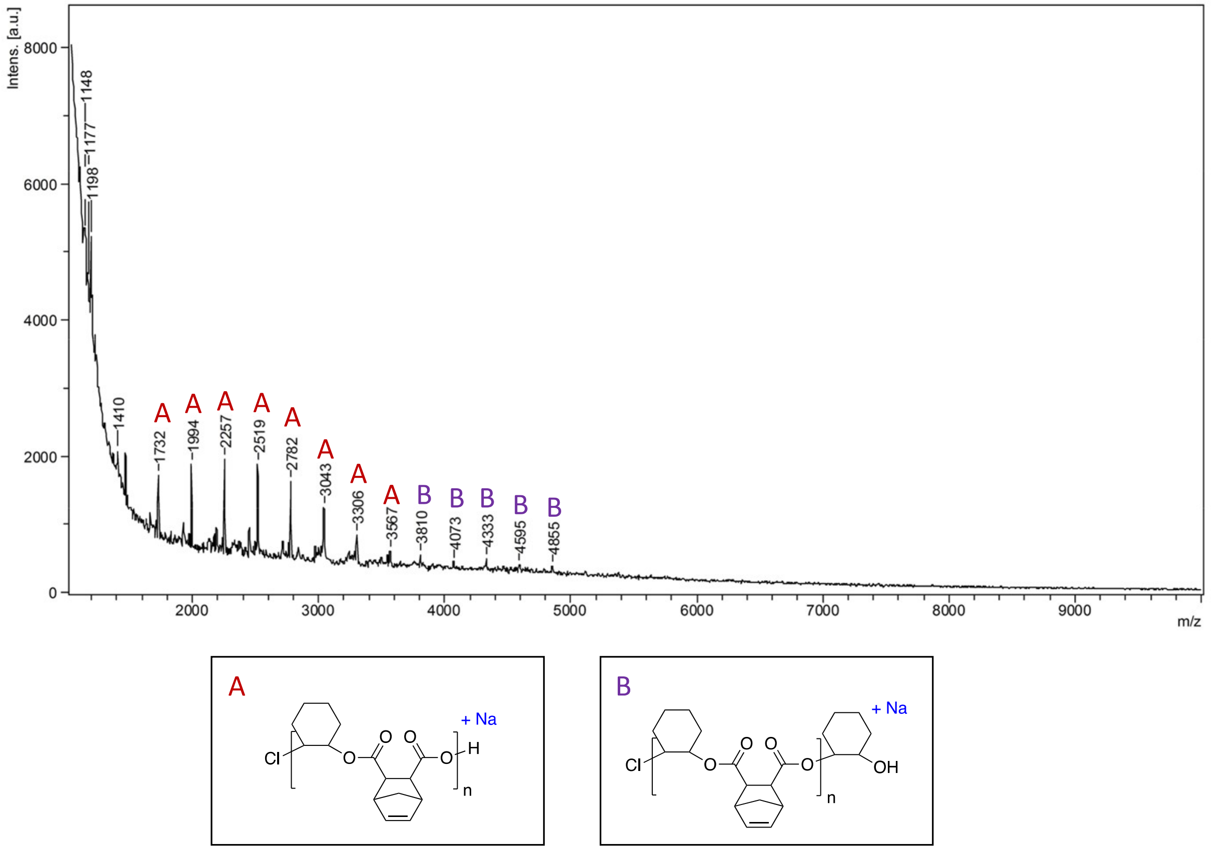

Fig. S66 MALDI-TOF mass spectrum of CHO-*alt*-CPMA synthesized using $YCl_3THF_{3.5}$ (Table 1, Entry 8).

8. Differential Scanning Calorimetry Data

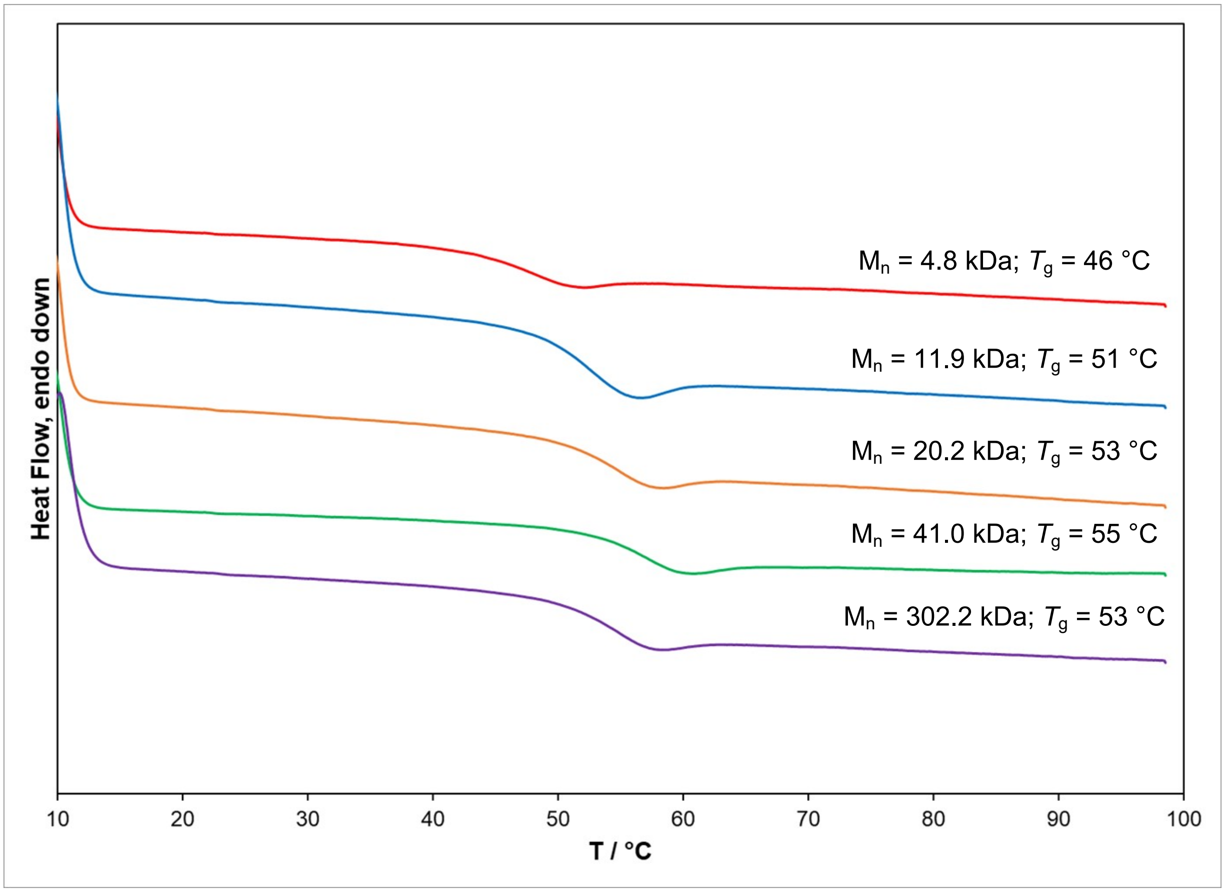

Fig. S67 DSC traces of BO-*alt*-CPMA copolymers synthesized using $YCl_3THF_{3.5}$.

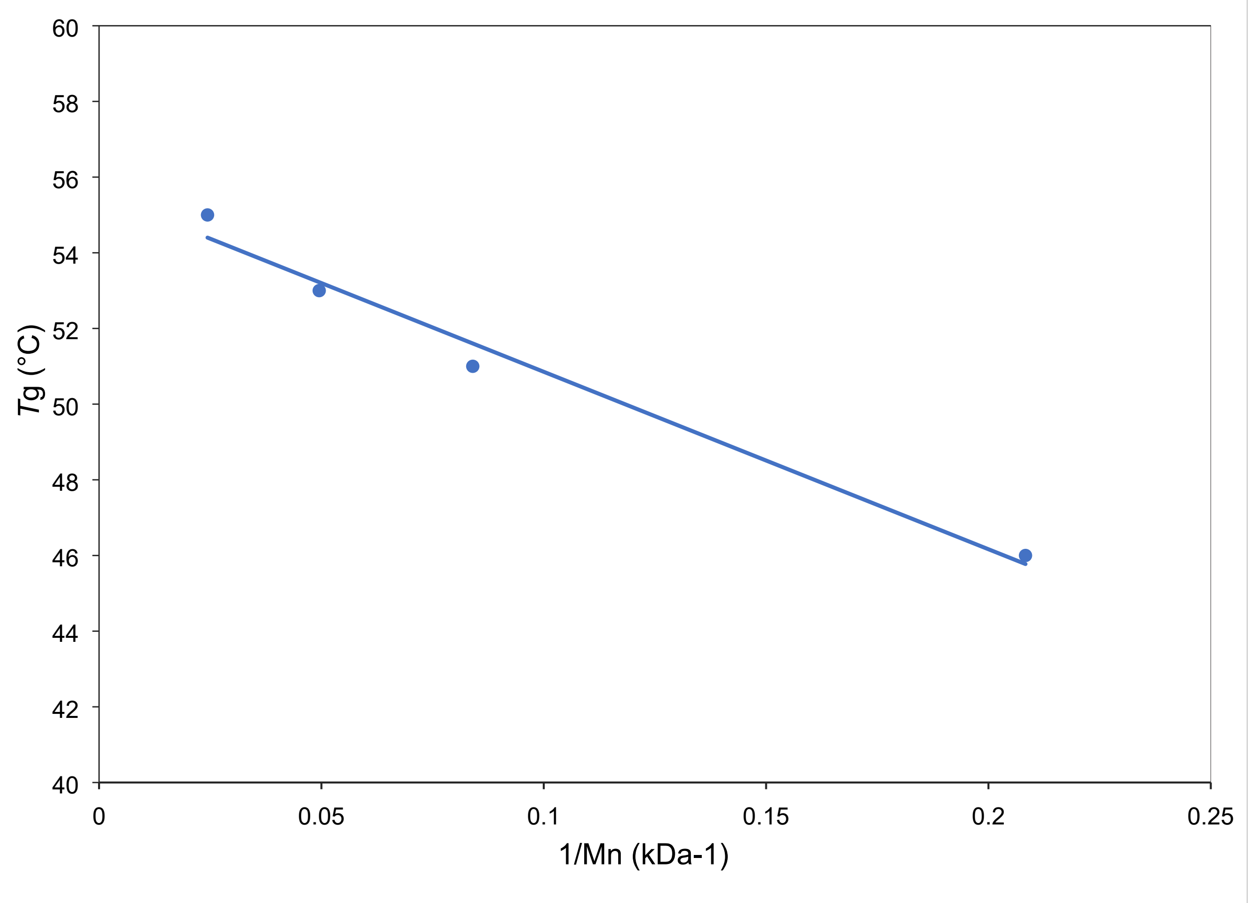

Fig. S68 Dependence of glass transition temperature (T_g) on molecular weight for BO-*alt*-CPMA copolymers synthesized using $YCl_3THF_{3.5}$.

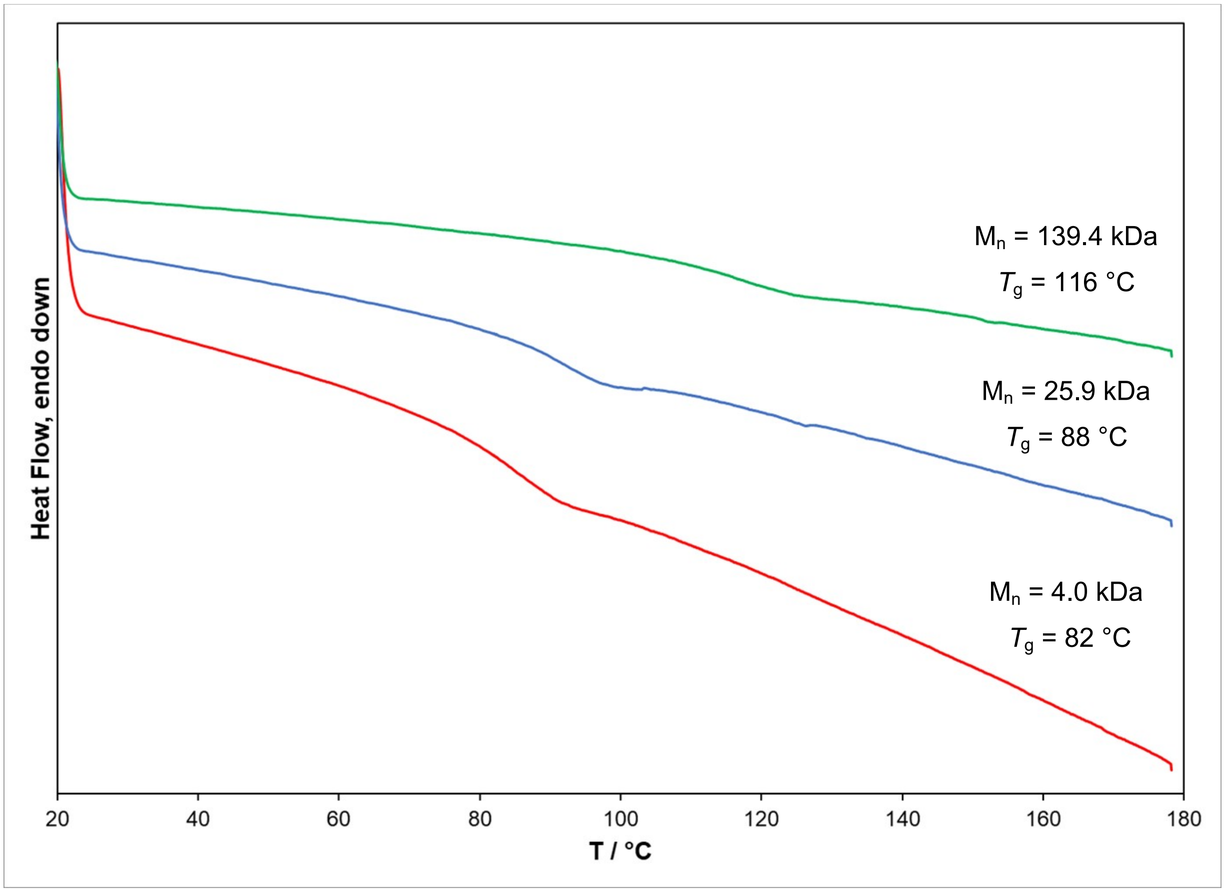

Fig. S69 DSC traces of CHO-*alt*-CPMA copolymers synthesized using $YCl_3THF_{3.5}$.

9. References

[1] P. Sobota, J. Utko, S. Szafert, *Inorg. Chem.,* 1994, **33**, 5203–5206.

[2] M. D. Taylor, C. P. Carter, *J. Inorg. Nucl. Chem.,* 1962, **24**, 387–391.

[3] M. D. Taylor, *Chem. Rev.,* 1962, **62**, 503–511.

[4] B. A. Abel, C. A. Lidston, G. W. Coates, *J. Am. Chem. Soc.,* 2019, **141**, 12760–12769.

[5] L. Bester, A. Bukowska, B. Myśliwiec, K. Hus, D. Tomczyk, P. Urbaniak, W. Bukowski, W. *Polym. Chem.,* 2018, **9**, 2147–2156.

[6] H. -Y. Ji, D. -P. Song, B. Wang, L. Pan, Y. -S. Li, *Green Chem.,* 2019, **21**, 6123–6132.

[7] Z. Hošťálek, O. Trhlíková, Z. Walterová, T. Martinez, F. Peruch, H. Cramail, J. Merna, *Eur. Polym. J.,* 2017, **88**, 433–447.

[8] B. Han, B. Liu, H. Ding, Z. Duan, X. Wang, P. Theato, *Macromolecules,* 2017, **50**, 9207–9215

[9] Y. Liu, J. -Z. Guo, H. -W. Lu, H. -B. Wang, X. -B. Lu, *Marcomolecules,* 2018, **51**, 771–778.

[10] W. T., Diment, G. L. Gregory, A. Buchard, R. W. F. Kerr, A. Phanopoulos, C. K. Williams, *ACS Catal.,* 2021, **11**, 12532–12542.

[11] F. Isnard, M. Lamberti, C. Pellecchia, M. Mazzeo, *ChemCatChem,* 2017, **9**, 2972–2979.

[12] D. A. L. Otte, D. E. Borchmann, C. Lin, M. Weck, K. A. Woerpel, *Org. Lett.,* 2014, **16**, 1566–1569.

[13] B. Han, L. Zhang, B. Liu, X. Dong, I. Kim, Z. Duan, P. Theato, *Macromolecules*, 2015, **48**, 3431–3437.

[14] C.-M. Chen, X. Xu, H.-Y. Ji, B. Wang, L. Pan, Y. Luo, Y.-S. Li, *Macromolecules*, 2021, **54**, 713–724.

[15] J. Y. Jeon, S. C. Eo, J. K. Varghese, B. Y. Lee, *Beilstein J. Org. Chem.*, 2014, **10**, 1787–1795.

[16] J. Zhang, L. Wang, S. Liu, Z. Li, *J. Poly. Sci.*, 2020, **58**, 803–810.

[17] L. -F. Hu, C. -J. Zhang, D. -J. Chen, X. -H. Chao, J. -L. Yang, X. -H. Zhang, *ACS Appl. Polym. Matt.*, 2020, **2**, 5817–5823.

[18] Y. -N. Li, Y. Liu, H. -H. Yang, W. -F. Zhang, X. -B. Lu, *Ang. Chem. Int. Ed.*, 2022, **61**, e202202585.

[19] C. Hu, X. Chen, M. Niu, Q. Zhang, R. Duan, X. Pang, *Macromolecules,* 2022, **55**, 652–657.

[20] W. J. v. Meerendonk, R. Duchateau, C. E. Koning, G.-J. M. Gruter, *Macromolecules,* 2005, **38**, 7306–7313.

[21] M. J. Sanford, L. P. Carrodeguas, N. J. V. Zee, A. W. Kleij, G. W. Coates, *Macromolecules,* 2016, **49**, 6349–6400.

[22] C. A. Lidston, B. A. Abel, G. W. Coates, *J. Am. Chem. Soc.*, 2020, **142**, 20161–20169.
